# Multi-marker comparative analysis of 18S, ITS1, and ITS2 primers for human gut mycobiome profiling

**DOI:** 10.3389/fbinf.2025.1690766

**Published:** 2025-11-19

**Authors:** Hiba Orsud, Sumaya Zoughbor, Fatima AlDhaheri, Khalid Hajissa, Manar Refaey, Suad Ajab, Khaled Alswaider, Nora Mohamed, Obaid Alkaabi, Zakeya Al Rasbi

**Affiliations:** 1 Department of Microbiology and Immunology, College of Medicine and Health Sciences, United Arab Emirates University, Al Ain, Abu Dhabi, United Arab Emirates; 2 Zayed Centre for Health Sciences, College of Medicine and Health Sciences, United Arab Emirates University, Al Ain, United Arab Emirates; 3 Paediatric Department, College of Medicine and Health Sciences, United Arab Emirates University, Al Ain, Abu Dhabi, United Arab Emirates; 4 Department of Genetics and Genomics, College of Medicine and Health Sciences, United Arab Emirates University, Al Ain, United Arab Emirates; 5 Institute of Public Health, College of Medicine and Health Sciences, United Arab Emirates University, Al Ain, United Arab Emirates

**Keywords:** gut mycobiome, ITS sequencing, 18S rRNA, ALDEx2, multi-marker strategy

## Abstract

**Background:**

Gut fungi play crucial roles in human health. The profiling of the human gut mycobiome continues to progress. However, adjustments in the selection of ribosomal DNA marker regions can substantially affect the taxonomic resolution of a population. In particular, the impact of using primers’ combinations is insufficiently defined. In this study, we investigated the performance of three targeted sequencing regions, ITS1, ITS2 and 18S rRNA, separately and in combination.

**Methods:**

Eight fecal samples from healthy individuals (n = 4) and cancer patients (n = 4) were selected as proof of principle for amplicon-based sequencing conducted with the DNBSEQ™ sequencing system. Quality-filtered reads were grouped into operational taxonomic units (OTUs) via USEARCH and categorized using the SILVA (18S) and UNITE (ITS) databases. Downstream bioinformatics encompassed diversity analyses, principal component analysis (PCA), and biomarker detection via linear discriminant analysis effect size (LEfSe). To improve taxonomic coverage and compositional understanding, data were examined using ALDEx2 with centered log-ratio (CLR) transformation, facilitating reliable differential abundance and effect size assessment in small sample metagenomic contexts.

**Results and Discussion:**

Among primers, ITS2 and ITS1 enhanced the coverage of identified taxa, with operational taxonomic unit quantities of 183 and 158, respectively, compared to 58 OTUs of 18S. Accordingly, among primer combinations tested, the triple integration of ITS1–ITS2–18S produced the highest fungal richness, while the dual ITS1–ITS2 combined datasets enhanced group discrimination analysis, showing enrichment of *Candida albican*s and scarcity of *Penicillium sp*. in cancer patients. Our findings based on ITS sequencing and the combination of ITS1 and ITS2 provide instructive information on the composition and dynamics of gut fungi in our initial test subjects, enhancing our understanding of their roles in gut homeostasis and the microbial shifts associated with cancer. Despite our approach being conducted with a limited cohort to establish methodological feasibility, it brings attention to multi-marker strategies, demonstrating that integrated primer datasets surpass traditional single-marker methods in both taxonomic coverage and biomarker detection sensitivity in low-biomass fecal samples. Our research provides a reliable starting point for future studies on gut mycobiome in both healthy and diseased individuals, which could lead to better diagnostics and treatments based on microbiome profiles.

## 1 Introduction

The human gut harbors a complex microbial ecosystem, which is vital for sustaining the host’s health. The interaction between gut microbiota and disease progression remains a focus for researchers. Despite advances in gut mycobiome research, the question of how fungal diversity in stool differs across populations or geographical regions remains poorly defined, with current studies frequently constrained by limited sample sizes, regional focus, or methodological inconsistencies (Nash et al., 2017; Kabwe et al., 2020). Several studies have presented contradictory results concerning the stability and fundamental composition of the gut mycobiome among different populations. Some attribute this variation to experimental conditions, particularly amplicon primer selection and bioinformatics analysis, while the relative impact of additional factors, such as diet, environment, urbanization, ethnicity, and host genetics, on fungal diversity remains controversial (Gupta et al., 2017; Mahnic and Rupnik, 2018).

Advancements in sequencing technologies and computational biology have uncovered the importance of the gut mycobiome, which was previously underappreciated in diversity and significance (Chin et al., 2020). Previous metagenomic sequencing analyses showed that mycobiota constitutes ∼0.03–0.1% of the gut microbiota in healthy populations, primarily consisting of *Saccharomyces, Malassezia*, and *Candida species* (Qin et al., 2010; Gao et al., 2017). Emerging studies found that fungal dysbiosis might be concurrent with disease development and progression, highlighting the importance of precise mycobiome profiling in oncology (Aykut et al., 2019; Liu et al., 2015).

Techniques for fungal community analysis have progressed significantly over the past few decades. While traditional culture-based methods (e.g., microscopy and biochemical assays) were once foundational (Pathan and Patel, 2013), genome sequencing has expanded fungal databases, although they still remain underdeveloped compared to bacterial counterparts (Strati et al., 2016). Despite these advances, detecting fungi in fecal samples remains challenging due to their low biomass, high microbial diversity, and persistent technical limitations.

Using traditional culture-based fungal detection techniques in fecal samples enables rapid clinical diagnostics, but it limits the resolution for low-abundance species and restricts broader applicability due to the uncultivability of numerous fungi, rendering it inadequate for comprehensive mycobiome profiling (Hamad et al., 2017; Sarrabayrouse et al., 2021; Wiesmann et al., 2022). Thereafter, molecular methods revolutionized fungal detection by addressing the limitations of traditional techniques. Primer-based approaches amplify fungal DNA with high sensitivity, enabling species-specific identification and quantification (Huffnagle and Noverr, 2013).

Despite their specificity, primer bias can distort detection accuracy, as evidenced by comparative analyses of commonly used primer pairs (Jana et al., 2013) and further validated in our study. Current methodologies for investigating gut fungal diversity utilize targeted sequencing of specific genetic markers, including the *18S rRNA* gene and the ITS regions, each offering distinct advantages and drawbacks in elucidating fungal community composition of a given sample.

The primary focus on culture-independent analyses and database creation has targeted loci encompassing the *18S*, *5.8S*, and *28S rRNA* genes, along with the ITS1 and ITS2 (Supplementary Figure S1). The analysis of fungal ITS regions along with rRNA genes has uncovered a remarkable variety of fungi within the microbiome (Hoggard et al., 2018a).

The *18S rRNA* gene, which encodes a small ribosome subunit, is sequenced to identify eukaryotes, including fungi. Due to its conserved and phylogenetically informative regions, it is valuable for studying broad taxonomic clades among eukaryotes. Universal primers for *18S rRNA* gene amplification across numerous fungal taxa have been developed based on its highly conserved sequences (Wang et al., 2014). This conservation may not identify closely related species, reducing species-level variation (Schoch et al., 2012a). The conserved *18S rRNA* gene matches sequences across taxa but lacks diversity to identify closely related species or strains, limiting its use in fungal diversification (Pelley and Pelley, 2007). In addition, the *18S rRNA* gene has a higher PCR failure rate than other rRNA markers. This limitation may require group-specific primers to improve amplification of some fungal groups (Banos et al., 2018).

The ITS regions, including flanking subregions of ITS1 and ITS2, are situated between the *18S* and *28S rRNA* genes (Supplementary Figure S1). These locations are more diverse, rendering them suitable for distinguishing closely related fungus species. Thus, the ITS region is proposed as the universal DNA barcode for fungi due to its high variability, and it is beneficial for species-level identification (Takada et al., 2016; Stewart et al., 2007). Public repositories contain ITS region databases with complete sequence data for taxonomic classifications and comparative investigations (Stielow et al., 2015). Therefore, ITS databases are often advantageous for providing valuable resources for fungal identification, but improperly curated reference material might lead to misidentification or overestimation (Nilsson et al., 2008; Gardes and Bruns, 1993; White et al., 1990).

Mycobiome profiling is highly affected by the choice of primers, which can influence taxonomic coverage, detection sensitivity, and community composition outcomes. ITS1, ITS2, and 18S rRNA primers each provide distinct advantages; however, their combined application in fungal profiling has been insufficiently evaluated, particularly in complex, low-biomass samples such as human stool.

The primary objective of this study is to evaluate the performance of ITS1, ITS2, and 18S rRNA primer sets, both individually and in combination, in enhancing fungal community detection and taxonomic resolution. We further aimed to assess the advantages of multi-marker datasets in improving differential abundance detection using appropriate statistical approaches, such as the analysis of differential abundance taking advantage of log-ratios (ALDEx2) R package. It is a compositional data analysis package specifically designed for small-sample microbiome datasets. It employs a centered log-ratio (CLR) transformation tool to accurately estimate differential abundance despite the limited cohort size.

This evaluation was conducted using methodological proof of a limited number of fecal samples from a cohort of cancer patients and cancer-free controls from the Middle East. To tackle controversy over the appropriate gut mycobiome marker (Banos et al., 2018; Takada et al., 2016; Stewart et al., 2007), we evaluated the taxonomic resolution, detection sensitivity, and overall efficacy of each marker utilizing standardized pipelines that incorporate the SILVA and UNITE reference databases. This method was designed to overcome the limitations commonly encountered in prior research, such as reliance on BLAST-based annotations, offering only genus-level identifications, and limited incorporation of comparative or differential abundance analysis regarding the gut mycobiome.

## 2 Materials and methods

### 2.1 Sampling and sequencing

Eight fecal samples (n = 8) were obtained from cancer patients and cancer-free participants for microbiome analysis from a previous study. The samples were selected for a proof-of-concept design to evaluate primer efficacy across different clinical conditions. The cohort consisted of eight adults (ages 42–58), Eastern Mediterranean volunteers (https://www.emro.who.int/information-resources/imemr-database/), including four individuals diagnosed with different malignancies (colorectal, breast, and endometrial) and four cancer-free individuals (Supplementary File S1). Cancer patients were selected for different malignancies, thus providing a range of host conditions for the analysis of gut mycobiome diversity. Individuals free of cancer were paired based on age to decrease confounding due to age-related microbial variability. The purpose of this small yet diverse cohort was to improve methodological understanding of primer efficiency and taxonomic resolution while reducing biological variability that might mask primer-related variations. The smallest sample size (n = 8) was selected since this pilot study aimed not at epidemiological inference but at the technical comparison of primer sets and sequencing efficacy. All samples were obtained under uniform conditions and processed consistently to ensure comparability among groups (Labania et al., 2023). The collected samples were subjected to DNA extraction using the QIAamp PowerFecal Pro DNA Kit (QIAGEN GmbH, Germany). A NanoDrop ND-1000 UV-Vis Spectrophotometer (Thermo Fisher Scientific, Wilmington, DE, United States) was used for DNA purity and concentration inspection, and gel electrophoresis was used for DNA integrity verification. A measure of 30 ng of approved DNA templates was sent to Neo-Science (neoscience.ae) for sequencing; each sample went through three primer-based PCR reactions targeting the *18S rRNA* gene: V4 (5′ to 3′) (F: CCAGCASCYGCGGTAATTCC, R: ACTTTCGTTCTTGAT), ITS1 (F: TCCGTAGGTGAACCTGCGG, R: GCTGCGTTCTTCATCGATGC), and ITS2 (F: GATGAAGAACGYAGYRAA, R: TCCTCCGCTTATTGATATGC) regions. PCR enrichment was performed in 50 μL reactions comprising 30 ng of template DNA using fusion PCR primers, adhering to the specified cycle conditions: 95 °C for 3 min, 30 cycles of 95 °C for 15 s, 56 °C for 15 s, and 72 °C for 45 s, followed by a final extension at 72 °C for 5 min. PCR products were purified utilizing DNA magnetic beads (BGI, LB00V60). Library preparation was conducted using 2× Phanta Max Master Mix (VAZYME, China). The resultant libraries were subjected to circularization and rolling circle amplification to produce DNA nanoballs (DNBs), which were subsequently deposited onto patterned nanoarrays. Sequencing was conducted on the DNBSEQ-G400 platform (BGI-Shenzhen, China) utilizing paired-end 300 bp reads.

### 2.2 Bioinformatics analysis workflow

Raw sequencing data underwent preliminary quality control for contaminants to ensure clean data for analysis. The merging of overlapping paired-end reads into contiguous tags was completed using FLASH v1.2.11, which finalized the filtering process. UCHIME (v4.2.40) was used for chimera detection, and USEARCH (v7.0.1090) clustered high-quality reads into operational taxonomic units (OTUs). Detailed information on DNA concentration and quality assessment, sequencing quality control metrics, OTU statistics, and software used for clustering and chimera detection is provided in Supplementary File S1.

The 18S rRNA database, SILVA v.138 for 18S rDNA amplicon sequencing OTUs, and UNITE v.8.2, a fungal-specialized database, were utilized for taxonomic classification. Based on the OTU profile table and taxonomic annotation results for each approach, R (v4.4.1) libraries (detailed in Supplementary File S2 were used to conduct species accumulation and prevalence rate analyses, alpha diversity, beta diversity, and differential principal component analysis (PCA), log2-fold differential abundance evaluations, and linear discriminant analysis effect size (LEfSe) to identify biomarkers and gain insights into microbial community composition and structure (RStudio. bookdown, 2023; Microbiome Project, 2023; Bioconductor, 2023; Abarenkov et al., 2025).

To improve taxonomic coverage, datasets were combined from ITS1 and ITS2, ITS1 and 18S, ITS2 and 18S, and all primer datasets collectively, and OTU tables along with their accompanying taxonomy annotations were integrated into a singular dataset for each combination of ITS1–ITS2, ITS1–18S, ITS2–18S, and ITS1–ITS2–18S. Furthermore, the combined datasets and singular datasets (ITS1, ITS2, and 18S) were subjected to ALDEx2, which is appropriate for small metagenomic sample sizes. This method applied CLR-transformed abundance matrices that underwent PCA using the ALDEx2 v. 1.36.0 package to investigate compositional variation across groups. Subsequently, we evaluate differential abundance and effect size estimation. A step-by-step protocol for bioinformatics analysis is provided in Supplementary File S2. All analytical reproducible code scripts are provided in a step-by-step protocol (File S2) and accessible in our GitHub repository: GitHub https://github.com/HibaOrsud/18S-Microbiome-.git and Zenodo (DOI: 10.5281/zenodo.17198284).

### 2.3 Ethics statement

This study was conducted on previously collected samples, which received approval from the Tawam Human Research Ethics Committee at Tawam Hospital, Al Ain, Abu Dhabi, United Arab Emirates. Ethical approval was provided from 25 December 2019 to 31 March 2021 (approval no. THREC-678). Informed consent was obtained from participants before enrollment. All procedures adhered to the ethical standards given by the institutional research committee, according to Good Clinical Practice (GCP) guidelines, the Department of Health (DoH), Abu Dhabi, and the 1964 Declaration of Helsinki, together with its subsequent revisions or equivalent ethical standards.

## 3 Results

The results provided an in-depth characterization of fungal communities across limited samples and primer sets, highlighting significant differences in richness, diversity, and taxonomic composition. The study revealed substantial group-specific patterns and emphasized the influence of primer selection, along with the combination of their datasets, on community profiling.

### 3.1 OTU distribution across primer sets

**FIGURE 1:**
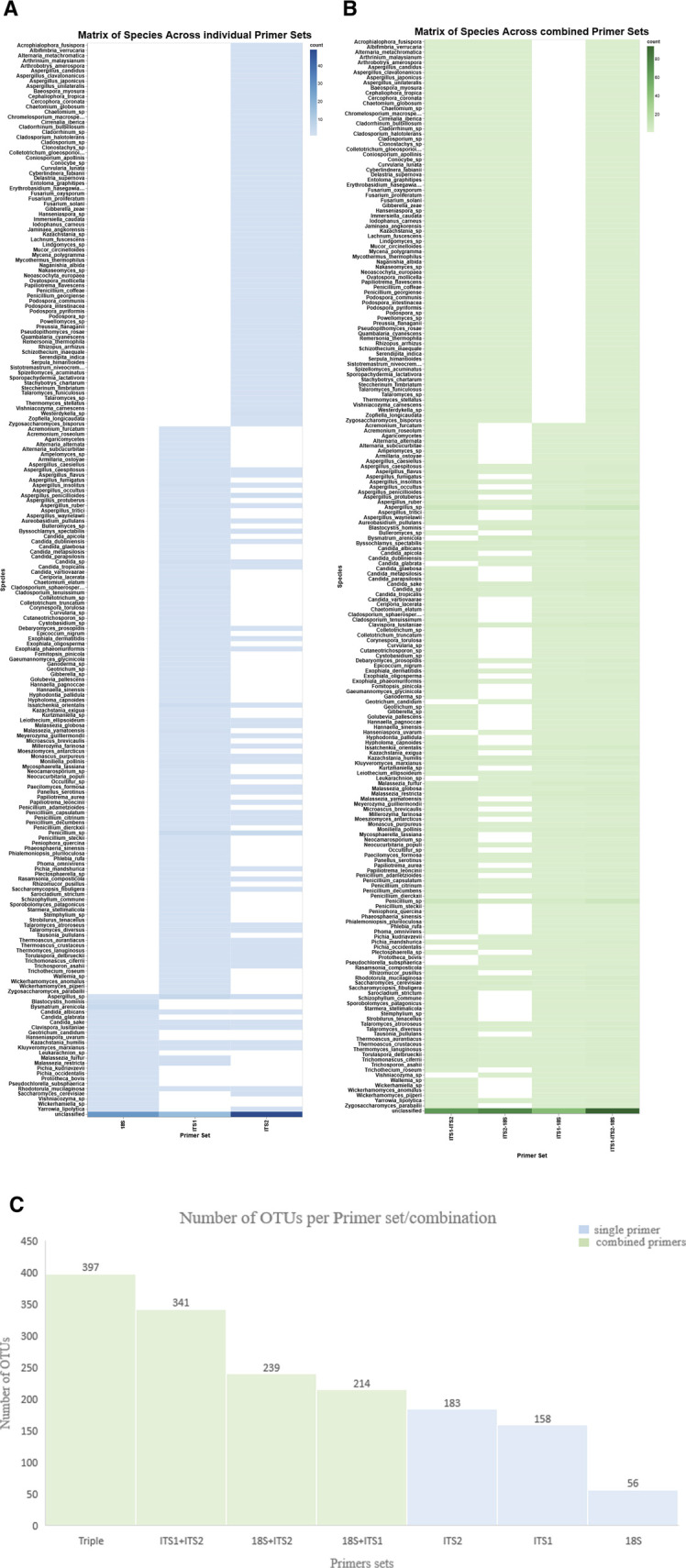
Matrix of species and OTUs across individual and combined primer sets. The heatmaps show species detection across individual and combined primer sets. X-axis: individual primer sets **(A)** (ITS1, ITS2, and 18S) and combined primer sets **(B)**. Y-axis: species, the hue color map represents the detection level, indicating increased or decreased species abundance. **(C)** The bar chart displays the number of OTUs generated from each single primer set (blue) and in combination (green).

**FIGURE 2:**
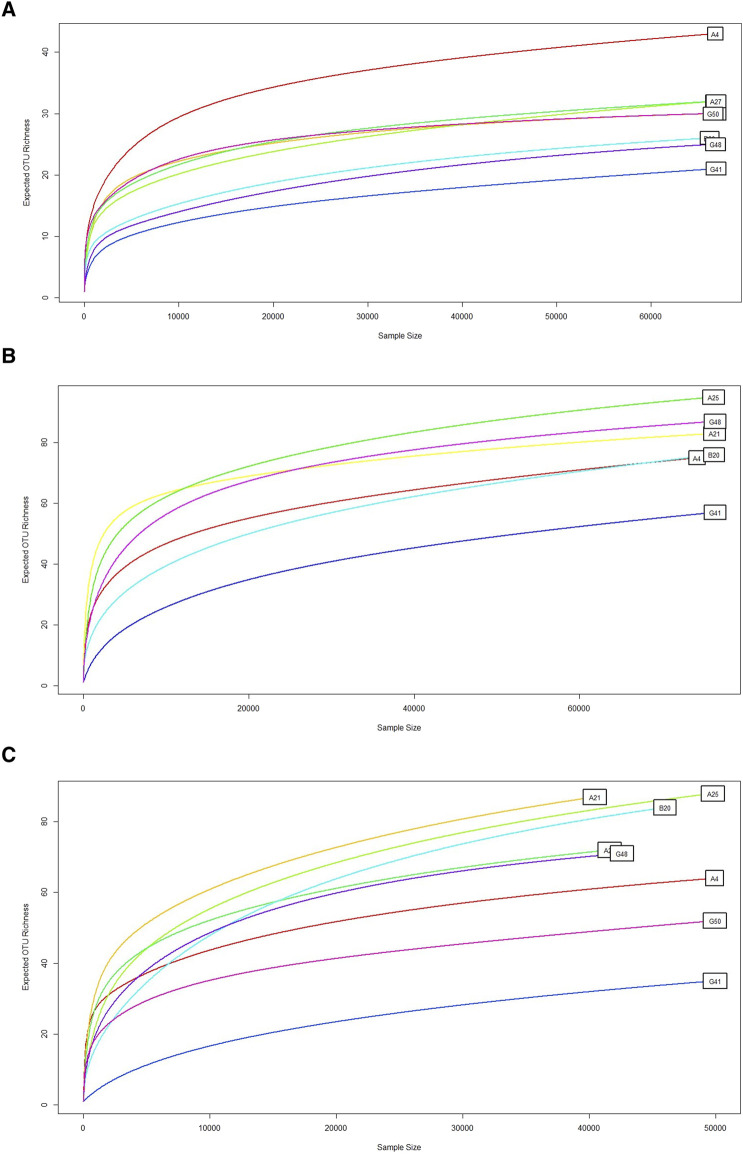
Rarefaction curve. This graph shows the rarefaction curve for microbial diversity using **(A)** 18S rRNA, **(B)** ITS1, and **(C)** ITS2 primers, demonstrating the expected OTUs richness (y-axis) relative to the number of samples or sequencing depth (x-axis). A consistent curve indicates adequate sequencing depth. The expected OTU richness increased with the sample size, which was typical of rarefaction curves. Some samples, such as G50, G41 and G48, showed a lower increase in OTU richness. Conversely, some samples, such as A4, A25, and B20, quickly saturated. Overall, ITS1 and ITS2 represent the higher OTUs richness. However, 18S sequencing produced remarkably low OTUs richness.

The variation in OTU richness across the three primer sets was observed by assessing the quantity of OTUs using three primer sets—18S, ITS1, and ITS2—among eight samples. A bar plot illustrating OTU counts demonstrated significant heterogeneity in richness among the samples and primer sets (Supplementary Figure S2a). Both ITS1 and ITS2 primers consistently yielded a higher number of OTUs (n = 158 and n = 183, respectively) than the 18S primer (n = 56). Accordingly, the histogram enhanced comprehension of microbial detection, illustrating the frequency distribution of OTUs across the primer sets (Figure 2b). On the other hand, the triple-combined dataset ITS1–ITS2–18S represented a greater number of OTUs (n = 397) than the pairwise combined ITS1–ITS2 (n = 341), ITS2–18S (n = 239), and ITS1–18S (n = 214). Moreover, but it also enabled the detection of a wider array of fungal taxa, showing the beneficial effect of primer set integration in enhancing taxonomic resolution (Figure 1). Detailed OTU counts and distributions are provided in Supplementary File S3.

### 3.2 Sequencing depth evaluation via rarefaction analysis

To evaluate read accumulation per sample, the created rarefaction curves assessed sequencing depth that adequately captured the microbial diversity within each sample. The ITS1 and ITS2 primers surpassed 18S in capturing OTU richness across samples (Figure 2). ITS2 exhibited the highest diversity and the slowest saturation, rendering it very effective for evaluating varied fungal or eukaryotic microbial populations. Conversely, the 18S primer, although beneficial for broader taxonomic representation, may exhibit constraints in resolution and sensitivity. This difference (Table 1) indicates varying ecological complexities among samples and underscores the necessity of tailored sequencing procedures based on community richness, such as the multi-marker strategy.

**TABLE 1:** Cross-primer comparison of OTU richness and rarefaction trends.

Primer set	Maximum OTU richness %	Saturation trend
18S	∼45	Early plateau
ITS1	∼75	Moderate
ITS2	∼85	Late plateau

OTU richness (%) reflects the highest number of OTUs captured per primer set across all samples. The saturation trend indicates how quickly the rarefaction curve flattens, reflecting whether the sequencing depth was sufficient to capture most of the diversity. The data were derived from rarefaction analyses conducted in R4.0 (vegan2.6 and ggplots3.2.0 packages). The results indicate that ITS2 performed better in detecting a wide range of fungal taxa, while 18S reached saturation earlier and revealed lower diversity.

### 3.3 Taxonomic prevalence patterns

To assess variation in microbial abundance across taxa, log-transformed prevalence profiling revealed significant differences in microbial family-level abundance among the samples. Notable fungal families include Aspergillaceae, Saccharomycetaceae, and Pichiaceae, which exhibited consistently high abundance across many samples when using ITS1 and ITS2 individually, compared to the 18S primer, increasing the potential of ITS-based sequencing for detecting the dominance and opportunistic growth of relevant conditions (Supplementary Figure S3). Furthermore, species-level profiles of taxa prevalence and abundance offer deeper insights into the microbial composition of each sample (Supplementary Figure S4). These visualizations elucidated major differences in community distribution within samples and among primer sets, highlighting the heterogeneity of ITS primers in contrast to 18S. For instance, while many species were consistently present, others were markedly sample-specific, corroborating the identification of group-specific OTUs (Supplementary Figure S5). The uniformity of these trends in both family- and species-level analysis further reinforces the validity of our findings.

### 3.4 Differential abundance profiles per individual primer

**FIGURE 3:**
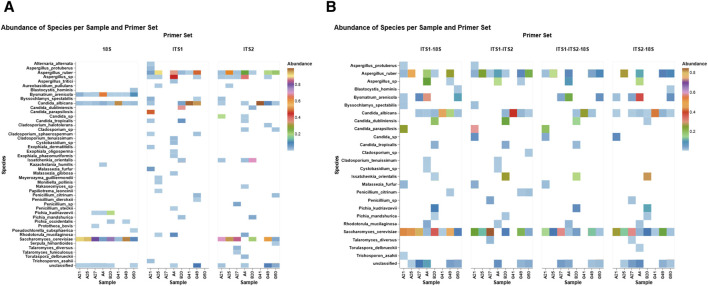
Differential abundance profiles per primer set. The heatmaps illustrate the relative abundance of fungal and eukaryotic species across eight fecal samples with three primer sets (18S, ITS1, and ITS2) **(A)** and primer combinations (ITS1–18S, ITS2–18S, ITS1–ITS2, and ITS1–ITS2–18S) **(B)**. Each tile signifies the sample-specific abundance of a discovered taxon, with color intensity reflecting the abundance level (0%–1%). The color gradient signifies abundance, emphasizing primer-dependent detection sensitivity and specificity within fungal and non-fungal gut populations.

A differential abundance heatmap was created to evaluate primer-specific profile patterns across eight fecal samples using three primer sets: 18S, ITS1, and ITS2 (Figure 3a). The heatmap demonstrated considerable variations in taxonomic recognition among primers, emphasizing both shared and distinct fungal profiles. The ITS primer sets exhibited the highest taxonomic coverage, identifying multiple taxa with mostly elevated relative abundance, such as *S. cerevisiae* (45%), *Aspergillus* spp. (14%), *C. albicans* (22%), and *Candida* sp. (8%), which were captured by ITS2. Similarly, ITS1 detected *C. albicans* (34%), *A.* spp. (16%), uniquely detected *Malassezia* spp. (18%) and *Exophiala* spp. (1.03%), and exhibited differential detection relative to ITS2. Conversely, the 18S primer set detected non-fungal eukaryotes (e.g., *Blastocystis hominis* and *Bysmatrum arenicola*) and yeast (e.g., *Kazachstania humilis*) that were unique to 18S, indicating its broader eukaryotic detection range. On the other hand, 18S detected *C. albicans* (13%) and *S. cerevisiae* (57%) in all eight samples consistently. However, ITS detected both species in selected samples. Furthermore, *Candida parapsilosis* (48%) and *Candida dubliniensis* (20%) were distinctively captured by ITS1, and *Candida tropicalis* (2%) was consistently detected across both ITS primers.

### 3.5 Differential abundance in combined primer sets

The differential abundance patterns of the top fungal species identified in eight fecal samples, detected by four primer set combinations: ITS1–18S, ITS1–ITS2, ITS1–ITS2–18S, and ITS2–18S (Figure 3b). Considerable differences in detection sensitivity and taxonomic range are apparent among the primer sets. For example, ITS1–ITS2 exhibited elevated detection rates for *Candida* spp. and *A.* spp., with increased relative abundances in particular samples. The triple combination of ITS1–ITS2–18S consistently identified core taxa such as *S. cerevisiae* and *Rhodotorula mucilaginosa* in all subjects, demonstrating a comprehensive profiling of both prevalent and infrequent fungi. Conversely, ITS1–18S and ITS2–18S exhibited a slightly narrower taxonomic profile while still identifying notable species such as *Trichosporon asahii* and *Talaromyces diversus.*

#### 3.5.1 Case studies

This protocol was conducted as a proof-of-principle trial to examine gut fungi in stool samples from two groups: CG, comprising cancer patients (n = 4), and CF, encompassing cancer-free individuals (n = 4). The study highlights human gut mycobiome profiling, facilitated by combined primer strategies for modifying the workflow for evaluating the performance of ITS1, ITS2, and 18S datasets.

The distribution of OTUs was assessed through Venn diagrams of the two piloted groups for each primer set to evaluate group-specific microbiological trends. The results evidently illustrate the enhanced discriminatory power of the ITS primer sets in distinguishing between the groups, particularly ITS2, in delineating group-specific microbial diversity. Among all tested primers, ITS2 detected the highest total number of OTUs (n = 183) including 31 OTUs specific to the cancer group (Supplementary Figure S5; Supplementary File S3).

The differential abundance analysis using the three primer sets (ITS1, ITS2, and 18S) demonstrated diverse profiles in the detection of fungal species and group-specific enrichment (Supplementary Figure S6). In our trial cohorts, ITS1 indicated that *C. albicans* and *Aspergillus ruber* were commonly prevalent in the cancer group (CG), but *Malassezia furfur* and *C. dubliniensis* were present at moderate levels, predominantly in the cancer-free (CF) group. ITS2 further improved species-level resolution, emphasizing the significant enrichment of *C. albicans* in CG and *S. cerevisiae* in CF. This primer additionally identified taxa such as *Penicillium* sp. and *Issatchenkia orientalis*, enhancing discriminating power among groups. Conversely, 18S exhibited a wider eukaryotic diversity, with *S. cerevisiae* predominating in the CF group and *C. albicans* maintaining prominence in the CG group. Nevertheless, 18S yielded a greater quantity of unclassified taxa, indicating reduced selectivity for fungal community characterization compared to ITS primers.

The integration of primer datasets improved the inferentiality of abundantly variable fungal taxa between the CF and cancer (CG) groups (Supplementary Figure S7). In the ITS1–ITS2 combination, *C. albicans* and *A. ruber* were significantly more prevalent in the CG group, corroborating a trend previously noted in the individual ITS1 dataset. In contrast, *S. cerevisiae* was consistently more prevalent in CF samples, especially in the ITS1–ITS2 and ITS1–18S pairings. The ITS1–18S and ITS2–18S combinations revealed a broader spectrum of unclassified taxa, particularly in CG. This suggests that a greater portion of the mycobiota might be hidden and potentially dysbiotic in the cancer cohort. The triply integrated ITS1–ITS2–18S dataset provides the most thorough perspective, preserving the detection of significant fungal taxa such as *C. albicans* and *S. cerevisiae* and expanding coverage of rare or unclassified species. Therefore, the integration of datasets together provides the most comprehensive view, integrating signals from all gene regions and reinforcing trends observed in primer combinations (Table 2).

**TABLE 2:** Comparative table of the generally observed trends (combined vs. individual dataset-derived heatmaps).

Species	Combined datasets (ITS1–ITS2, ITS1–18S, ITS2–18S, and triple combined)	Individual datasets (ITS1, ITS2, and 18S)
*Saccharomyces cerevisiae*	Higher abundance in CF (especially in ITS1–ITS2 and ITS2–18S)	Higher abundance in CF in 18S; moderate in ITS2
*Candida albicans*	Higher in CG (especially ITS1–ITS2 and ITS2–18S)	Higher in CG across all datasets
Unclassified Basidiomycota	Higher in CG, particularly in ITS2–18S	Not observed
*Pichia kudriavzevii*	Not observed	Appears in CG in the 18S dataset
*Candida parapsilosis*	Present in CF (ITS1–ITS2, all combined)	Present in the CF of ITS1
*Penicillium* sp.	Slight increase in CG with ITS2–18S	Low abundance in the CG of ITS2
Unclassified species	Higher in CG, especially in ITS2–18S	Evenly distributed in both CF and CG with more abundance in 18S

CF, cancer-free participants; CG, cancer group.

#### 3.5.2 Linear discriminant analysis

We examined the combined potential of various ribosomal markers because of the differential abundance approaches for the individual and integrated datasets of 18S, ITS1, and ITS2. LEfSe analysis quantified these differences, identifying taxa with significant discriminatory power based on their linear discriminant analysis (LDA) scores. Each primer set enabled the identification of group-specific fungal species; however, ITS1 and ITS2 yielded a greater number of informative indicators. For instance, *Aspergillus* sp. (LDA = 3.7 and *p* = 0.031) and *Penicillium* sp. (LDA = 2.8 and *p* = 0.032) were prominently detected with ITS2, while *M. furfur* (LDA = 3.9 and *p* = 0.025) and *C. parapsilosis* (LDA = 4.2 and *p* = 0.023) were more evident with ITS1. *Candida albicans* demonstrated substantial differential abundance across both ITS primer sets (ITS1: LDA = 4.7 and *p* = 0.026; ITS2: LDA = 4.5 and *p* = 0.04). In contrast, the 18S rRNA dataset revealed fewer species with meaningful effect sizes, such as *K. humilis* (LDA = 2.5 and *p* = 0.021), with many of the dominant taxa remaining unclassified, reflecting its limited taxonomic resolution at the species level (Supplementary Figure S8).

Upon the dataset’s integration, the differentiation ability increased notwithstanding the elevation of *p*-value (Supplementary Figure S9). For instance, *C. albicans* (LDA = 4.5 and *p* = 0.08), *C. parapsilosis* (LDA = 4.1 and *p* = 0.8), and *C. dubliniensis* (LDA = 3.8 and *p* = 0.4) were consistently enriched in CG across the dataset combination, and CF was characterized by *Aspergillus rube* (LDA = 4.5 and *p* = 0.1), *Penicillium* sp. (LDA = 2.0 and *p* = 0.08), and *K. humilis* (LDA = 2.2 and *p* = 0.02). Although 18S inclusion broadened the taxonomic coverage, it diminished fungal resolution and increased dataset complexity. Although the combined dataset revealed a stronger differential abundance capturing *C. albicans* as a biomarker (LDA = 4.58), the statistical significance decreased to *p* = 0.08 compared to LEfSe analyses using separate primers (*p*-adj <0.05). This indicates increased within-group variance and expanded taxonomic coverage characteristic of multi-marker integration, emphasizing the necessity of capturing both biological relevance and statistical robustness; nonetheless, this requires further statistical evaluation due to increased variation, including additional tuning or stratification of ALDEx2 LDA analysis (Table 3).

**TABLE 3:** Comparative table of differentially abundant key taxa across primer approaches.

Taxon	ITS1–ITS2	ITS1–18S	ITS2–18S	Triple combined (ITS1–ITS2–18S)
*Candida albicans*	CG (very strong)	CG (strong)	CG (moderate)	CG (High effect, p-adj moderate)
*Candida parapsilosis*	CG (strong)	CG (weaker)	CG (low)	CG (strong)
*Candida dubliniensis*	Not detected	CG (strong)	Not detected	CG (present)
*Saccharomyces cerevisiae*	CF (strong)	CF (present)	CF (moderate)	CF (consistent)
*Penicillium* spp.	CF (strong)	CF (weaker)	CF (weaker)	CF (strong)
*Kazachstania humilis*	Not detected	CF (moderate)	CF (moderate)	CF (detected)
*Blastocystis hominis*	Not detected	CG (low)	CG (low)	CG (detected)
Eukaryotic Noise/Unclassified	Minimal	Present	Moderate	Moderate

CF, cancer-free participants; CG, cancer group.

#### 3.5.3 Integration of ALDEx2 LDA for improving statistical confidence

**FIGURE 4:**
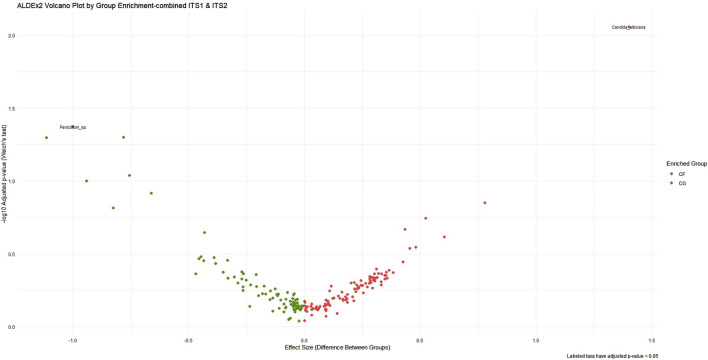
ALDEx2 volcano plot of differential abundance across ITS1–ITS2 combined primer dataset. This volcano plot visualizes the differential abundance of microbial taxa between cancer-free (CF, green) and cancer (CG, red) groups using ALDEx2. The x-axis represents the effect size, while the y-axis shows the statistical significance (−log_10_ adjusted *p*-value). Taxa farther from the origin and higher on the plot are both highly differentially abundant and statistically significant. The plot emphasizes taxa that are enriched in each group, aiding in the identification of biologically meaningful and robust microbial biomarkers.

ALDEx2’s LDA was employed concurrently to harmonize biological relevance (via LEfSe) with statistical significance (through ALDEx2), facilitating a more refined interpretation of taxa exhibiting substantial impacts yet varied certainty. As a result, the pairwise combined primer sets ITS1–ITS2 had the most significant effect size range and yielded the most distinct group differentiation, particularly in detecting fungal dysbiosis between CG and CF. The analysis identified several statistically significant taxa, including *C. albicans* (*p* < 0.03) and *Penicillium sp*. (*p* < 0.05), as illustrated in Figure 4, albeit with elevated *p*-values resulting from variance. A slight improvement in statistical confidence was observed with the multiple ITS1–ITS2–18S dataset via ALDEx2 LDA analysis, in which a balance was achieved between fungal resolution and broader eukaryotic context, resulting in moderate effect size differentiation and indicating heightened dataset complexity and within-group variability. The ITS1–18S and ITS2–18S combination exhibited a lesser array of species and reduced capacity for mycobiome community analysis (Supplementary Figure S10, 11) (Table 4).

**TABLE 4:** Comparison of combined primer sets in ALDEx2 group discrimination.

Criterion	*Triple combined*	*ITS1–18S*	*ITS2–18S*	*ITS1–ITS2*	*18S*	*ITS1*	*ITS2*
Taxa separation (effect size range)	Moderate	Narrow	Broader	Widest	Very narrow	Moderate	Moderate
High-confidence hits (adj. *p* < 0.05)	Moderate	Few	More	Multiple with clarity	Very limited	Moderate	Not detected
Distinct group enrichment (CF vs. CG)	Balanced	Skewed	Clear CG	Well-separated	Minimal, poorly defined	Partial, less distinct	Weak
Highlighted markers (e.g., *C. albicans*)	Present	Present	Strong	Very strong (CG)	Inconsistent detection	Detected, lower effect	Minimal signal
Undetected signals surfaced	Yes	Limited	Moderate	Yes, e.g., Penicillium	Rare signals surfaced	Some rare taxa surfaced	Poor detection

CF, cancer-free participants; CG, cancer group.

Overall, diversity patterns indicate that microbial community richness and evenness are generally greater in cancer-free individuals, especially when the 18S and ITS2 primers are used (Supplementary Figure S12). In addition, beta diversity was assessed using the principal coordinate analysis (PCoA) (Supplementary Figure S13); the ordination values exhibited distinct separation between the CF and CG groups across all three primer sets. These data affirm that microbial community composition varies greatly among groups, possibly attributable to underlying pathological features. However, there was no significant variation regarding the three primer sets individually. With the multiple combined datasets, the overall trend demonstrates the strongest diversity signals with the triply combined datasets, as shown in Supplementary Figure S14 and Supplementary Table S1.

Principal component analysis of the feasibility test CG group exhibited a greater variation in PCA plots (Supplementary Figures S15–18), indicating heterogeneous fungal dysbiosis. Compared to the corresponding CF group, tight clustering indicates stable commensal communities. The combination effect of ITS1–ITS2 enhanced the identification of infrequent species while preserving substantial differentiation (e.g., PC1 = 18%). Conversely, 18S primers introduced noise from non-fungal eukaryotes, reducing specificity. ALDEx2’s compositional method (CLR) surpassed DESeq2 (variance-stabilized transformation, VST) in detecting dysbiosis caused by relative abundance shifts, particularly for low-biomass taxa*.* The observed difference between the elevated PC1 variance in DESeq2 (29%–38%) and that in ALDEx2 (16%–22%) signifies fundamental methodological differences: DESeq2’s variation is influenced by predominant absolute abundances, including non-fungal eukaryotes in 18S-containing combinations, whereas ALDEx2’s lower yet more biologically meaningful percentages capture compositional alterations within fungal communities. This emphasizes that variance percentage alone is insufficient for evaluating separation quality; ALDEx2’s 19% for ITS1–ITS2 yields more informative biological insights than DESeq2’s 32% for the identical dataset (Table 5).

**TABLE 5:** Comparison of PCA results using ALDEx2 (CLR) vs. DESeq2 (VST) across primer set combinations.

Primer combination	ALDEx2 (CLR transformation)	DESeq2 (VST transformation)
ITS1*–*ITS2	Distinct group separation observed (PC1: 19% variance); effectively captures rare taxa and compositional shifts	Weaker group separation (PC1: 32.1%); primarily driven by dominant taxa
ITS1*–*18S	Partial separation achieved (PC1: 22%); reveals potential interactions between fungal and non-fungal eukaryotes	Minimal separation (PC1: 29.3%); likely influenced by broad eukaryotic background noise
ITS2*–*18S	Enhanced compositional resolution (PC1: 16%) despite 18S signal noise; better reflects fungal diversity	Moderate separation (PC1: 38.2%); clustering influenced predominantly by 18S-derived eukaryotic taxa
ITS1*–*ITS2*–*18S	Improved detection of relative abundance differences (PC1: 22%); compositional differences highlighted	Emphasizes absolute abundance differences (PC1: 29%); reduced sensitivity to compositional variation

PCA, principal component analysis. The CLR transformation of ALDEx2 illustrates the uniqueness of group differentiation and compositional insights derived from various primer combinations. DESeq2’s VST captures absolute abundance trends while reducing sensitivity to low-abundance taxa. PC1 denotes the initial principal component that encapsulates the maximum variation.

## 4 Discussion

This study represents the first systematic comparative assessment of multi-marker integration strategies for profiling the human gut mycobiome. The freshness of our approach stems from the thorough evaluation of 18S rRNA, ITS1, and ITS2 primers, individually and in strategic combinations, providing unprecedented insights into the synergistic impacts of multi-marker datasets on taxonomic resolution and biomarker detection sensitivity. Despite the limited sample size, our methodological framework demonstrates greater efficacy for integrated primer techniques than for single-marker strategies.

### 4.1 Impact of primer selection on fungal diversity

Our results indicate that primer selection markedly affects the richness, evenness, and taxonomic resolution of microbial communities. This finding supports previous research highlighting the primer selection effect on amplicon-based sequencing for the necessity of choosing suitable primers according to the study’s objectives, geographical location, and the precise characterization of complex fungal communities (Hoggard et al., 2018b).

### 4.2 Performance of ITS primers in OTU detection

The ITS1 and ITS2 primers consistently identified a greater number of OTUs than the 18S rRNA primer set (Figure 2c). This was particularly evident in their ability to capture higher richness and detect a greater number of group-specific OTUs unique to each cohort. These results align with prior studies demonstrating that the ITS region, particularly ITS2, offers enhanced taxonomic resolution for fungal communities (Banchi et al., 2020; Schoch et al., 2012b). The superior sensitivity and specificity of ITS primers render them more appropriate for fungal diversity research, whereas the more conserved 18S rRNA is less adept at differentiating closely related fungal species, a finding that underscores the necessity of sufficient sequencing depth in metagenomic investigations to prevent underestimating microbial diversity (Lundberg et al., 2012).

### 4.3 Fungal family patterns and ecological relevance

The significant diversity at the fungal family level observed with ITS primers across samples, such as Aspergillaceae*,* Saccharomycetaceae, and Pichiaceae*,* was among the most prevalent, indicating their ecological importance and opportunistic functions within the investigated environments. On the other hand, other families, such as *Herpotrichiellaceae* and *Trichomonadaceae* were found in low abundances, potentially indicating niche specialization or detection constraints attributable to primer bias (Lindahl et al., 2013).

### 4.4 Group-specific diversity

The variability among the CF and CG groups, particularly with ITS1 and ITS2, demonstrates higher microbial richness and evenness among the groups. This may indicate environmental variations in the health condition niche, affecting community diversity (Lozupone et al., 2012). In addition to the disparity in fungal composition between groups, as shown by PCoA and PCA grouping patterns, studies align with the fact that the integration of both ITS regions provides a holistic perspective on fungal populations (Mbareche et al., 2020a; Monard et al., 2013). Furthermore, fungal dysbiosis, marked by reduced richness and altered community structure, has been increasingly recognized as a hallmark of pathological conditions (Mukher et al., 2015) (Paterson et al., 2017b), including cancer (Paterson et al., 2017a) (Vallianou and Stratigou, 2021). The lower fungal diversity observed in cancer patients in our foundational study groups aligns with prior reports suggesting that the disruption of the normal mycobiome may contribute to disease status. In our study, dysbiosis was observed as an outcome of disease status; however, this observation needs validation in a larger cohort through comparison between cancer patients with confirmed illness (CG) and cancer-free controls. In particular, in the non-GI subgroup, there was a considerable increase of *Candida albicans*, an opportunistic fungus commonly observed in immunocompromised states. This discovery indicates that microbial imbalances may not only reflect the underlying disease context but could also actively facilitate disease progression by inducing immune dysregulation or mucosal susceptibility, thus facilitating worsening disease outcomes. These findings underscore the mycobiome’s potential role as both an indicator and a facilitator of disease status in cancer patients (Lozupone et al., 2012; Vallianou et al., 2021). For instance, our dual ITS1–ITS2 combination in the proof-of-concept cohort resulted in the greatest effect size separation and detection of multiple high-confidence fungal markers, particularly *C. albicans*, which increased in the cancer group, and *S. cerevisiae*, which was elevated in the cancer-free group.

This pattern is in line with multiple studies (Mukher et al., 2015; Rizzatti et al., 2017; Neville et al., 2015; Nenciarini et al., 2024), thus, our observation fits the expected trend that disease-associated dysbiosis often favors opportunistic fungi, while probiotic species dominate healthy microbiomes.

### 4.5 Discriminatory power of ITS in LDA analysis

Our LDA results emphasized the importance of robust statistical mycobiome frameworks, such as LEfSe, in identifying microbial signatures relevant to group differentiation. Moreover, Mbareche et al. (2020b) demonstrated in prior research that comparing ITS1 and ITS2 has yielded inconsistent results, suggesting that ITS1 may exceed ITS2 in delineating fungal diversity, particularly in specialized contexts such as bioaerosols, a setting characterized by mixed microbial populations (Lozupone et al., 2012). This evidence potentially corroborates our results regarding differential LDA, where ITS1 produced more differential taxa than ITS2. The gut microbiome constitutes a complicated environment, and this finding indicates the potential of ITS1 to better understand fungal diversity within complex communities regarding LDA. In our study, ITS1 similarly yielded a marginally broader set of differentially abundant taxa, as reflected in the LDA results, suggesting its utility for distinguishing fungal profiles even in diverse host-associated environments.

### 4.6 Broader eukaryotes or fungal specificity

The inclusion of 18S rRNA expanded eukaryotic detection, revealing broader community structures, including species such as *K. humilis*, *Pichia spp*., and other eukaryotes. This, however, resulted in diminished fungal specificity and heightened dataset complexity, aligning with established limits of 18S in mycobiome research (Tonge et al., 2014). These trade-offs were evident in PCA and differential abundance analysis, where 18S datasets included non-fungal interference and reduced statistical clarity. Notwithstanding these constraints, the fully integrated dataset (ITS1–ITS2–18S) yielded the most comprehensive ecological profile, encompassing both predominant fungal species (*C. albicans*, *C. parapsilosis*, and *S. cerevisiae*) and broader unclassified eukaryotic signals. Nevertheless, as noted in recent multi-marker investigations (Bokulich and Mills, 2013; D’Andreano et al., 2021), augmented taxonomic coverage resulted in intra-group heterogeneity, necessitating more conservative statistical thresholds.

### 4.7 ALDEx2 vs. DESeq2 for differential abundance

As mentioned above, C. albi*cans* exhibited a substantial LDA score in CG using the aggregated dataset; nevertheless, its statistical significance diminished (*p*-adj = 0.08) relative to studies utilizing individual ITS primers. This illustrates the importance of analyzing both biological impact sizes and adjusted *p*-values in multi-marker research.

The ALDEx2 analysis of pairwise integrated ITS1–ITS2 datasets decreases primer-specific biases and, as previously mentioned, improves the discovery of both dominant taxa (*Candida sp*.) and low-abundant taxa (*Penicillium* sp.*)* that might be implicated in cancer-associated dysbiosis. The concordance between ALDEx2 impact sizes and LDA results reinforces the credibility of the identified microbial signatures, consistent with evidence that ITS-based primers outperform 18S for species-level fungal resolution (Tedersoo et al., 2015; Tonge et al., 2014; Zhao et al., 2017). Furthermore, the CLR-based PCA from ALDEx2 results outperformed DESeq2’s variance-stabilizing transformation in distinguishing mycobiome communities across different clinical groups by analyzing the compositional characteristics of microbiome data and minimizing the effect of dominant taxa. Conversely, DESeq2’s VST clustered samples by absolute abundance, potentially masking relevant taxon composition shifts in community structure.

Our findings further highlight that ITS1 or ITS2 alone is only partially informative for thorough gut mycobiome characterization. Single-marker approaches exhibited insufficient resolution and sensitivity to reliably identify disease-associated species such as *C. parapsilosis* and *Penicillium spp*., corroborating previous findings that primer biases constrain fungal community evaluations (Abid et al., 2022; Bellemain et al., 2010).

## 5 Strengths and limitations

This study was conducted through the thorough evaluation of three commonly utilized fungal DNA regions—18S rRNA, ITS1, and ITS2—across various analytical dimensions, including OTU richness, taxonomic resolution, alpha and beta diversity, and biomarker identification. The comprehensive analytical framework, which integrates bioinformatics tools, including USEARCH, SILVA, and UNITE databases, LEfSe, and PCA/PCoA, ensures considerable reliability and reproducibility in community profiling. Additionally, testing fecal samples incorporating from both cancer group (CG) and cancer-free (CF) subjects facilitated disease-specific microbiological analysis and the identification of group-specific biomarkers. The limitation of this study is the sample size; the utilization of only eight samples may constrain statistical power and the generalizability of the findings to broader populations.

Furthermore, despite the utilization of rarefaction curves, certain samples (e.g., G41) exhibited inadequate richness and non-saturation, indicating that the sequencing depth might be insufficient to fully capture community complexity in specific instances. This suggests that factors influencing mycobiome profiling do not rely solely on primer selection; other factors extend beyond primer selection, including sample quality, host condition, fungal load, or geographical variations.

## 6 Conclusion

In conclusion, this proof-of-principle study establishes the methodological basis for multi-marker mycobiome profiling, revealing for the first time the synergistic advantages of mixing 18S rRNA, ITS1, and ITS2 primer datasets.

In our study, ITS1 and ITS2 demonstrated complementary strengths. ITS1 tended to increase the differentially abundant taxa, reflecting higher richness, whereas ITS2 captured a greater number of OTUs; both facilitate a more thorough evaluation of fungal diversity, encompassing a broader spectrum of taxa and ecological variation. The pairwise integration of ITS–ITS2 datasets provided more discriminatory power, uncovering dysbiosis predictors that might be overlooked with single-primer methodologies. On the other hand, the triple integration of ITS1–ITS2–18S offered greater richness, demonstrating a comprehensive profiling of the fungal community.

Studies employing amplicon sequencing should focus on creating taxonomically balanced, multi-locus primer panels for mycobiome assessment. This technique tackles existing limitations in primer bias and facilitates comprehensive characterization of fungal dysbiosis in health–disease transitions. ALDEx2 presented an in-depth structure for differential abundance analysis by enhancing the discovery of both prevalent and low-abundance taxa likely associated with cancer-related dysbiosis, improving group differentiation, and minimizing distortion from dominant taxa. These findings highlight the importance of ALDEx2 in multi-marker mycobiome research in small cohort contexts when statistical power is constrained. In summary, multi-marker integration boosts the detection of both common and rare taxa, reduces primer-specific biases, and improves the overall interpretability of gut fungal communities. The heightened complexity of datasets requires meticulous statistical analysis, especially in low-biomass settings such as the human gut.

## Data Availability

The raw data supporting the conclusions of this article will be made available by the authors, without undue reservation.
