# Supplementary material for: Multi-marker comparative analysis of 18S, ITS1, and ITS2 primers for human gut mycobiome profiling
Source: Front Bioinform. 2025 Nov 19;5:1690766. doi: 10.3389/fbinf.2025.1690766 (PMC12672528; doi:10.3389/fbinf.2025.1690766)
Supplement: Supplementary file 2 [file Table2.docx]

**Step-by-Step Bioinformatics Protocol (Supplementary Material)**

**Title:**

Step-by-Step Bioinformatics Analysis Protocol for “a comparative analysis of 18S, ITS1, and ITS2 primers for mycobiome profiling in human”

**Introduction:**

This supplementary protocol presents comprehensive bioinformatics analyses, multi-datasets of amplicon sequencing for gut mycobiome profiling: ITS1, ITS2, and 18S as an integration strategy conducted in the primary manuscript, labelled " comparative analysis of 18S, ITS1, and ITS2 primers for mycobiome profiling in human". The protocol is designed to ensure the reproducibility of the results obtained. All program versions, parameters, and commands utilized are clearly stated.

**Objective:**

To enhance fungal taxonomic resolution and detection efficacy by combining datasets from ITS1, ITS2, and 18S amplicons in faecal samples using standardized pipelines. This protocol enables comprehensive mycobiome profiling while accounting for compositionality and marker biases.

**Validation of the Method:**

- **Databases Used:** SILVA (for 18S) and UNITE (for ITS regions).
- **Toolkits:** DADA2, R packages includes phyloseq, ALDEx2, DESeq2.
- **Cross-validation:** Compared single-amplicon vs. combined datasets on taxa richness, PCA resolution, LDA effect size, and adjusted p-values.
- **Effectiveness:** Enhanced detection of fungal dysbiosis markers

**Materials and Software:**

- **Hardware Requirements:**

CPU

RAM 16G

Disk Space

- **Operating System:**

Windows 11 Enterprise 22H2

- **Platforms**

R 4.4.0

Python 3.12.3

- **Toolkits:**

| - **R tools:** |  | **Python tools:** |
| --- | --- | --- |
| DADA2 |  | Pandas |
| BiocManager |  | NumPy |
| Blocstyle |  | Seaborn |
| ggplot2 |  | Matplotlib |
| MiaV |  |  |
| Vegan |  |  |
| ALDEx2 |  |  |
| DESeq2 |  |  |
| metagenomeSeq |  |  |
|  |  |  |

- **Software:**

Bioconductor 3.20

- **Databases:**

SILVA 138.1

UNITE v.8.2

**Step-by-Step Protocol**

**Method:**

3.1 Data Acquisition:

The data provided in the FASTA files and the OTUs table and taxonomy tables were obtained from a sequencing company that was outsourced out (BGI Genomics ltd).

**STEP 1: rarefaction curve for the expected OTUs richness**

Species accumulation curves were created using R 4.4.0 to illustrate the elevation in detected species with the inclusion of each sample. The *microbiome* utility tools in R were utilized to understand the composition of the community and predict the richness of species, which is frequently employed to evaluate the adequacy of sampling volume and species richness in biodiversity assessments. The rarefaction curve aids in assessing whether the sequenced data adequately represents all possible species, with the curve often levelling off as sampling nears saturation.

#import datasets (out_tables) individually:

# Convert OTU table to a matrix

otu_tab <- as(otu_table(physeq), "matrix")

# Remove samples with zero counts

otu_tab <- otu_tab[rowSums(otu_tab) > 0, ]

# Define colors based on the number of rows (samples) in the filtered OTU table

colors <- rainbow(nrow(otu_tab))

# Plot rarefaction curve

p <- vegan::rarecurve(

otu_tab,

step = 50,

label = TRUE,

col = colors,

cex = 0.8,

ylab = "Expected OTU Richness",

lwd = 2

)

**STEP 2: Dataset Merging**

- **Merge OTU and taxonomy tables** for combinations:
  - ITS1-ITS2
  - ITS1-18S
  - ITS2-18S
  - ITS1-ITS2-18S
- **Tools:** phyloseq + microbiome R package
- **Transformation:** Centered log-ratio (CLR) for ALDEx2

#importing dataset’s files path:

otu_its1 <- read.csv("C:\\Users\\hibaorsud\\Desktop\\3primers-data\\re-analysis\\otu_table_ITS1.csv", row.names = 1)

otu_18s <- read.csv("C:\\Users\\hibaorsud\\Desktop\\3primers-data\\re-analysis\\otu_table_18s.csv", row.names = 1)

#otu_its2 <- read.csv("C:\\Users\\hibaorsud\\Desktop\\3primers-data\\re-analysis\\otu_table_ITS2 -new.csv", row.names = 1)

tax_its1 <- read.csv("C:\\Users\\hibaorsud\\Desktop\\3primers-data\\re-analysis\\taxa_table_ITS1 - species.csv", row.names = 1)

tax_18s <- read.csv("C:\\Users\\hibaorsud\\Desktop\\3primers-data\\re-analysis\\taxa_table_18s - species.csv", row.names = 1)

#tax_its2 <- read.csv("C:\\Users\\hibaorsud\\Desktop\\3primers-data\\re-analysis\\taxa_table_ITS2 -species.csv", row.names = 1)

#Step-by-Step: Merging and Preparing for ALDEx2

#1. Make OTU IDs unique across datasets

rownames(otu_its1) <- paste0("ITS1_", rownames(otu_its1))

rownames(otu_18s) <- paste0("ITS1_", rownames(otu_18s))

#rownames(otu_its2) <- paste0("ITS2_", rownames(otu_its2))

rownames(tax_its1) <- rownames(otu_its1)

rownames(tax_18s) <- rownames(otu_18s)

#rownames(tax_its2) <- rownames(otu_its2)

##For the three ;;; Add row names as a column:

#Before merging, convert row names into an explicit column (e.g., "OTU"), then merge:

otu_its1$OTU <- rownames(otu_its1)

otu_18s$OTU <- rownames(otu_18s)

#otu_its2$OTU <- rownames(otu_its2)

### 3. Use Reduce + merge:

#This helps combine all three using OTU as the common column:

otu_combined <- Reduce(function(x, y) merge(x, y, by = "OTU", all = TRUE),

list(otu_its1, otu_18s, otu_its2))

###Set OTU back as row names

rownames(otu_combined) <- otu_combined$OTU

otu_combined$OTU <- NULL

#2. Combine OTU tables (fill missing with 0s)

otu_combined <- merge(otu_its1, otu_18s, by = "row.names", all = TRUE)

rownames(otu_combined) <- otu_combined$Row.names

otu_combined$Row.names <- NULL

otu_combined[is.na(otu_combined)] <- 0

#3. Combine taxonomy tables

tax_combined <- rbind(tax_its1,tax_18s)

tax_combined[is.na(tax_combined)] <- "unclassified"

#4. Filter at Species Level (or Genus if Species missing)

library(dplyr)

# Keep only species-level assignments

tax_combined_species <- tax_combined %>%

filter(Species != "unclassified") %>%

select(Species)

# Collapse OTU table by species

#otu_combined$Species <- tax_combined_species[rownames(otu_combined), "Species"]

# Add Species column (as before)

otu_combined$Species <- tax_combined_species[rownames(otu_combined), "Species"]

# How many missing?

sum(is.na(otu_combined$Species))

otu_combined <- otu_combined[!is.na(otu_combined$Species), ]

library(dplyr)

library(tibble)

otu_by_species <- otu_combined %>%

group_by(Species) %>%

summarise(across(where(is.numeric), sum, na.rm = TRUE)) %>%

column_to_rownames("Species")

#5. Prepare ALDEx2 Inputs

library(ALDEx2)

# Transpose for ALDEx2 (samples as rows)

otu_for_aldex <- as.data.frame(t(otu_by_species))

........................

ncol(otu_for_aldex)

length(conds)

group <- c(0, 0, 0, 0, 1, 1, 1, 1) # From your table

conds <- ifelse(group == 0, "CF", "CG")

conds

# Create condition vector

conds <- read.csv("C:\\Users\\hibaorsud\\Desktop\\3primers-data\\re-analysis\\8_sample_data.csv", row.names = 1)

#conds <- c("CF", "CF", "CF", "CF", "CG", "CG","CG", "CG") # update based on your metadata

colnames(otu_for_aldex)

otu_for_aldex <- as.data.frame(otu_by_species)

head(otu_for_aldex)[, 1:5]

# Collapse duplicate sample columns, or select just one source (e.g., ITS2)

otu_for_aldex <- otu_combined[, unique(colnames(otu_combined))]

............................

# Match the correct sample order

sample_names <- colnames(otu_for_aldex)

# Assign conditions manually (must match `sample_names` order exactly)

conds <- c("CF", "CF", "CF", "CF", "CG", "CG", "CG","CG", "CF", "CF", "CF", "CF", "CG", "CG", "CG", "CG")

# Run ALDEx2

aldex_clr <- aldex.clr(otu_for_aldex, conds, mc.samples = 128, denom = "all", verbose = FALSE)

aldex_result <- aldex.ttest(aldex_clr)

aldex_effect <- aldex.effect(aldex_clr)

# Combine results

aldex_output <- cbind(aldex_result, aldex_effect)

head(aldex_output)

#Add significance flag

aldex_output$significant <- ifelse(aldex_output$we.ep < 0.05, "*", "")

**Troubleshooting:**

**Integration and Merging Challenges of OTU Tables:**
During the integration of OTU tables from several amplicon datasets (ITS1, ITS2, and 18S), multiple problems arose due to discrepancies in OTU identifies and matrix dimensions. In particular:

- The OTU identifiers differed among datasets, even if they represented identical or overlapping taxa.
- The taxonomy columns exhibited different formats and naming conventions
- Inconsistencies in row lengths and absent entries hindered automatic merging with conventional tools in R (e.g., merge_phyloseq, phyloseq::merge_taxa, or bespoke dplyr scripts).
- The inconsistencies resulted in dimension mismatch errors and subscript out-of-bounds exceptions during the alignment of abundance matrices or the programmatic assignment of taxonomy names.

**Manual Resolution**
In light of persistent automated merging failures, we addressed the issue manually by:
Exporting OTU tables and taxonomy assignments from each dataset to CSV format.
Manually synchronizing OTU identifiers and taxonomic labels between datasets via Microsoft Excel. Generating integrated OTU abundance matrices and their associated taxonomy tables through:
Aligning OTUs based on taxonomic resemblance, and imputing non-existent OTUs with zeros in the dataset. Maintaining uniform formatting for columns and rows.
The manually curated combined datasets were subsequently re-imported into R for subsequent compositional analysis.

**STEP 3: OTUs distribution**

The distribution of OTUs was analysed using Python 3.12.3, utilizing the *venn3* package to generate Venn diagrams. The allocation of OTUs among different groups was demonstrated using bar charts created with *Matplotlib* and *Seaborn*. This approach enables a precise comparison of shared and unique taxa across many categories.

**Tool: Python libraries**

import pandas as pd

import numpy as np

import matplotlib as plt

import matplotlib.pyplot as plt

import seaborn as sns

from google.colab import files

uploaded = files.upload()

com = pd.read_csv("otu_table_combined.csv")

com

# Create a new DataFrame with the desired columns and calculate the sum for each sample

sample_sums = com[['A4', 'A21', 'A25', 'A27', 'B20', 'G41', 'G48', 'G50', 'PrimerSet']].groupby('PrimerSet').count()

# Transpose the DataFrame to have samples as rows and PrimerSet as columns

sample_sums_transposed = sample_sums.transpose()

# Transpose the DataFrame to have samples as rows and PrimerSet as columns

sample_sums_transposed = sample_sums.transpose()

# Create the bar chart

sample_sums_transposed.plot(kind='bar')

plt.title('number of OTUs in in Each Sample')

plt.xlabel('Samples')

plt.ylabel('sum of OTUs')

plt.legend(bbox_to_anchor=(1.05, 1), loc='upper left')

plt.tight_layout()

plt.savefig('otu_bar_chart.tif')

# Download the figure

files.download('otu_bar_chart.tif')

plt.show()

# prompt: plot 3primer sets frequency

import matplotlib.pyplot as plt

# Assuming 'prim' DataFrame is already loaded as in the previous code

primer_counts = prim['PrimerSet'].value_counts()

plt.figure(figsize=(10, 6))

sns.histplot(data=prim, x='PrimerSet', hue='OUT', multiple='stack', palette=['skyblue', 'lightcoral', 'gold'], legend =False)

plt.xticks(prim['PrimerSet'].unique(), ['18S', 'ITS1', 'ITS2'])

plt.xlabel('Primer Set')

plt.ylabel('Frequency')

plt.title('Frequency of OTUs')

plt.xticks(rotation=45, ha='right')

plt.tight_layout()

# Download the figure

plt.savefig('otu_bar_primersets.tif')

files.download('otu_bar_primersets.tif')

plt.show()

**STEP 4: phyloseq construction:**

**Tool: phyloseq package**

#importing merge files

otu_table <- read.csv("C:\\Users\\hibaorsud\\Desktop\\3primers-data\\re-analysis\\Combined\\3-otu_table_combined.csv", row.names = 1)

otu_table <- otu_table(otu_table, taxa_are_rows = TRUE)

tax_table <- read.csv("C:\\Users\\hibaorsud\\Desktop\\3primers-data\\re-analysis\\Combined\\3-otu_table_combined.csv", row.names = 1)

tax_table <- as.matrix(tax_table)

sample_data <- read.csv("C:\\Users\\hibaorsud\\Desktop\\3primers-data\\re-analysis\\8_sample_data.csv", row.names = 1)

sample_data <- sample_data(sample_data)

# Step 2: Convert the data frame to a matrix

tax_matrix <- as.matrix(tax_table)

# Step 3: Create a tax_table object

tax_table <- tax_table(tax_matrix)

# Check the tax_table

print(tax_table)

# Convert OTU table and taxonomy table to appropriate formats

otu_table <- otu_table(as.matrix(otu_table), taxa_are_rows = TRUE)

tax_matrix <- as.matrix(tax_table)

tax_table <- tax_table(tax_matrix)

# Check if row names match

otu_taxa_names <- rownames(otu_table)

tax_taxa_names <- rownames(tax_table)

if (!all(otu_taxa_names %in% tax_taxa_names) || !all(tax_taxa_names %in% otu_taxa_names)) {

stop("OTU names and taxonomy names do not match")

}

print(otu_taxa_names)

print(tax_taxa_names)

# Ensure sample data is formatted correctly

sample_data <- sample_data(sample_data)

# Create phyloseq object

#physeq <- phyloseq(otu_table, tax_table, sample_data, treeNJ)

physeq <- phyloseq(otu_table, tax_table, sample_data)

print(physeq)

**STEP 5: Taxonomic Filtering and Grouping**

**Tool: phyloseq**

# Define the standard ranks

standard_ranks <- c("Species")

# Filter the taxonomy table to include only the standard ranks

filtered_tax_table <- tax_table[, standard_ranks, drop = FALSE]

# Replace the taxonomy table in the phyloseq object

tax_table_ps<- filtered_tax_table

tax_table_ps

ps <- phyloseq(otu_table, tax_table_ps, sample_data)

print(ps)

sample_data(ps)$group <- factor(sample_data(ps)$group,

levels = c(0, 1),

labels = c("CF", "CG"))

# Check the tax_table

print(tax_table(ps))

# Check taxa ranks

print(rank_names(ps))

# Remove taxa with no classification

ps <- prune_taxa(taxa_sums(ps) > 0, ps)

# Detach the tidytree package if it's not needed

detach("package:tidytree", unload = TRUE)

# Get OTU table and sample metadata

otu <- as.data.frame(phyloseq::otu_table(ps))

group <- phyloseq::sample_data(ps)$group

# Filter out taxa found in only one group

keep_taxa <- apply(otu, 1, function(x) {

sum(tapply(x, group, function(g) sum(g > 0)) > 0) > 1

})

ps_filtered <- phyloseq::prune_taxa(keep_taxa, ps)

**STEP 6: differential heatmap for combined datasets:**

print(physeq)

##For top 20 species:

# Check the taxonomic table

tax_table(physeq)

# Select the top 20 species based on mean relative abundance

ps1.com.fam <- microbiome::aggregate_taxa(physeq,"Species")

ps1.com.fam.rel <- microbiome::transform(ps1.com.fam, "compositional")

top20_species <- names(sort(taxa_sums(ps1.com.fam.rel), decreasing = TRUE)[1:10])

ps1.com.fam.rel.top10 <- prune_taxa(top20_species, ps1.com.fam.rel)

# Plot the composition and set the x-axis label

plot.composition.relAbun <- plot_composition(ps1.com.fam.rel.top10,

x.label = "group")

# Extract the plotting data

data.com <- plot.composition.relAbun$data

# Print the filtered data to verify

print(head(data.com))

# Filter to keep only species level data in the Tax column

#data.com <- data.com[data.com$Tax == "Species", ]

# Now you can use data.com for further analysis or plotting

=======================================

# Print the filtered data to verify

print(head(data.com))

colnames(data.com)

data.com$xlabel <- factor(data.com$xlabel,

levels = c(0, 1),

labels = c("CF", "CG"))

# base plot

p.heat <- ggplot(data.com, aes(x = Sample, y = Tax)) + geom_tile(aes(fill = Abundance))

# Change color

p.heat <- p.heat + scale_fill_distiller("Abundance", palette = "RdYlBu") + theme_bw()

# Make bacterial names italics

p.heat <- p.heat +

theme(axis.text.y = element_text(colour = 'black', size = 10, face = 'italic'))

p.heat <- p.heat +

theme(

axis.ticks.y = element_line(linewidth = 0.1))

# Make seperate samples based on main varaible

p.heat <- p.heat + facet_grid(~xlabel,

scales = "free") + rremove("x.text")

p.heat <- p.heat + ylab("Species")

#Clean the x-axis

p.heat <- p.heat +

theme(

axis.title.x = element_blank(), # Remove x-axis title

axis.text.x = element_text(angle = 90, size = 7, color = "black", hjust = 1, vjust = 0.5),

axis.ticks.x = element_blank() # Remove x-axis ticks

)

# Clean the facet label box

p.heat <- p.heat + theme(legend.key = element_blank(),

strip.background = element_rect(colour="black", fill="white"))

print(p.heat)

**STEP 7: Taxa prevalence and diversity analysis for Separate datasets**

**7.1 Prevalence Rates**

The overall taxonomy prevalence was measured using the *MiaViz* and *Phyloseq* R packages, a method that provides a comprehensive overview of microbial prevalence across samples, which in which transform microbiome count data into average count log scale and percentage scale, enabling the identification of specific taxa abundance across diverse ecosystems.

**7.2 Diversity analysis**

Alpha and beta diversity were conducted utilizing the *vegan* package in R.

7.2.1 Alpha diversity denotes the species diversity inside an individual sample and is measured using various indices of richness and evenness: Observed Species richness, Chao, Shannon and Inverse Simpson reflecting how different taxa are richly and evenly distributed within a sample.

7.2.2 Beta diversity refers to the species diversity across groups, and it was evaluated to determine the variations in microbial community composition among samples with unweighted UniFrac distances for sample dissimilarity measurement in form of PCoA non-linear dimension reduction technique. It quantifies differences in the overall taxonomic composition between two samples in different groups. This procedure facilitates the comparison of microbial community structures across samples or groups.

**Tool: vegan**

#construct phyloseq separately:

# Transform data to relative abundance

physeq_relabund <- transform_sample_counts(physeq, function(x) x / sum(x))

# Check the transformed phyloseq object

print(physeq_relabund)

# Aggregate data at the Phylum/family/species level

physeq_Phylum <- tax_glom(physeq_relabund, "Phylum")

# Convert to a data frame for ggplot2

physeq_df <- psmelt(physeq_Phylum)

# Print the plot

pg <- ggplot(physeq_df, aes(x = Sample, y = Abundance, fill = Phylum)) +

geom_bar(stat = "identity", position = "stack") +

theme_minimal() +

theme(legend.key.height = unit(0.15, "cm"),

axis.text.x = element_text(angle = 90, vjust = 0.5, hjust=1, size = 10),

axis.title.x = element_text(size = 12, face = "bold"),

axis.title.y = element_text(size = 12, face = "bold")) +

scale_y_continuous(labels = scales::percent) +

labs(title = "Taxa Prevalence at phylum Level", x = "Samples", y = "Relative Abundance")

print(pg)

#Alpha diversity:

# quick check taxa prevalence

p.rar <- plot_taxa_prevalence(physeq, "Family")

p.rar

hmp.div <- alpha(physeq, index = "all")

hmp.meta <- meta(physeq)

# rownames added as a new colum for easy integration later.

hmp.meta$sam_name <- rownames(hmp.meta)

#rownames added to diversity table

hmp.div$sam_name <- rownames(hmp.div)

# these two data frames were merged into one

div.df <- merge(hmp.div,hmp.meta, by = "sam_name")

colnames(div.df)

# conditions were converted to factor and set the labels

div.df$Treatment.type <- factor(div.df$group,

levels = c(0, 1),

labels = c("CF", "CG"))

# the div.df data frame Converted to long format

div_df_long <- div.df %>%

pivot_longer(

cols = -c(sam_name, group),

names_to = "diversity_measure",

values_to = "value"

)

head(hmp.meta)

# By Meling index

# phyloseq object converted into a long data format.

c("group", "observed", "chao1", "diversity_inverse_simpson", "diversity_gini_simpson")]

colnames(div.df)

div.df2 <- div.df[, c("Event.status", "observed", "chao1", "diversity_inverse_simpson", "diversity_gini_simpson")]

colnames(div.df2) <- c("group", "observed", "chao1", "diversity_inverse_simpson", "diversity_gini_simpson")

div_df_melt <- reshape2::melt(div.df2, c("group"))

head(div_df_melt)

# div_df_melt used as data frame to plot

p <- ggboxplot(div_df_melt, x = "Cancer.types", y = "value",

fill = "group",

palette = "jco",

legend= "right",

facet.by = "variable",

scales = "free")+

labs(title = "Comparison of Diversity Indices by Cancer.types",

x = "Cancer.types", y = "Diversity Index") +

scale_fill_manual(values = c("CF" = "skyblue", "CG" = "orange"))

#Beta diversity:

wt.unifrac <- plot_ordination(ps1.rel,

ordu.wt.uni, color="group")

#with significance-ordination-weighted

library(ggsignif)

# Generate the ordination plot object

ordu_plot <- plot_ordination(ps1.rel, ordu.wt.uni, color = "group")

# Extract the scores and metadata from the ordination plot object

plot_data <- ordu_plot$data

# Perform a significance test (e.g., Wilcoxon test)

test_result <- wilcox.test(Axis.1 ~ group, data = plot_data)

# Create the box plot with significance bars

p_box <- ggplot(plot_data, aes(x = group, y = Axis.1, fill = Treatment.type)) +

geom_boxplot() +

geom_jitter(width = 0.2, size = 1.5, alpha = 0.7, aes(group = Treatment.type)) +

theme_gray() +

scale_fill_manual(values = c("CF" = "steelblue", "CG = "tomato")) +

labs(title = "Box Plot of PCoA1 by Treatment.type", x = "Treatment.type", y = "weighted unifrac") +

geom_signif(comparisons = list(c("with Immunotherapy", "without Immunotherapy")),

annotations = sprintf("p = %.2g", test_result$p.value),

y_position = max(plot_data$Axis.1, na.rm = TRUE) * 1.1, # adjust as needed

tip_length = 0.03)

# Print the plot

print(p_box)

**STEP 8: LEfSe Differential Abundance Analysis**

Linear Discriminant Analysis Effect Size (LEfSe) was conducted to find biomarkers that distinguish microbial communities among groups. LEfSe combines statistical significance and biological consistency to identify discriminative traits, which are ordered according to their effect magnitude. LEfSe analysis was conducted via the *microbiomeMarker* package in R, which applies the LEfSe methodology specifically designed for microbiome data. This investigation offered a comprehensive array of indicators that may aid in differentiating various groups based on microbial community composition.

**Tool: microbiomeMarker**

library(microbiomeMarker)

result <- run_lefse(

ps,

"group",

subgroup = NULL,

taxa_rank = "Species",

transform = c("identity", "log10", "log10p"),

norm = "none",

norm_para = list(),

kw_cutoff = 0.5, # Less stringent

lda_cutoff = 2, # Less stringent

bootstrap_n = 30,

bootstrap_fraction = 2/3,

wilcoxon_cutoff = 0.05, # Less stringent

multigrp_strat = FALSE,

strict = c("0", "1", "2"),

sample_min = 230,

only_same_subgrp = FALSE,

curv = FALSE

)

..........................

#lda abundance bar

ab <- plot_abundance(result,

label_level = 1,

max_label_len = 60,

markers = NULL,

group = "group")

ab

ab + scale_x_log10()

..............................

markers <- result@marker_table

if (is.null(markers) || nrow(markers) == 0) {

stop("No markers found in the result object.")

}

>>>>

plot_heatmap(result, group = "group")

>>>>>

# Extract and label significant markers

marker_df <- as.data.frame(result@marker_table)

markers$display_label <- ifelse(markers$padj < 0.05,

paste0(markers$feature, "*"),

markers$feature)

# Update feature names in the object itself

result@marker_table$feature <- marker_df$display_label

--------------

colnames(result@marker_table)

markers <- result@marker_table

markers$display_label <- ifelse(

markers$padj < 0.5,

paste0(rownames(markers), "*"),

rownames(markers)

)

result@marker_table$display_label <- markers$display_label

# Now plot using display_label values

plot_ef_bar(result,

label_level = 1,

max_label_len = 60,

markers = marker_df$display_label) +

ggtitle("LEfSe Analysis: Significant Markers (*)") +

theme(

plot.title = element_text(face = "bold", size = 14, hjust = 0.5),

legend.title = element_text(face = "bold", size = 10),

legend.text = element_text(size = 8),

legend.position = "right",

axis.text.x = element_text(angle = 0, hjust = 1)

) +

labs(fill = "group") +

scale_fill_manual(values = c(

"CF" = "#66C2A5",

"CG" = "#FC8D62"

))

**STEP 9: ALDEx2 LDA**

This method is particularly appropriate for microbiome data because of its compositional characteristics and its consideration of both biological and sampling variability. For each primer-specific count table (ITS1, ITS2, 18S, and their combinations), we conducted centred log-ratio (CLR) transformation, followed by ALDEx2's Welch's t-test and Wilcoxon Rank Sum test (aldex.ttest), to identify differentially abundant taxa between experimental groups (cancer vs. cancer-free). The Benjamini-Hochberg adjustment was employed to regulate the false discovery rate (FDR). ALDEx2 employs a Dirichlet-multinomial distribution to represent the data, rendering it particularly robust when handling sparse datasets and limited sample sizes. Linear Discriminant Analysis (LDA) effect size (LEfSe-style) was employed to illustrate the magnitude of effects of differentially frequent characteristics across several primer sets, demonstrating primer-dependent sensitivity in taxonomy identification.

**Tool: ALDEx2**

# Run ALDEx2 LDA volcano;;;

#group <- c(0, 0, 0, 0, 1, 1, 1, 1) # From your table

group <- c(0, 0, 0, 0, 1, 1, 1)

conds <- ifelse(group == 0, "CF", "CG")

conds

#aldex_._clr <- aldex.clr(otu_for_aldex, conds, mc.samples = 128, denom = "all", verbose = FALSE)

aldex_result <- aldex.ttest(aldex.clr)

aldex_effect <- aldex.effect(aldex.clr)

# Combine results

aldex_output <- cbind(aldex_result, aldex_effect)

head(aldex_output)

#Add significance flag

aldex_output$significant <- ifelse(aldex_output$we.ep < 0.05, "*", "")

library(ggplot2)

# Volcano plot for ALDEx2

# Check structure

head(tax_table)

head(aldex_output$Taxon)

# Map species names into aldex_output

aldex_output$Taxon <- tax_table[rownames(aldex_output), "Species"]

# Add labels for significant taxa only

aldex_output$label <- ifelse(aldex_output$we.ep < 0.05, aldex_output$Taxon, "")

# Plot with species names

ggplot(aldex_output, aes(x = effect, y = -log10(we.ep))) +

geom_point(aes(color = enriched_group), size = 2, alpha = 0.8) +

geom_text(data = subset(aldex_output, we.ep < 0.05),

aes(label = Taxon),

hjust = 0, vjust = 0.5, size = 3, check_overlap = TRUE) +

scale_color_manual(values = c("CF" = "forestgreen", "CG" = "red")) +

theme_minimal() +

labs(

title = "ALDEx2 Volcano Plot by Group Enrichment - ITS1",

x = "Effect Size (Difference Between Groups)",

y = "-log10 Adjusted p-value (Welch's test)",

color = "Enriched Group",

caption = "Labeled taxa have adjusted p-value < 0.05"

)

head(aldex_output)

aldex_output <- aldex_output %>% rownames_to_column("Taxon")

# Sort by significance (lowest we.ep)

aldex_output$Taxa <- your_taxonomic_names

top_sig <- aldex_output[aldex_output$we.ep < 0.05, ]

top_sig <- top_sig[order(top_sig$we.ep), ][1:10, ] # Top 10 by significance

#top_sig <- aldex_output[order(aldex_output$we.ep), ][1:10, ]

# Plot with only top 10 significant taxa labeled

ggplot(aldex_output, aes(x = effect, y = -log10(we.ep))) +

geom_point(aes(color = enriched_group), size = 2, alpha = 0.8) +

geom_text(data = top_labels, aes(label = Taxa),

hjust = 0, vjust = 0.5, size = 3, check_overlap = TRUE) +

scale_color_manual(values = c("CF" = "forestgreen", "CG" = "red")) +

theme_minimal() +

labs(

title = "ALDEx2 Volcano Plot by Group Enrichment - combined ITS1 & 18S",

x = "Effect Size (Difference Between Groups)",

y = "-log10 Adjusted p-value (Welch's test)",

color = "Enriched Group",

caption = "Top 10 taxa with lowest adjusted p-values"

)

top_labels <- aldex_output %>%

filter(we.ep < 0.05) %>%

arrange(we.ep) %>%

slice_head(n = 10)

ggplot(aldex_output, aes(x = effect, y = -log10(we.ep))) +

geom_point(aes(color = enriched_group), size = 2, alpha = 0.8) +

geom_text(data = top_labels, aes(label = Taxa),

hjust = 0, vjust = 0.5, size = 3, check_overlap = TRUE) +

scale_color_manual(values = c("CF" = "forestgreen", "CG" = "red")) +

theme_minimal() +

labs(

title = "ALDEx2 Volcano Plot by Group Enrichment - combined ITS1,ITS2 & 18S",

x = "Effect Size (Difference Between Groups)",

y = "-log10 Adjusted p-value (Welch's test)",

color = "Enriched Group",

caption = "Top 10 taxa with lowest adjusted p-values"

)

**STEP 10: DESeq2 PCA Analysis**

Principal Component Analysis (PCA) was performed to diminish data dimensionality and to find variation patterns among distinct groupings. PCA *DEseq* calculates the principal components (PCs) and reconfigures the data into a new coordinate system, wherein the initial two components often encapsulate the most important features of the dataset. The closeness of samples on the PCA plot signifies their similarity in microbial composition, whereas the differentiation of samples across various treatments or settings illustrates unique microbial profiles. This approach facilitates the visualization of groupings or clusters predicated on microbial diversity.

**Tool: DESeq2**

##DESeq PCA;;;;;;;;;;;

library(DESeq2)

library(ggplot2)

count_data <- read.csv("C:\\Users\\hibaorsud\\Desktop\\3primers-data\\re-analysis\\Combined\\count_table_its2+18s_combined.csv", row.names = 1)

sample_data <-read.csv("C:\\Users\\hibaorsud\\Desktop\\3primers-data\\re-analysis\\8_sample_data.csv", row.names = 1)

count_matrix <- as.matrix(count_data)

# Ensure sample names in count_matrix match those in sample_data

count_matrix <- count_matrix[, rownames(sample_data)]

if (!identical(colnames(count_matrix), rownames(sample_data))) {

stop("Sample names in count matrix and sample metadata do not match")

}

dds <- DESeqDataSetFromMatrix(

countData = count_matrix,

colData = sample_data,

design = ~ group # Replace 'group' with your actual covariate

)

dds <- DESeq(dds) # Run DESeq2 normalization

nrow(dds)

vsd <- varianceStabilizingTransformation(dds, blind = FALSE)

#vsd <- vst(dds, blind = FALSE) # Apply variance stabilizing transformation

pca_data <- plotPCA(vsd, intgroup = "group", returnData = TRUE)

# Make group a factor with labeled levels

pca_data$group <- as.factor(pca_data$group) # Convert group to factor

unique(pca_data$group)

pca_data$group <- factor(pca_data$group, levels = c(0, 1), labels = c("CF", "CG"))

# Assume your PCA was done like this:

pca_res <- prcomp(pca_data[, c("PC1", "PC2")], scale. = TRUE)

# Percent variance explained

percent_var <- round(100 * summary(pca_res)$importance[2, 1:2], 1)

names(percent_var) <- c("PC1", "PC2")

# Get percent variance explained

#pca_data <- plotPCA(vst_data, intgroup = "group", returnData = TRUE)

percent_var <- round(100 * attr(pca_data, "percentVar"), 1)

# PCA plot with ellipses and custom colors

ggplot(pca_data, aes(x = PC1, y = PC2, color = group)) +

geom_point(size = 3) +

stat_ellipse(type = "t", size = 1, linetype = "solid") + # Add ellipses

theme_minimal() +

labs(

title = "PCA of DESeq2 for ITS2&18s Taxonomic Data",

x = paste0("PC1 (", percent_var[1], "%)"),

y = paste0("PC2 (", percent_var[2], "%)"),

color = "group"

) +

scale_color_manual(values = c(

"CF" = "#66C2A5",

"CG" = "#FC8D62"

))

**Step 11:** **ALDEx2 PCA**

we employed principal component analysis (PCA) with ALDEx2's aldex.clr function to investigate global compositional disparities among primer sets and clinical groups. The CLR-transformed abundance tables were utilized as input for PCA, facilitating dimensionality reduction while maintaining the ratio-based relationships essential in compositional data. PCA plots demonstrated varied clustering patterns associated with various primer sets and sample groups, illustrating the impact of each primer on community structure resolution. This PCA method enhances the differentially by providing an unsupervised perspective on the entire data structure, identifying nuanced changes in community makeup alongside with primer combination.

**Tool: ALDEx2**

#ALDEx2-PCA;;;;;;;;

#Prepare ALDEx2 Inputs

counts <- as.data.frame(phyloseq::otu_table(ps)) # or your own count matrix

group <- as.factor(phyloseq::sample_data(ps)$group)

group <- as.character(group)

library(ALDEx2)

# denom = "all" or "iqlr" (inter-quartile log-ratio), commonly used

aldex.clr <- aldex.clr(counts, group, mc.samples = 128, denom = "all", verbose = TRUE)

aldex.test <- aldex.ttest(aldex.clr, paired.test = FALSE)

aldex.effect <- aldex.effect(aldex.clr)

# PCA on the median clr values

clr_matrix <- data.frame(t(aldex.clr@reads)) # Transpose to get samples as rows

pca_res <- prcomp(clr_matrix, scale. = TRUE)

# Prepare sample metadata (e.g., group = CF/CG)

metadata <- data.frame(

Sample = rownames(clr_matrix),

Group = conds

)

# Combine PCA scores with metadata

pca_df <- data.frame(pca_res$x, metadata)

# Plot PCA

library(ggplot2)

ggplot(pca_df, aes(x = PC1, y = PC2, color = Group)) +

geom_point(size = 4) +

stat_ellipse(type = "t", size = 1) +

theme_minimal() +

labs(

title = "PCA of ALDEx2 clr-for combined ITS1& 18s",

x = paste0("PC1 (", round(summary(pca_res)$importance[2, 1] * 100, 1), "%)"),

y = paste0("PC2 (", round(summary(pca_res)$importance[2, 2] * 100, 1), "%)")

) +

scale_color_manual(values = c("CF" = "cyan3", "CG" = "tomato"))

# Extract alpha diversity

print(physeq)

library(microbiome)

# Transform to relative abundance if not done

ps_rel <- transform_sample_counts(physeq, function(x) x / sum(x))

# Alpha diversity measures

plot_richness(ps, x = "group", measures = c("Shannon", "Simpson", "Observed")) +

theme_minimal() +

labs(title = "Alpha Diversity Between Groups",

y = "Diversity Index",

x = "Group")

alpha_df <- plot_richness(ps, x = "group", measures = c("Observed", "Shannon", "Simpson"))$data

# Plot with boxplots and points

ggplot(alpha_df, aes(x = group, y = value, fill = group)) +

geom_boxplot(alpha = 0.6, width = 0.6, outlier.shape = NA) +

geom_jitter(width = 0.2, size = 2) +

facet_wrap(~ variable, scales = "free_y") +

stat_compare_means(method = "wilcox.test", label = "p.format") +

scale_fill_manual(values = c("CF" = "cyan3", "CG" = "tomato")) +

theme_minimal() +

labs(

title = "Alpha Diversity Between CF and CG Groups - combined ITS2&18s",

x = "Groups",

y = "Diversity Index"

) +

theme(

strip.text = element_text(face = "bold", size = 12),

axis.text = element_text(size = 10),

axis.title = element_text(face = "bold"),

legend.position = "none"

)

**Performance Metrics and Method Validation**

| **Metric** | **Value/Range** | **Explanation and Justification** |
| --- | --- | --- |
| Taxonomic Resolution | Species level (ITS1/ITS2), genus (18S) | ITS1 and ITS2 yielded species-level resolution for significant taxa, such as *Candida albicans, Malassezia furfur,* and *Rhodotorula mucilaginosa*, as seen in LEfSe and ALDEx2 LDA results. - 18S often resolved greater unclassified Eukaryota, indicating its conserved characteristics. |
| LOD (Limit of Detection) | ~10–100 copies/sample (based on ITS) | - This is based on previous fungal barcoding literature (Bellemain et al. 2010), and corresponds with our finding of the detection of low-abundance species such as *Exophiala* and *Penicillium* in ITS datasets, not always present in 18S alone. |
| Accuracy | >90% classification in real societies | The utilization of curated databases (SILVA and UNITE) facilitated precise taxonomic classifications, as demonstrated by the constant identification of predominant fungus across markers such as *C. albicans, S. cerevisiae, C. parapsilosis*. Aligns with literature-based benchmarks for ITS regions (Nilsson et al., 2019). |
| Precision | Consistent detection across replicates | - Numerous markers repeatedly consistently identified key taxa (e.g., *C. albicans* in CG group), validating reproducibility. - Alpha diversity and PCA plots showed replicates clustering by group, supporting internal precision. |
| Best Discrimination | ITS1+ITS2 combination | - This pairing gave the widest effect size range and multiple significant markers, especially in LEfSe and ALDEx2. - Clarity in separating CF and CG groups was highest here (supported by PCA and volcano plots). |

**Applications**

- Methodological validation of multi-marker integration pipelines to improve fungal community resolution in mycobiome research.
- Targeted detection of infrequent or low-abundance gut fungi, even within small-sample cohort.
- Comparative mycobiome profiling between disease states (e.g., cancer versus cancer-free individuals), facilitating the discovery of condition-associated taxa.

**References:**

1. RStudio. *bookdown: Authoring Books and Technical Documents with R Markdown*. GitHub; Available from: <https://github.com/rstudio/bookdown>
2. Microbiome Project. *Microbiome R Package* [Internet]. GitHub; [cited 2025 Mar 20]. Available from: https://github.com/microbiome
3. Bioconductor. BiocVersion: Bioconductor Version Information [Internet]. Bioconductor; Available from: <https://www.bioconductor.org/packages/release/bioc/html/BiocVersion.html>
4. Bellemain E, Carlsen T, Brochmann C, Coissac E, Taberlet P, Kauserud H. ITS as an environmental DNA barcode for fungi: An in silico approach reveals potential PCR biases. BMC Microbiol. 2010;10:189. doi:10.1186/1471-2180-10-189
5. Nilsson RH, Larsson KH, Taylor AFS, Bengtsson‐Palme J, Jeppesen TS, Schigel D, Kennedy P, Picard K, Glöckner FO, Tedersoo L, Saar I, Koljalg U, Abarenkov K. The UNITE database for molecular identification of fungi: Handling dark taxa and parallel taxonomic classifications. Nucleic Acids Res. 2019;47(D1):D259–D264.

**Software References:**

**R packages:**

**dada2:**

Callahan BJ, McMurdie PJ, Rosen MJ, Han AW, Johnson AJA, Holmes SP. DADA2: High-resolution sample inference from Illumina amplicon data. Nature Methods. 2016;13(7):581–3. doi:10.1038/nmeth.3869. [Bioconductor - dada2](https://www.bioconductor.org/packages/release/bioc/html/dada2.html?utm_source=chatgpt.com/)

**BiocManager**
Morgan M, Ramos M. BiocManager: Access the Bioconductor Project Package Repository. R package version 1.30.26. 2025. Available from: https://bioconductor.github.io/BiocManager/

**Biocstyle:**

Oleś A (2025). BiocStyle: Standard styles for vignettes and other Bioconductor documents. [doi:10.18129/B9.bioc.BiocStyle](https://doi.org/10.18129/B9.bioc.BiocStyle), R package version 2.36.0, <https://bioconductor.org/packages/BiocStyle>.

**ggplot2**

Wickham H. ggplot2: Elegant Graphics for Data Analysis. New York: Springer-Verlag; 2016. Available from: <https://ggplot2.tidyverse.org>

**miaV**

Borman T, Ernst F, Shetty S, Lahti L (2025). mia: Microbiome analysis. [doi:10.18129/B9.bioc.mia](https://doi.org/10.18129/B9.bioc.mia), R package version 1.16.1, <https://bioconductor.org/packages/mia>.

**Vegan**
Oksanen J, Simpson GL, Blanchet FG, Kindt R, Legendre P, Minchin PR, et al. vegan: Community Ecology Package. R package version 2.7-1. 2025. Available from: <https://cran.r-project.org/package=vegan>.

**ALDEx2**

Fernandes AD, Macklaim JM, Linn TG, Reid G, Gloor GB. ANOVA-Like Differential Gene Expression Analysis of Sequence Count Data. R package version 1.24.0. 2013. Available from: <https://bioconductor.org/packages/release/bioc/html/ALDEx2.html>

**DESeq2**
Love MI, Huber W, Anders S. Moderated estimation of fold change and dispersion for RNA-seq data with DESeq2. Genome Biology. 2014;15(12):550. doi:10.1186/s13059-014-0550-8. <http://www.bioconductor.org/packages/release/bioc/html/DESeq2.html>.

**metagenomeSeq**
Paulson JN, Stine OC, Bravo HC, Pop M. Analysis of 16S rRNA-based studies. R package version 1.24.0. 2013. Available from: <https://bioconductor.org/packages/release/bioc/html/metagenomeSeq.html>

**Python libraries:**

**Pandas**
McKinney W. pandas: a foundational Python library for data analysis and statistics. Python for High Performance and Scientific Computing. 2011;14:1–9.

**NumPy**
Harris CR, Millman KJ, van der Walt SJ, Gommers R, Virtanen P, Cournapeau D, et al. Array programming with NumPy. Nature. 2020;585(7825):357–62. doi:10.1038/s41586-020-2649-2.

**Seaborn**
Waskom ML. seaborn: statistical data visualization. Journal of Open Source Software. 2021;6(60):3021. doi:10.21105/joss.03021.

**Matplotlib**
Hunter JD. Matplotlib: A 2D graphics environment. Computing in Science & Engineering. 2007;9(3):90–5. doi:10.1109/MCSE.2007.55.
