# Supplementary material for: Multi-marker comparative analysis of 18S, ITS1, and ITS2 primers for human gut mycobiome profiling
Source: Front Bioinform. 2025 Nov 19;5:1690766. doi: 10.3389/fbinf.2025.1690766 (PMC12672528; doi:10.3389/fbinf.2025.1690766)
Supplement: Supplementary file 3 [file DataSheet1.pdf]

Supplementary materials

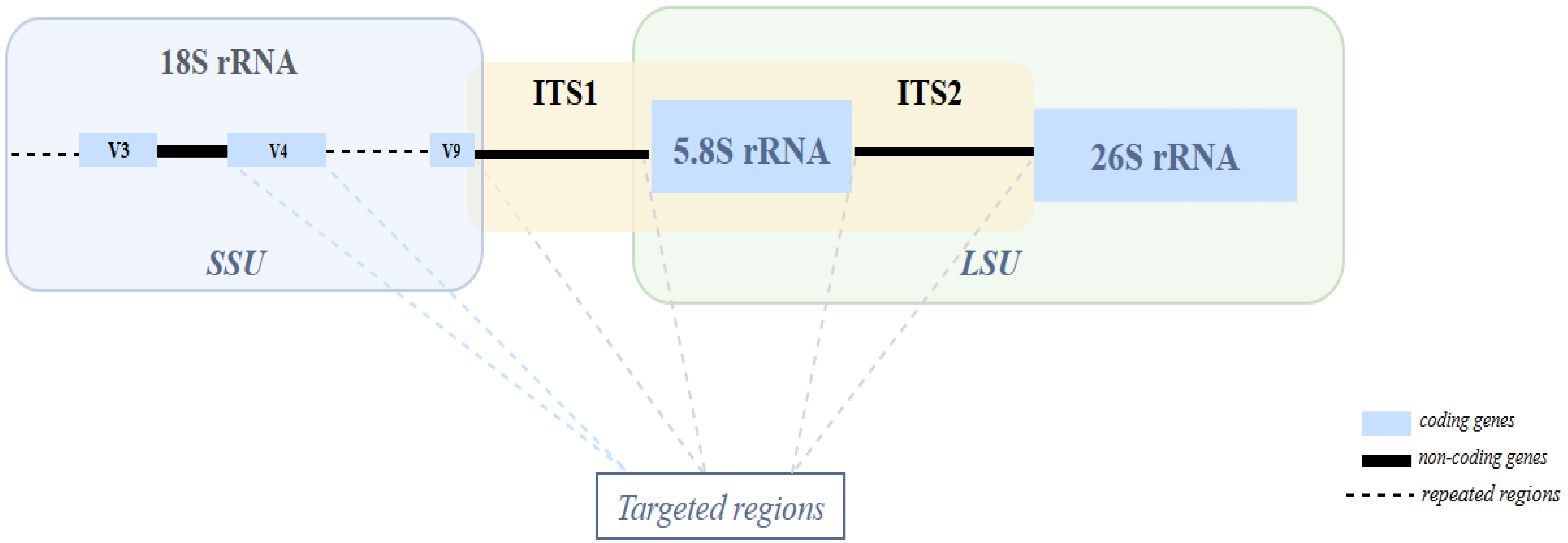

Figure S1: The ITS region as spacers between the ribosomal subunit sequences, the ITS region including ITS1 and ITS2, the spacer genes allocated between the small-subunit rRNA and large-subunit rRNA **[18-19]**.

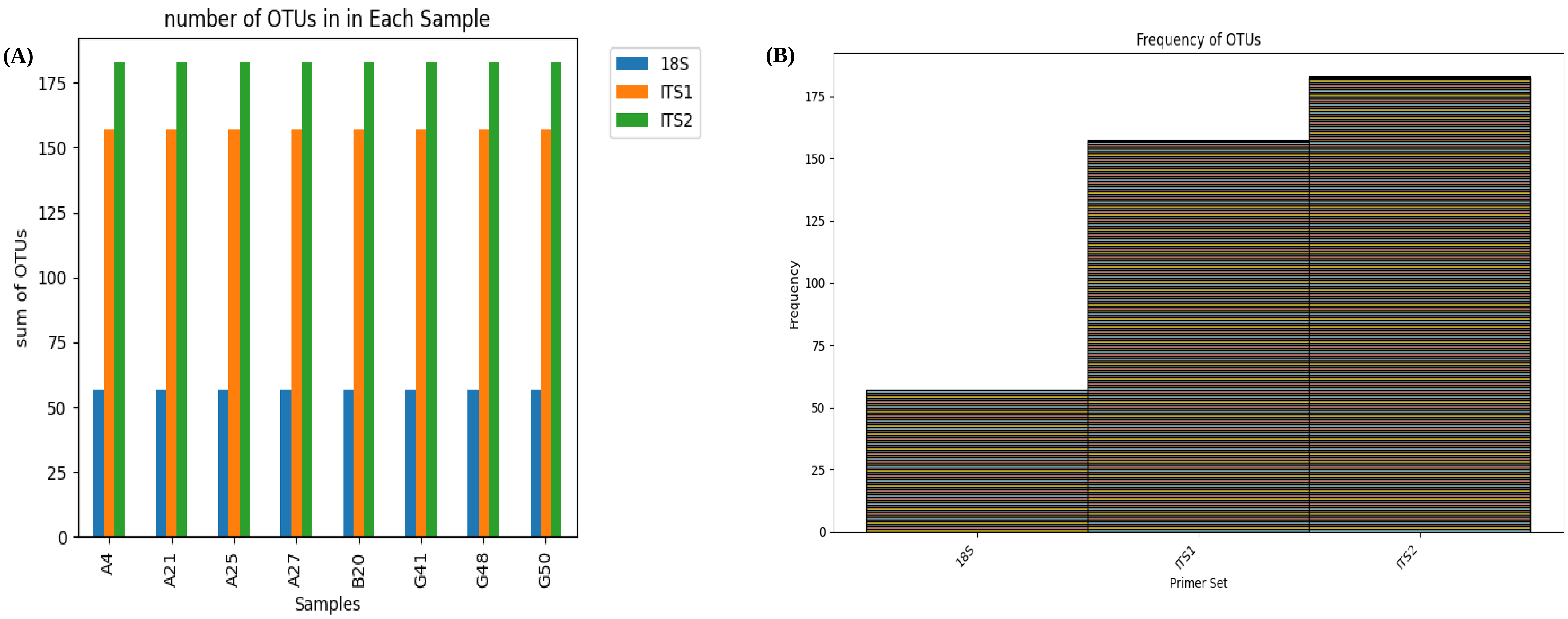

Figure S2: OTUs distribution. The plots provide multiple charts pertaining to OTUs distribution across various primer sets and sample groups; **(A)** bar plot shows the quantity of OTUs in each sample by primer set (18S, ITS1, and ITS2). the y-axis denotes the quantity of OTUs, whilst the x-axis illustrates the samples (A4, A21, A25, A27, B20, G41, G48, G50). **(B)** Frequency of OTUs; the histogram illustrates the frequency of OTUs across several primer sets (18S, ITS1, ITS2). The y-axis denotes the frequency of occurrence, while the x-axis illustrates the primer sets.

(A) Family-level taxa prevalence generated by 3-primer: ITS1

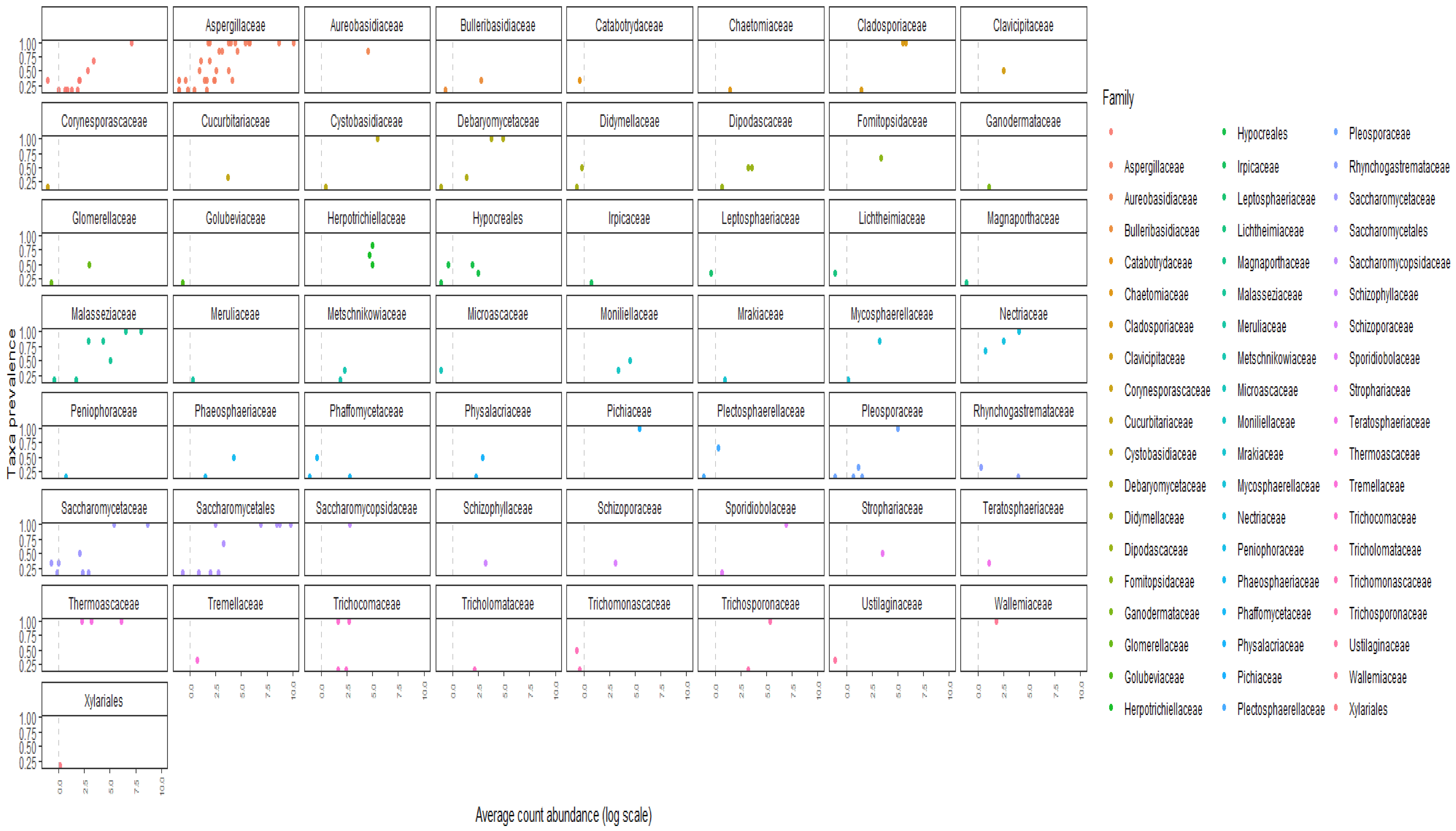

Figure S3. Taxa prevalence: (A), (B), (C) graphs represent the average abundance counts of microbial taxa, presumably at the family level, represented on a logarithmic scale. These charts illustrate the heterogeneity of various microbial families across several samples and primer sets. expands the scope of family-level taxa and illustrates their distribution across samples with logarithmically adjusted abundances.

(B) Family-level taxa prevalence generated by 3-primer: ITS2

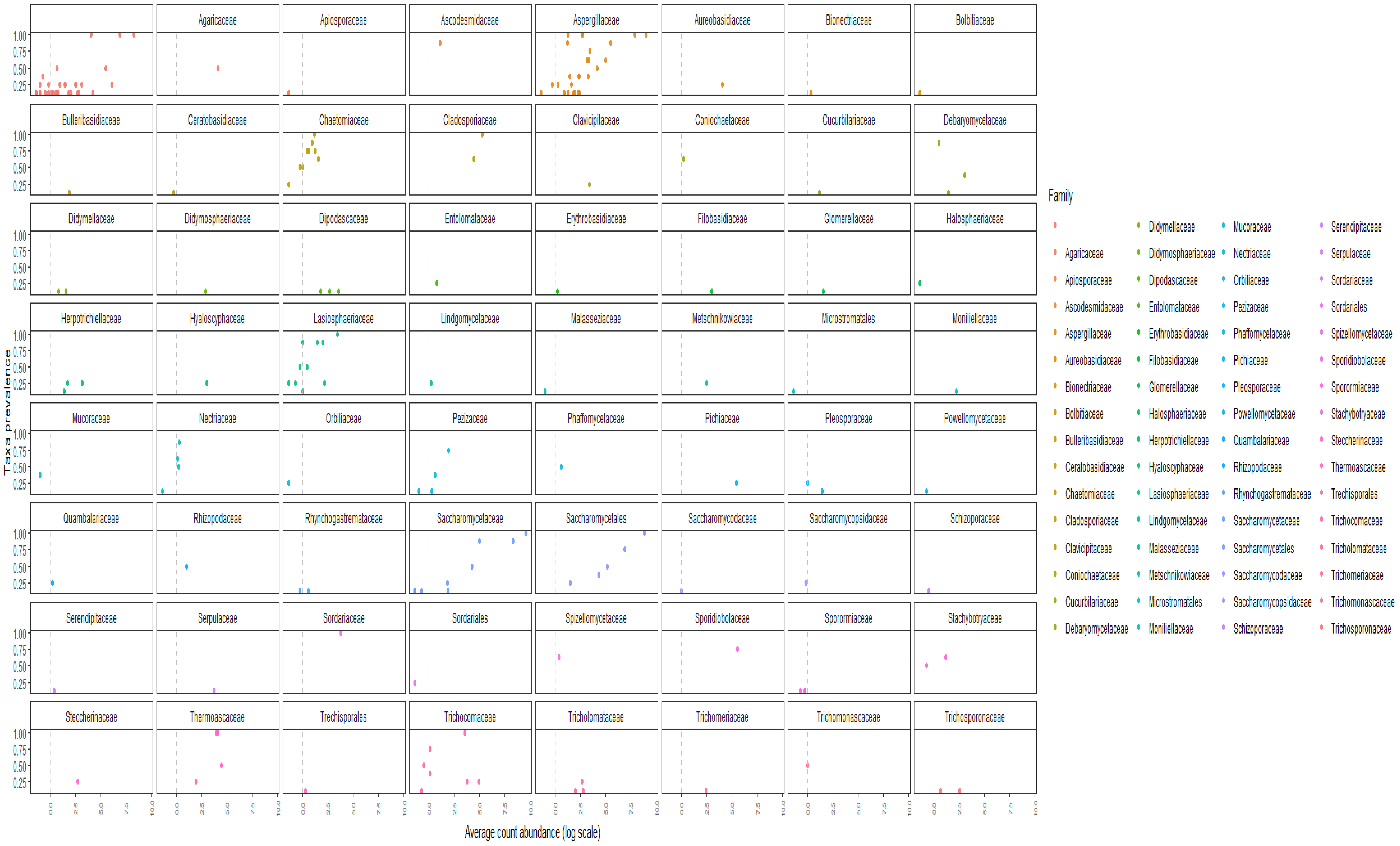

Figure S3. Taxa prevalence: (A), (B), (C) graphs represent the average abundance counts of microbial taxa, presumably at the family level, represented on a logarithmic scale. These charts illustrate the heterogeneity of various microbial families across several samples and primer sets. expands the scope of family-level taxa and illustrates their distribution across samples with logarithmically adjusted abundances.

(C) Family-level taxa prevalence generated by 3-primer: 18s

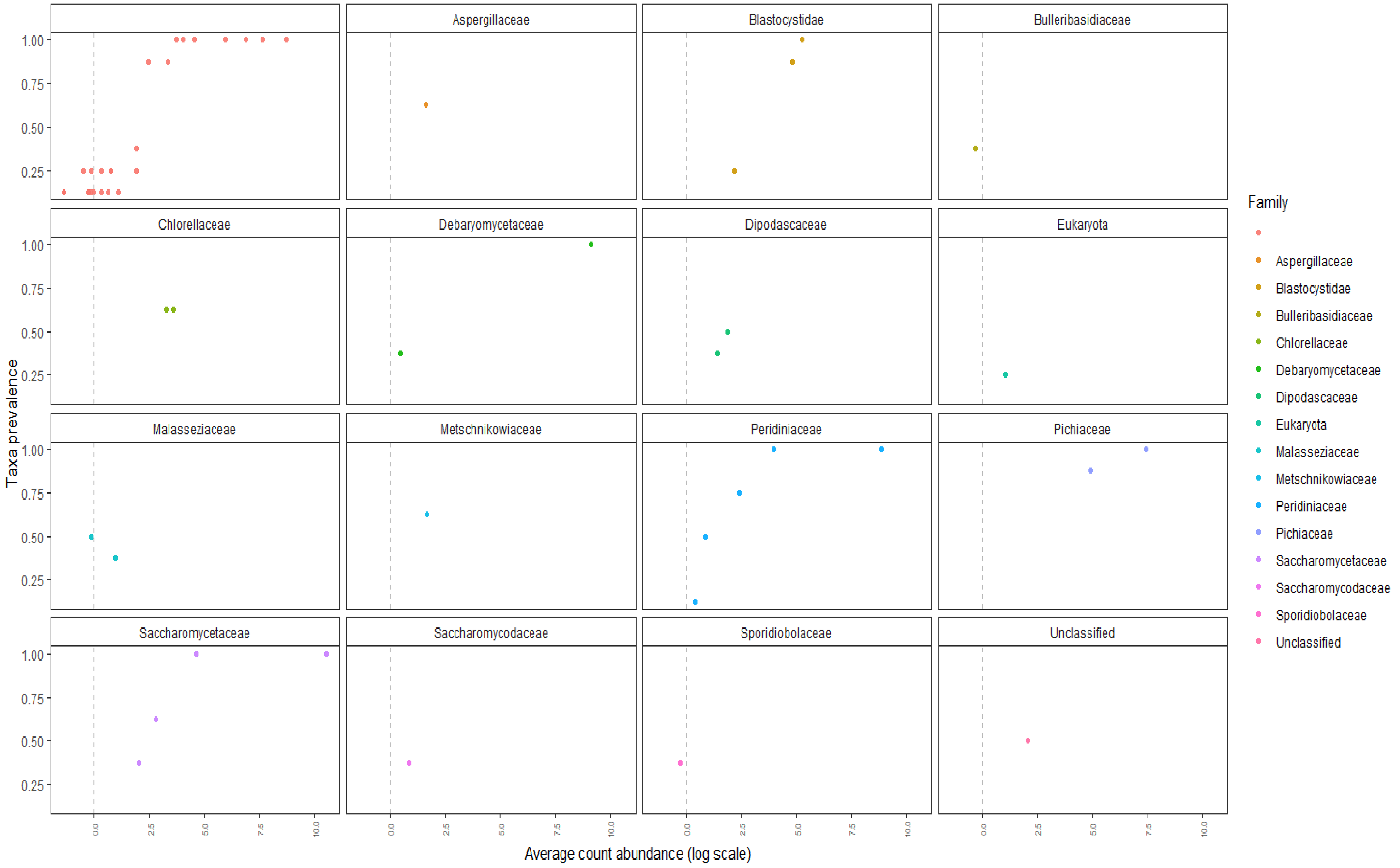

Figure S3. Taxa prevalence: (A), (B), (C) graphs represent the average abundance counts of microbial taxa, presumably at the family level, represented on a logarithmic scale. These charts illustrate the heterogeneity of various microbial families across several samples and primer sets. expands the scope of family-level taxa and illustrates their distribution across samples with logarithmically adjusted abundances.

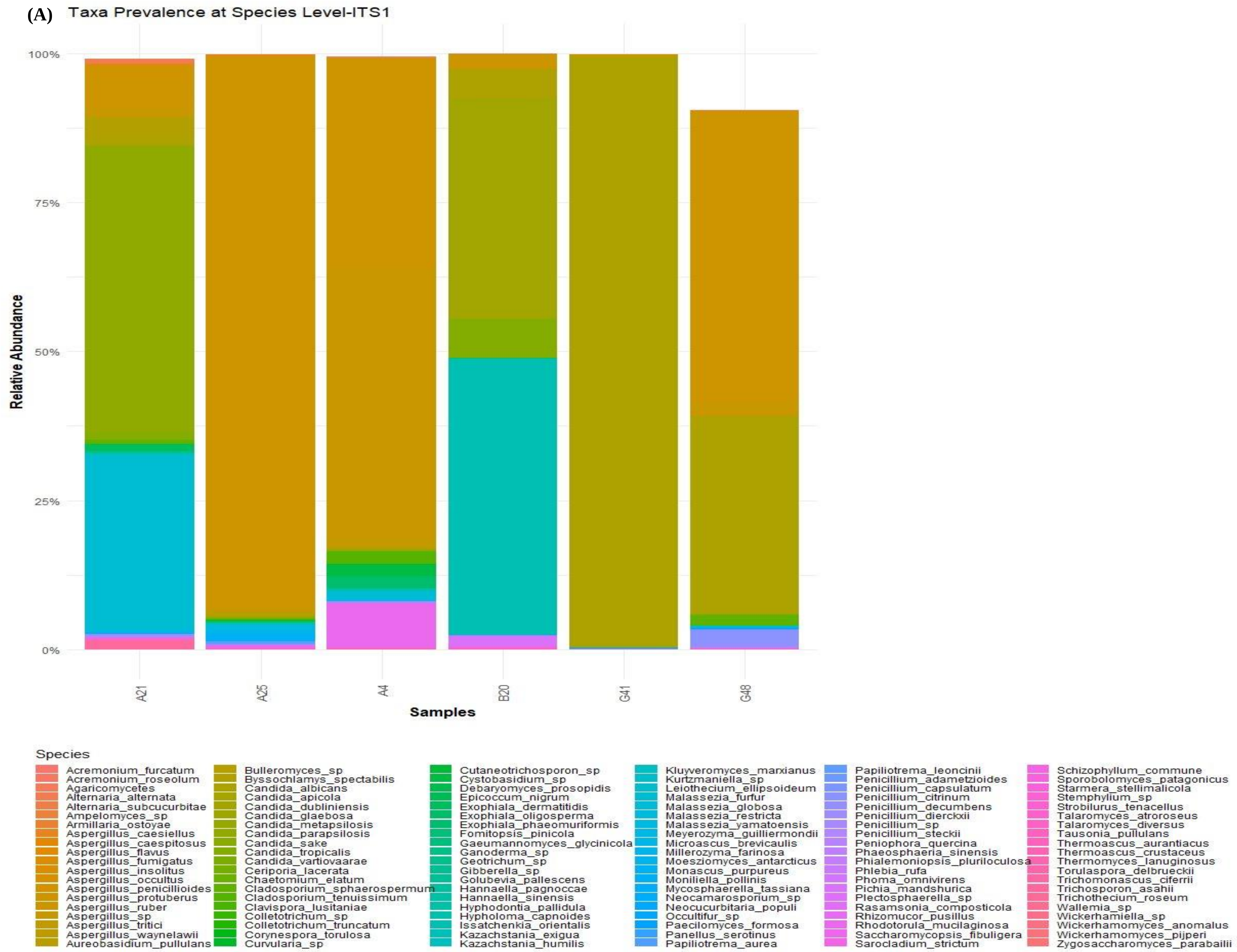

Figure S4: Taxa prevalence. The stack bars (A), (B), (C) represent the average abundance counts of microbial taxa, presumably at the species level, represented on a percentage scale. Illustrating the heterogeneity of various microbial communities across several samples, expanding the scope to species-level taxa and illustrating their distribution among samples with abundance percentages.

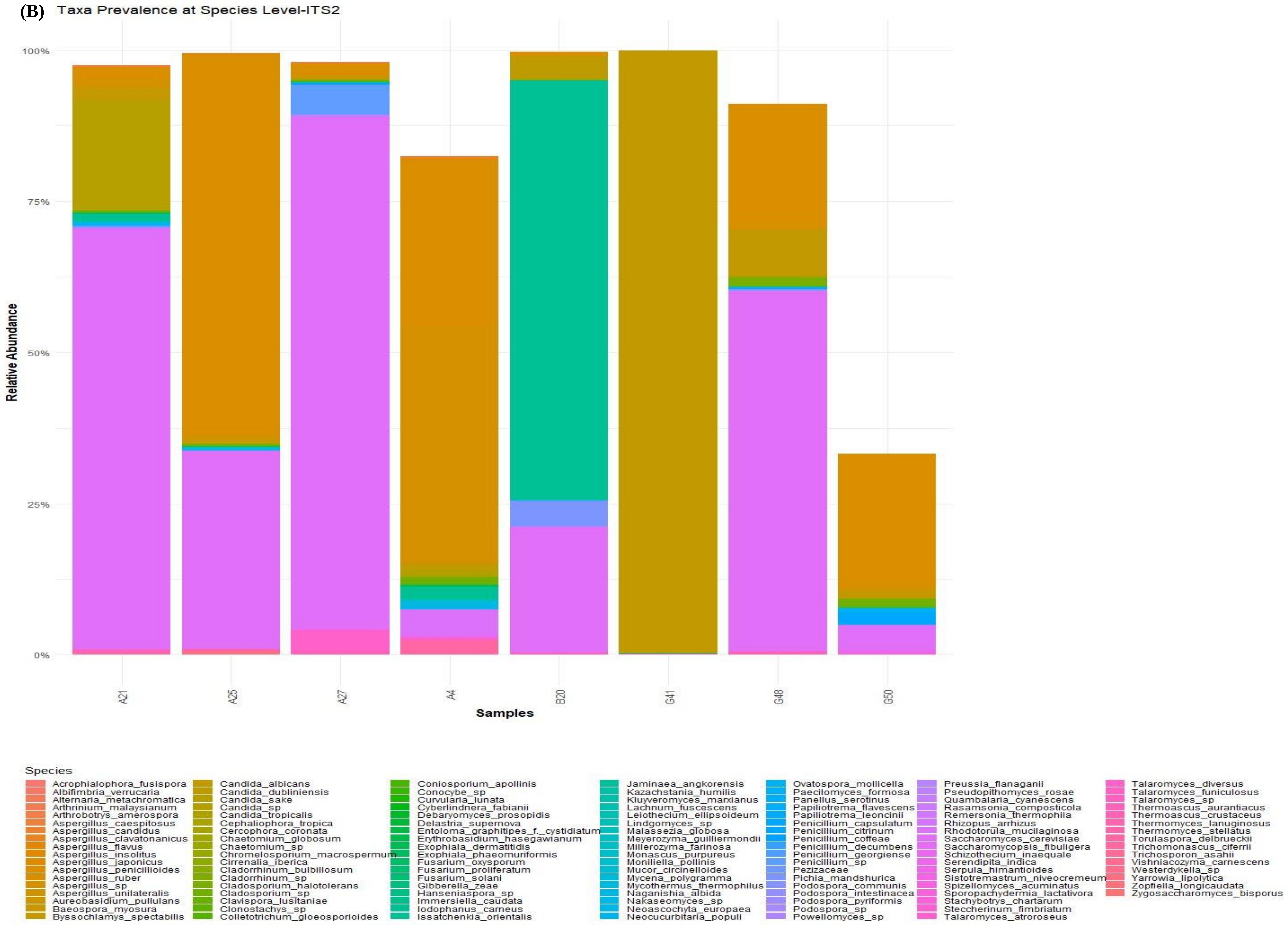

Figure S4: Taxa prevalence. The stack bars (A), (B), (C) represent the average abundance counts of microbial taxa, presumably at the species level, represented on a percentage scale. Illustrating the heterogeneity of various microbial communities across several samples, expanding the scope to species-level taxa and illustrating their distribution among samples with abundance percentages.

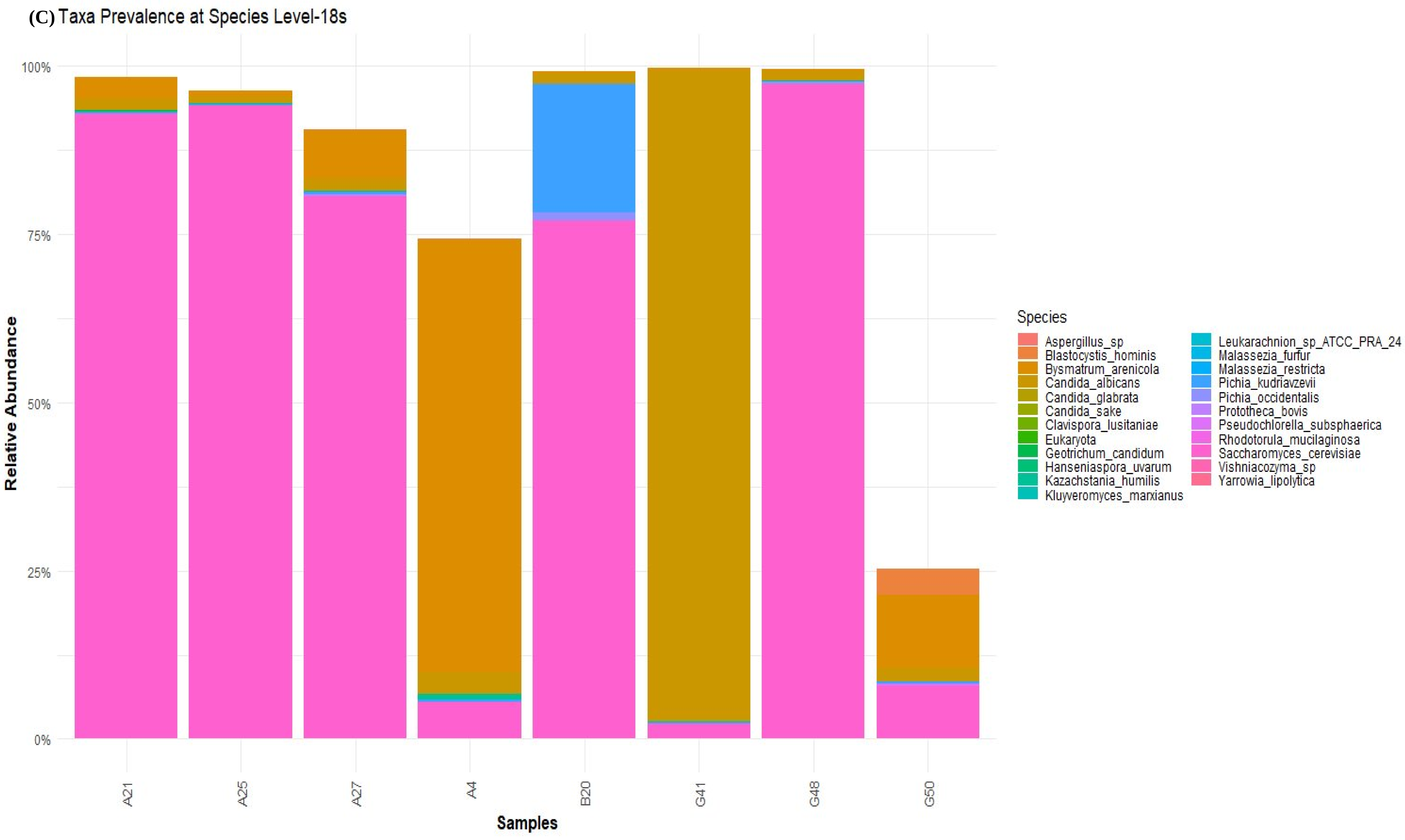

Figure S4: Taxa prevalence. The stack bars (A), (B), (C) represent the average abundance counts of microbial taxa, presumably at the species level, represented on a percentage scale. Illustrating the heterogeneity of various microbial communities across several samples, expanding the scope to species-level taxa and illustrating their distribution among samples with abundance percentages.

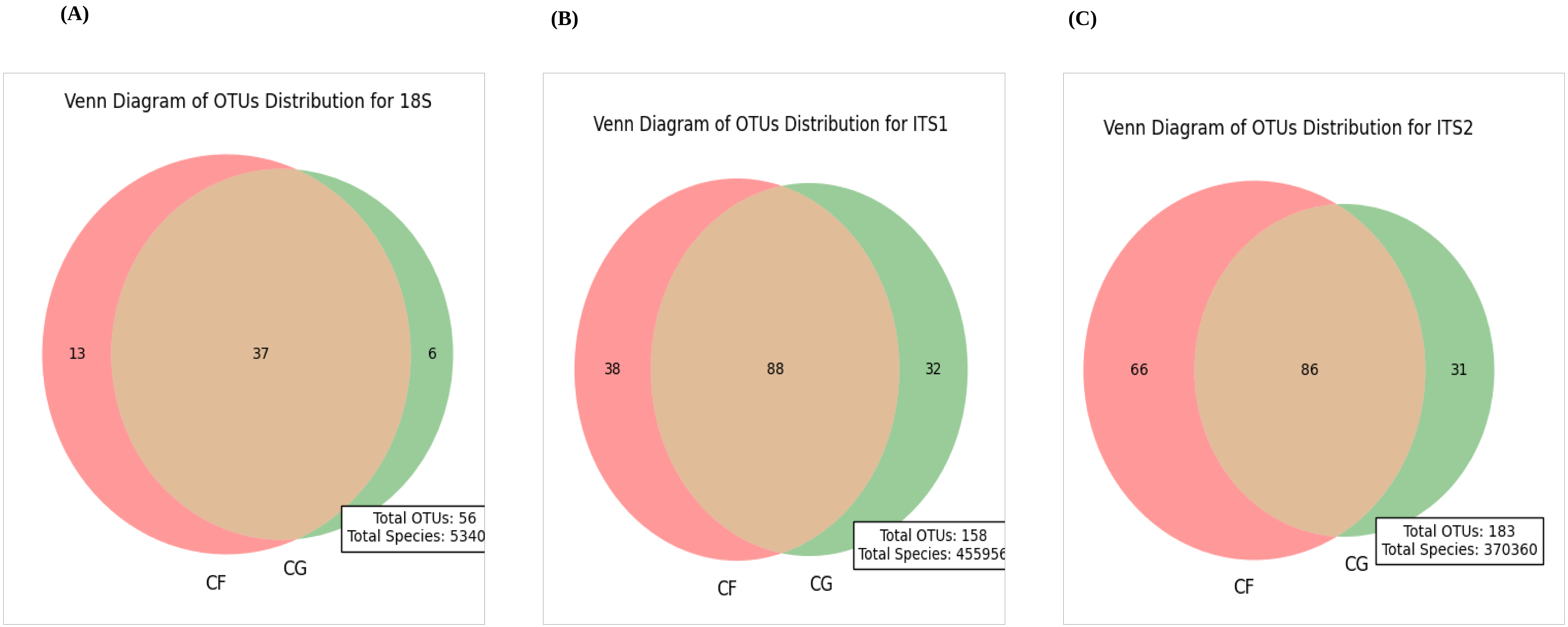

Figure S5: Venn Diagrams depicting the distribution of OTUs for 18S, ITS1, and ITS2 exhibit the overlap of OTUs between the two CF (cancer-free group) and CG (cancer group). In the 18S primer set, 13 OTUs were unique to CF, 6 were unique to CG, and 37 overlapped, totaling 56. The ITS1 primer set produced 158 OTUs: 38 CF-specific, 32 CG-specific, and 88 shared. A total of 183 OTUs were found in the ITS2 primer set, including 66 unique to CF, 31 to CG, and 86 shared (File S2).

(A) ITS1

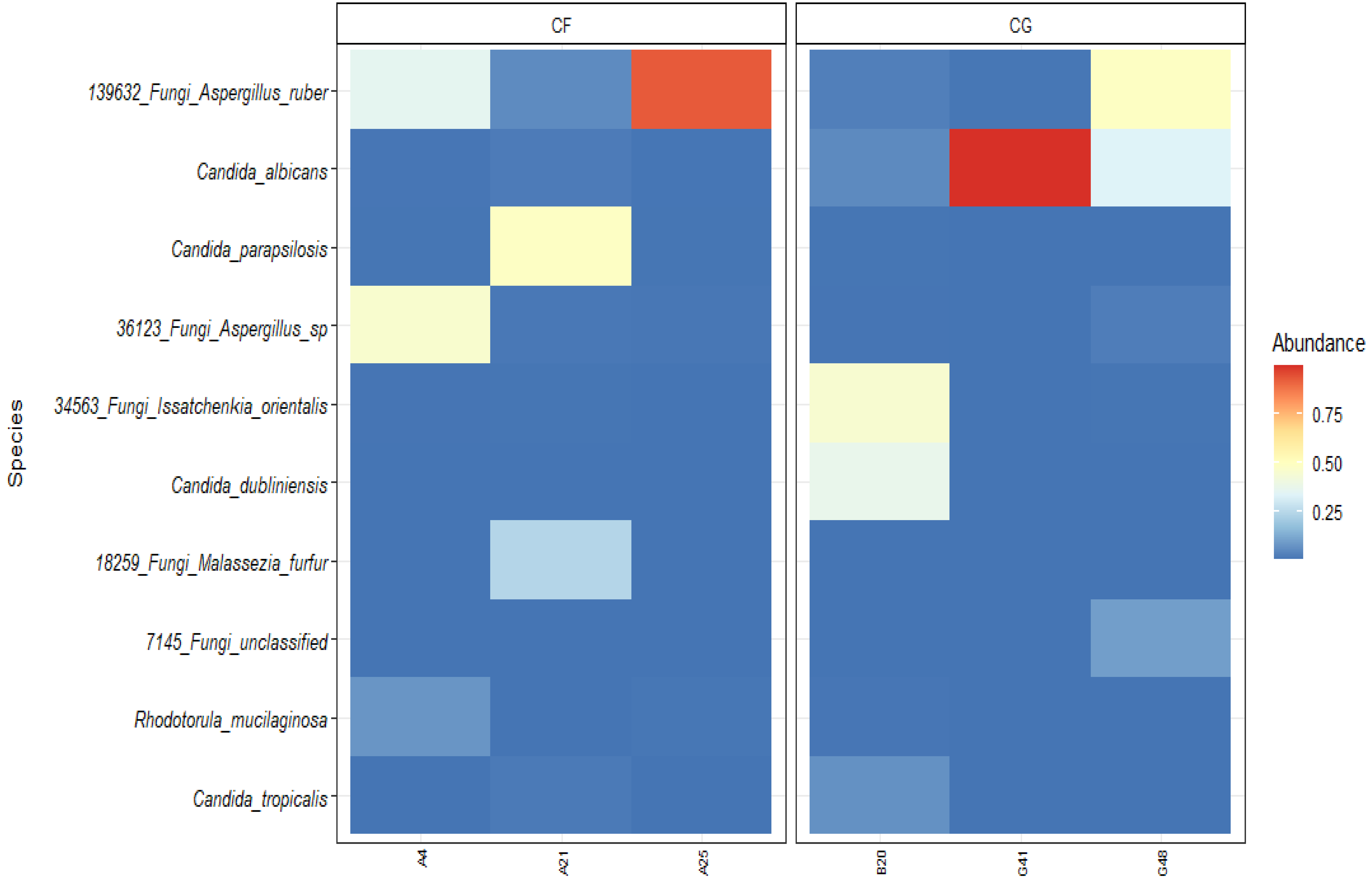

Figure S6. Differential analysis: The heatmaps (A), (B), (C) illustrate the prevalence of particular species within the two groups across the three primer sets. *Candida albicans* exhibited high differential abundance with the three primer sets, signifying differential abundance between the groups. *Saccharomyces cerevisiae* showed greater abundance with 18S but appeared more differentiated with ITS2, although having equivalent total abundance. 18S displayed more unclassified species. The color gradient facilitates the visualization of species abundance and their variation across different situations.

(B) ITS2

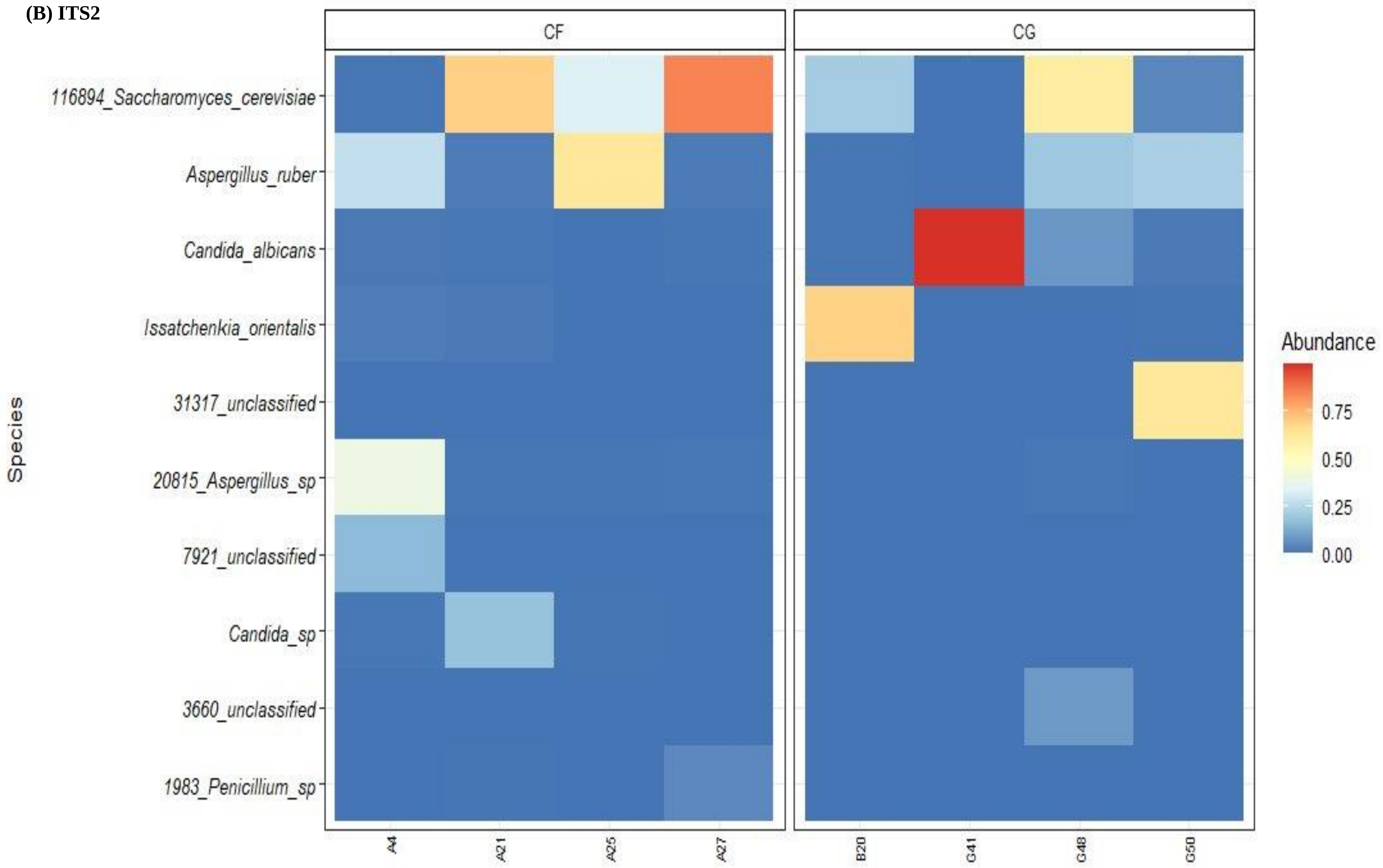

Figure S6. Differential analysis: The heatmaps (A), (B), (C) illustrate the prevalence of particular species within the two groups across the three primer sets. *Candida albicans* exhibited high differential abundance with the three primer sets, signifying differential abundance between the groups. *Saccharomyces cerevisiae* showed greater abundance with 18S but appeared more differentiated with ITS2, although having equivalent total abundance. 18S displayed more unclassified species. The color gradient facilitates the visualization of species abundance and their variation across different situations.

(C) 18S

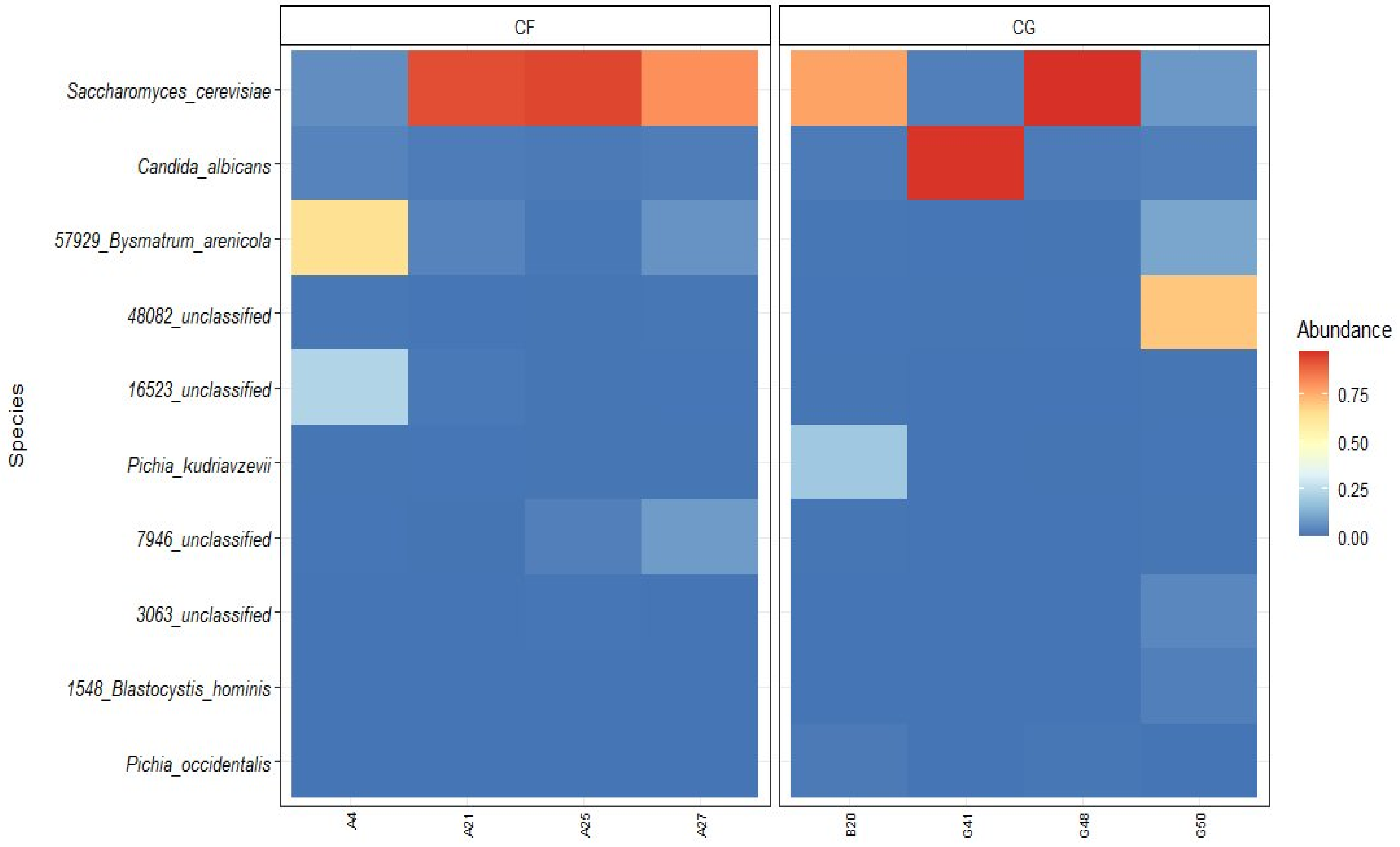

Figure S6. Differential analysis: The heatmaps (A), (B), (C) illustrate the prevalence of particular species within the two groups across the three primer sets. *Candida albicans* exhibited high differential abundance with the three primer sets, signifying differential abundance between the groups. *Saccharomyces cerevisiae* showed greater abundance with 18S but appeared more differentiated with ITS2, although having equivalent total abundance. 18S displayed more unclassified species. The color gradient facilitates the visualization of species abundance and their variation across different situations.

(A) ITS1-ITS2

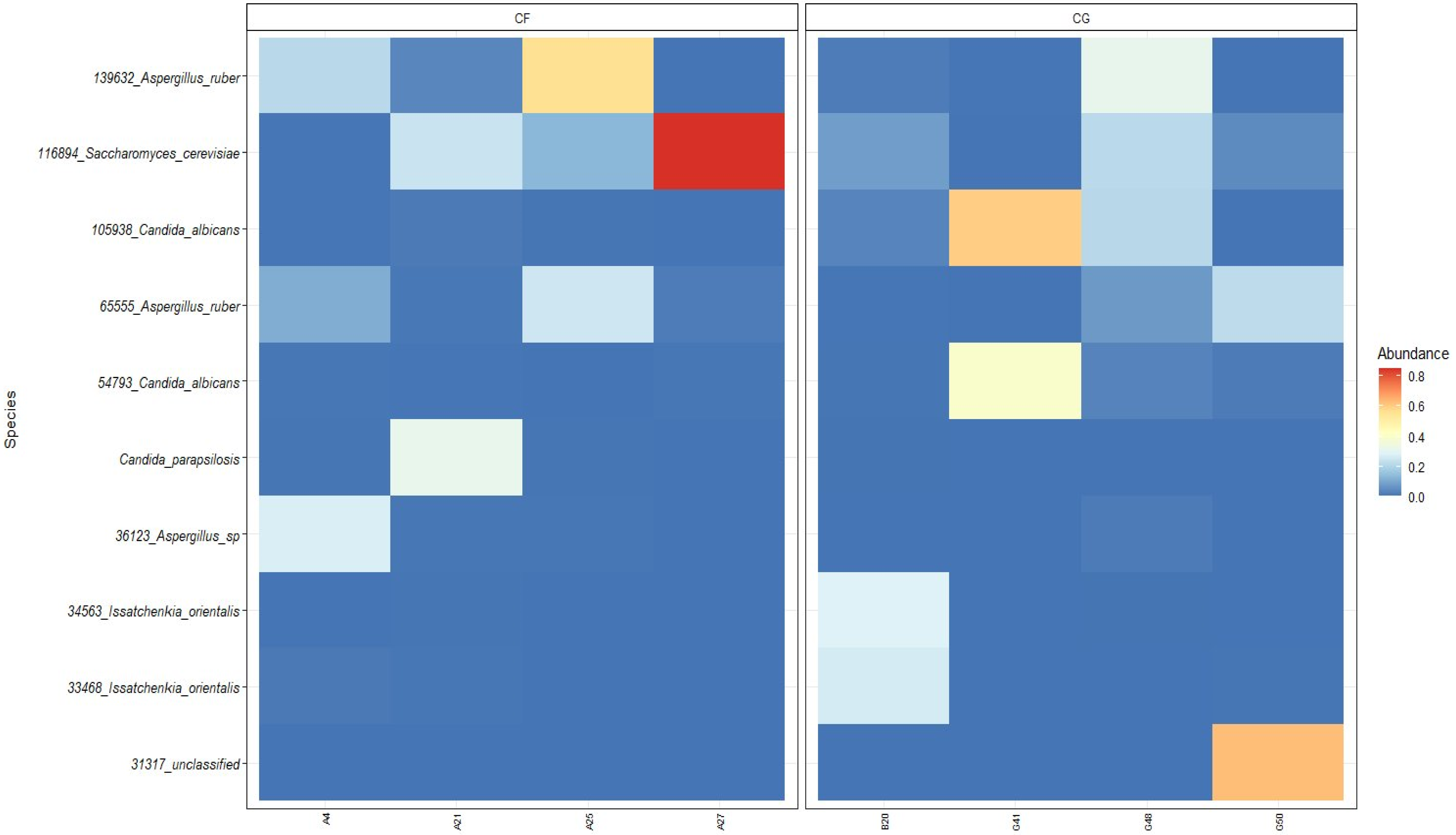

Figure S7. Differential analysis: The heatmaps (A), (B), (C) illustrate the prevalence of particular species within the two groups across the combined datasets (ITS1-ITS2, ITS1-18S, ITS2-18S, ITS1-ITS2-18S). CF group consistently shows elevated abundance of *Saccharomyces cerevisiae* and *Aspergillus ruber*, especially in the ITS1-ITS2 and ITS2-18S datasets. The CG group is characterized by greater prevalence of unclassified taxa such as unclassified Basidiomycota, suggesting more taxonomically diverse or less studied communities.

(B) ITS1-18S

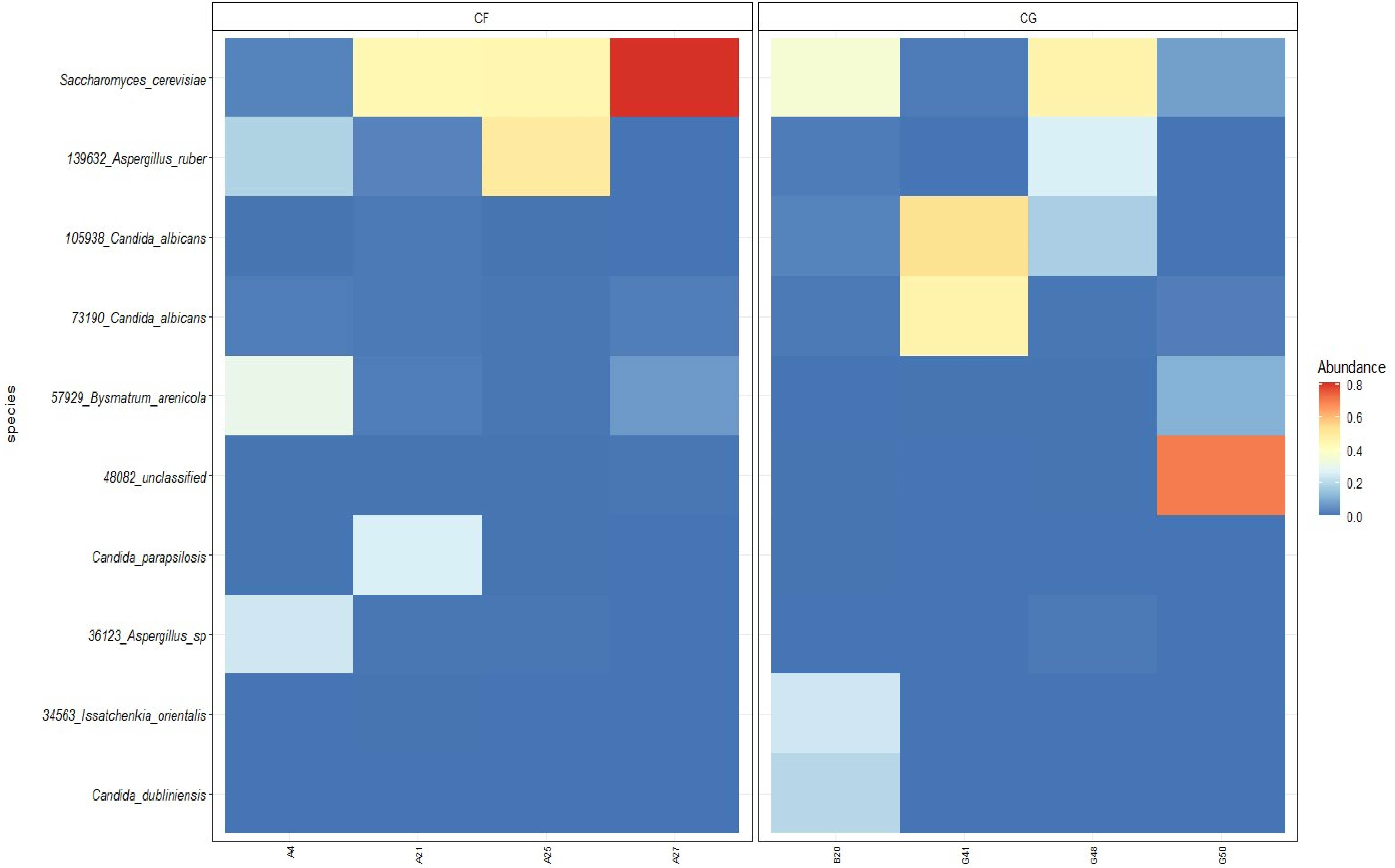

Figure S7. Differential analysis: The heatmaps (A), (B), (C) illustrate the prevalence of particular species within the two groups across the combined datasets (ITS1-ITS2, ITS1-18S, ITS2-18S, ITS1-ITS2-18S). CF group consistently shows elevated abundance of *Saccharomyces cerevisiae* and *Aspergillus ruber*, especially in the ITS1-ITS2 and ITS2-18S datasets. The CG group is characterized by greater prevalence of unclassified taxa such as unclassified Basidiomycota, suggesting more taxonomically diverse or less studied communities.

(C) ITS2-18S

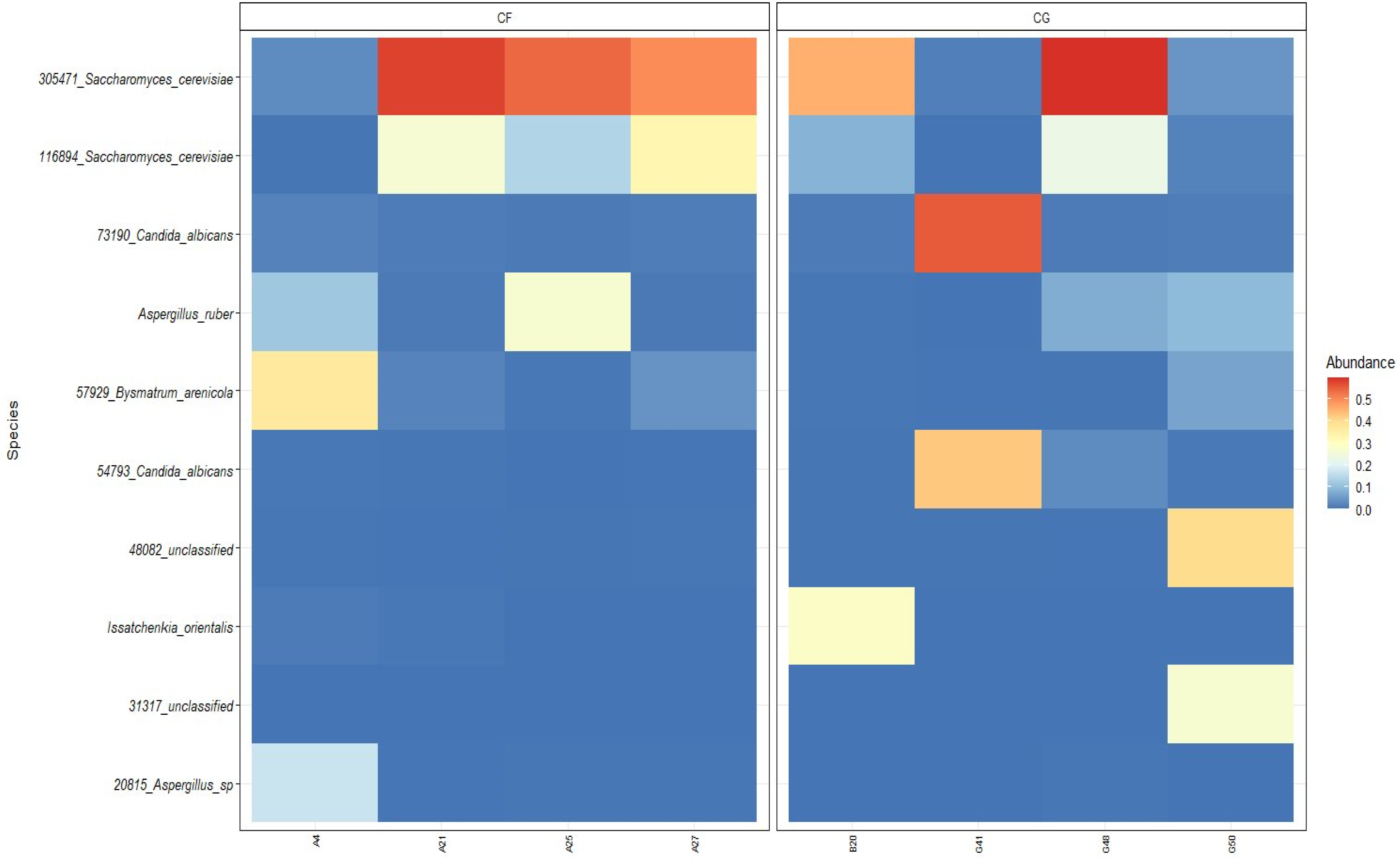

Figure S7. Differential analysis: The heatmaps (A), (B), (C) illustrate the prevalence of particular species within the two groups across the combined datasets (ITS1-ITS2, ITS1-18S, ITS2-18S, ITS1-ITS2-18S). CF group consistently shows elevated abundance of *Saccharomyces cerevisiae* and *Aspergillus ruber*, especially in the ITS1-ITS2 and ITS2-18S datasets. The CG group is characterized by greater prevalence of unclassified taxa such as unclassified Basidiomycota, suggesting more taxonomically diverse or less studied communities.

(D) **ITS1-ITS2-18S**

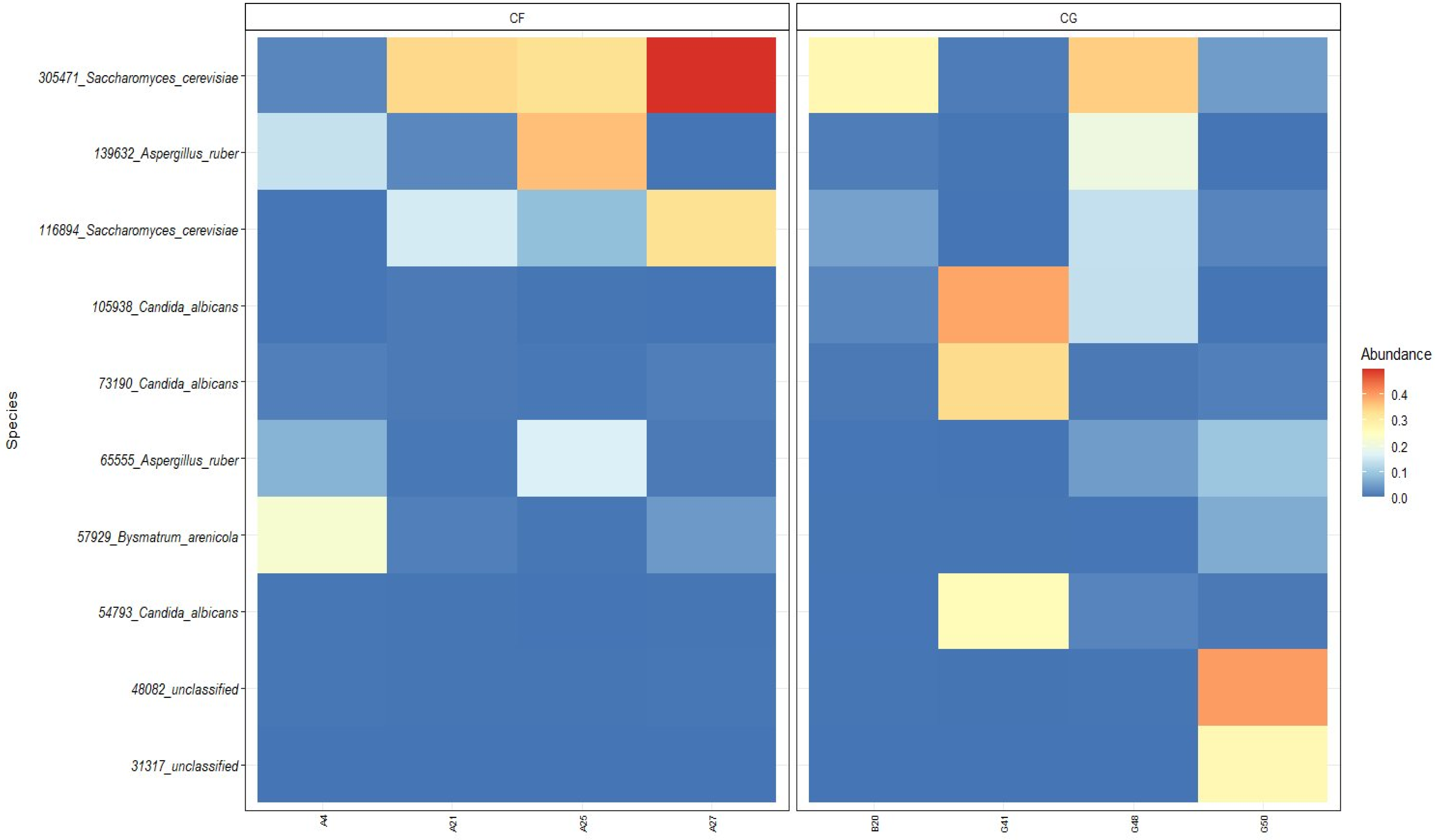

Figure S7. Differential analysis: The heatmaps (A), (B), (C) illustrate the prevalence of particular species within the two groups across the combined datasets (ITS1-ITS2, ITS1-18S, ITS2-18S, ITS1-ITS2-18S). CF group consistently shows elevated abundance of *Saccharomyces cerevisiae* and *Aspergillus ruber*, especially in the ITS1-ITS2 and ITS2-18S datasets. The CG group is characterized by greater prevalence of unclassified taxa such as unclassified Basidiomycota, suggesting more taxonomically diverse or less studied communities.

(A) ITS1

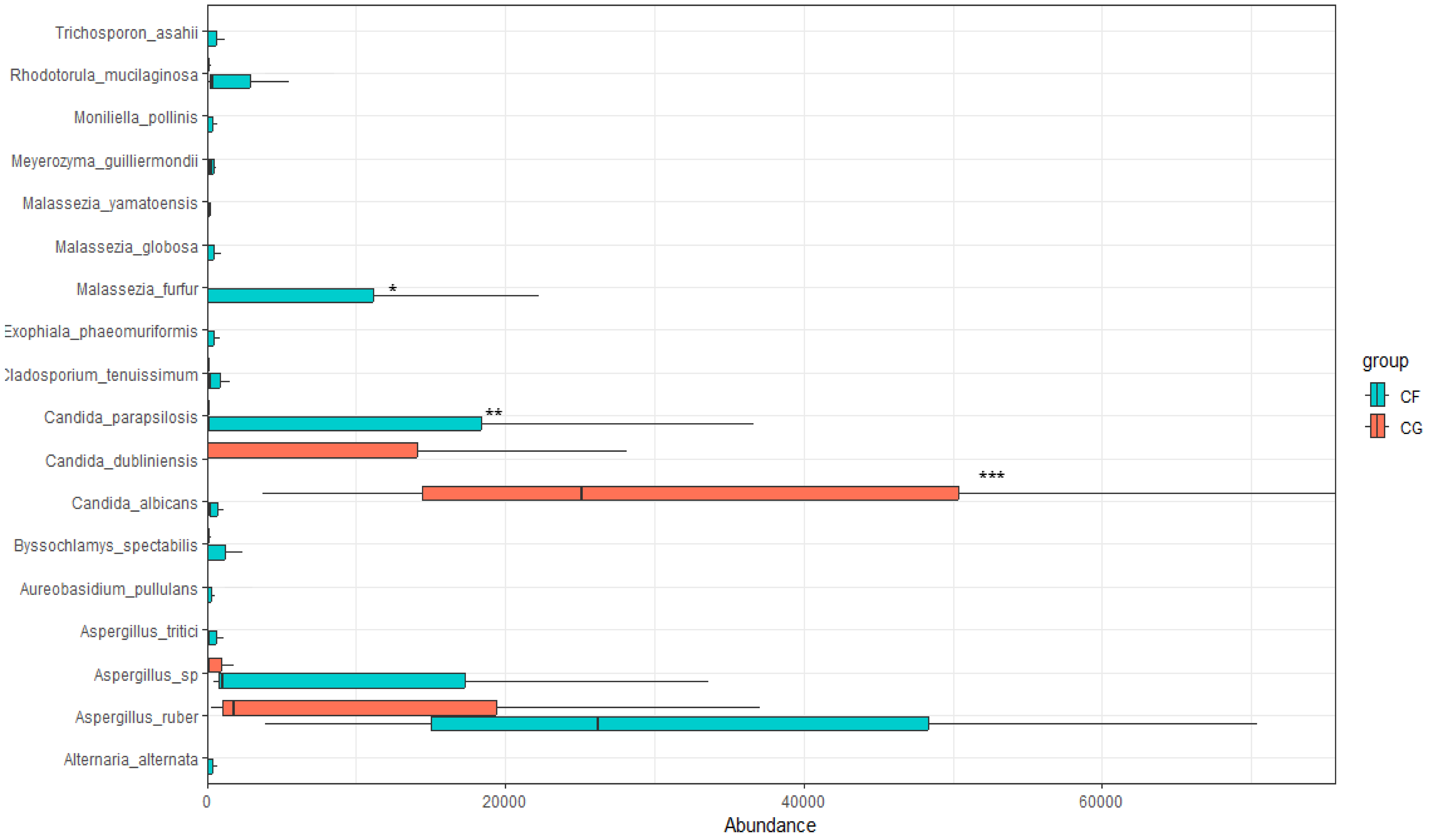

Figure S8. LDA abundance of Amplicon-based conventional primers: The plots (A), (B), (C) show Linear Discriminant Analysis (LDA) for (ITS1, ITS2, 18S) is utilized to distinguish groups based on the abundance of microbial species. The LDA results underscore particular microbial species that differentiate the groupings, including ITS1, ITS2, and 18S. The bar plots illustrate the LDA scores, highlighting species that markedly differentiate the groups, as evidenced by elevated LDA values. CG in red and CF in blue exhibit divergent patterns of enrichment for various species. An asterisk indicates species that are significantly differentially abundant.

(B) ITS2

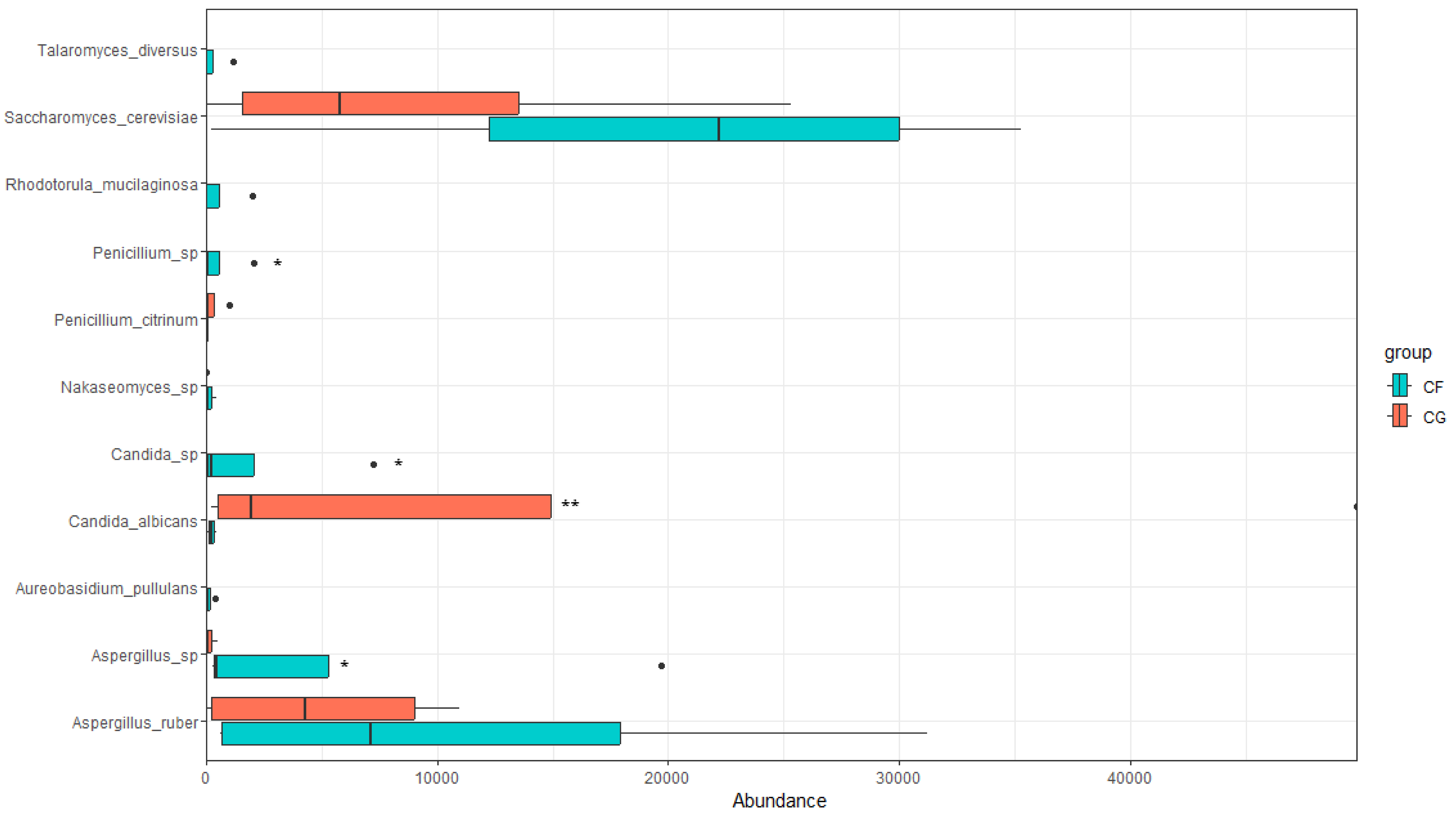

Figure S8. LDA abundance of Amplicon-based conventional primers: The plots (A), (B), (C) show Linear Discriminant Analysis (LDA) for (ITS1, ITS2, 18S) is utilized to distinguish groups based on the abundance of microbial species. The LDA results underscore particular microbial species that differentiate the groupings, including ITS1, ITS2, and 18S. The bar plots illustrate the LDA scores, highlighting species that markedly differentiate the groups, as evidenced by elevated LDA values. CG in red and CF in blue exhibit divergent patterns of enrichment for various species. An asterisk indicates species that are significantly differentially abundant.

(C) 18S

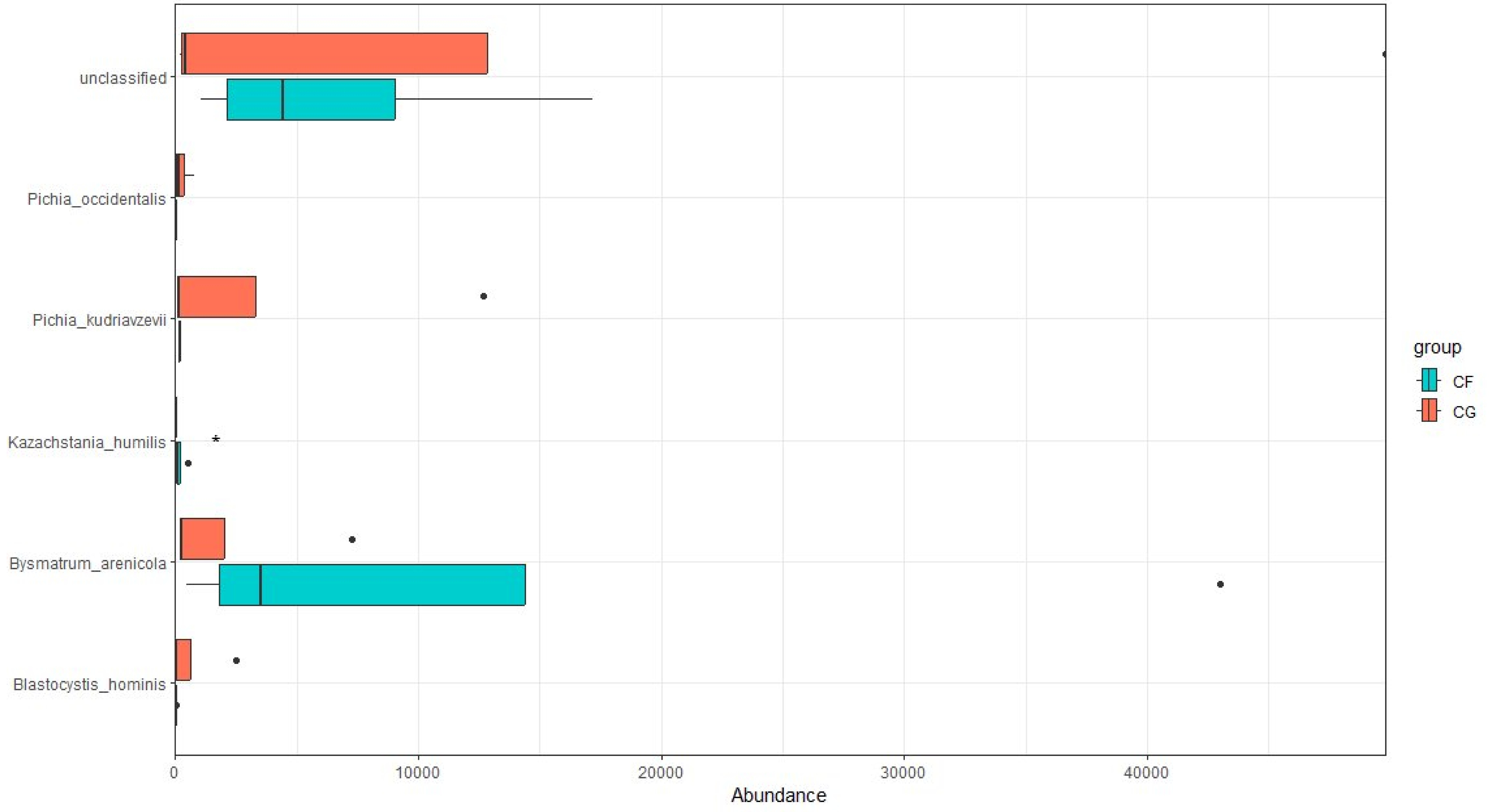

Figure S8. LDA abundance of Amplicon-based conventional primers: The plots (A), (B), (C) show Linear Discriminant Analysis (LDA) for (ITS1, ITS2, 18S) is utilized to distinguish groups based on the abundance of microbial species. The LDA results underscore particular microbial species that differentiate the groupings, including ITS1, ITS2, and 18S. The bar plots illustrate the LDA scores, highlighting species that markedly differentiate the groups, as evidenced by elevated LDA values. CG in red and CF in blue exhibit divergent patterns of enrichment for various species. An asterisk indicates species that are significantly differentially abundant.

(A) ITS1-IT2

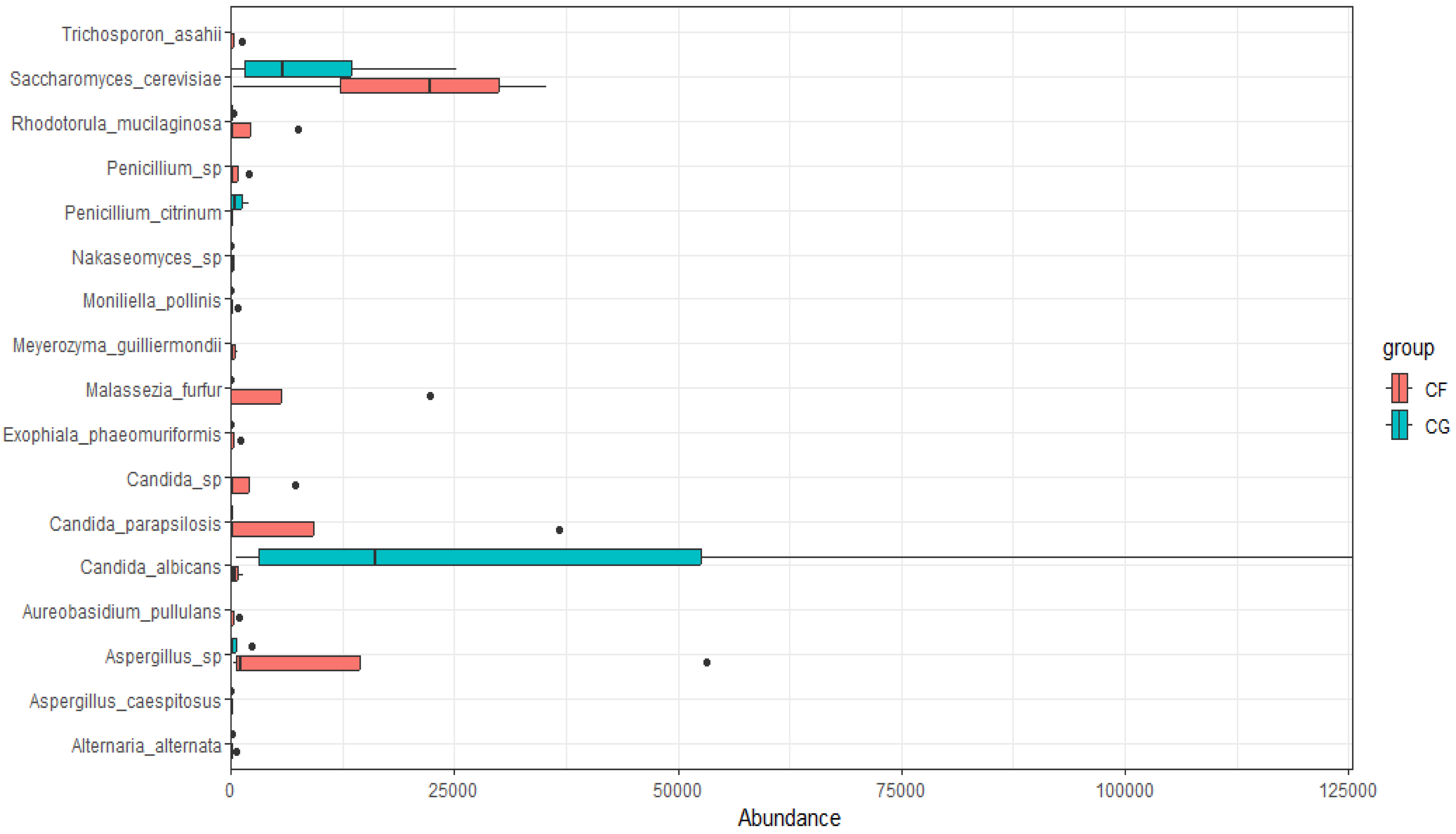

Figure S9. LDA abundance of combined primers: The plots (A), (B), (C) demonstrate Linear Discriminant Analysis (LDA) for combined datasets (ITS1-ITS2, ITS1-18S, ITS2-18S, ITS1-ITS2-18S). The plots illustrate the LDA scores, highlighting species that markedly differentiate the groups, as evidenced by elevated LDA values. CG in red and CF in blue exhibit divergent patterns of enrichment for various species. An asterisk indicates species that are significantly differentially abundant.

(B) ITS1-18S

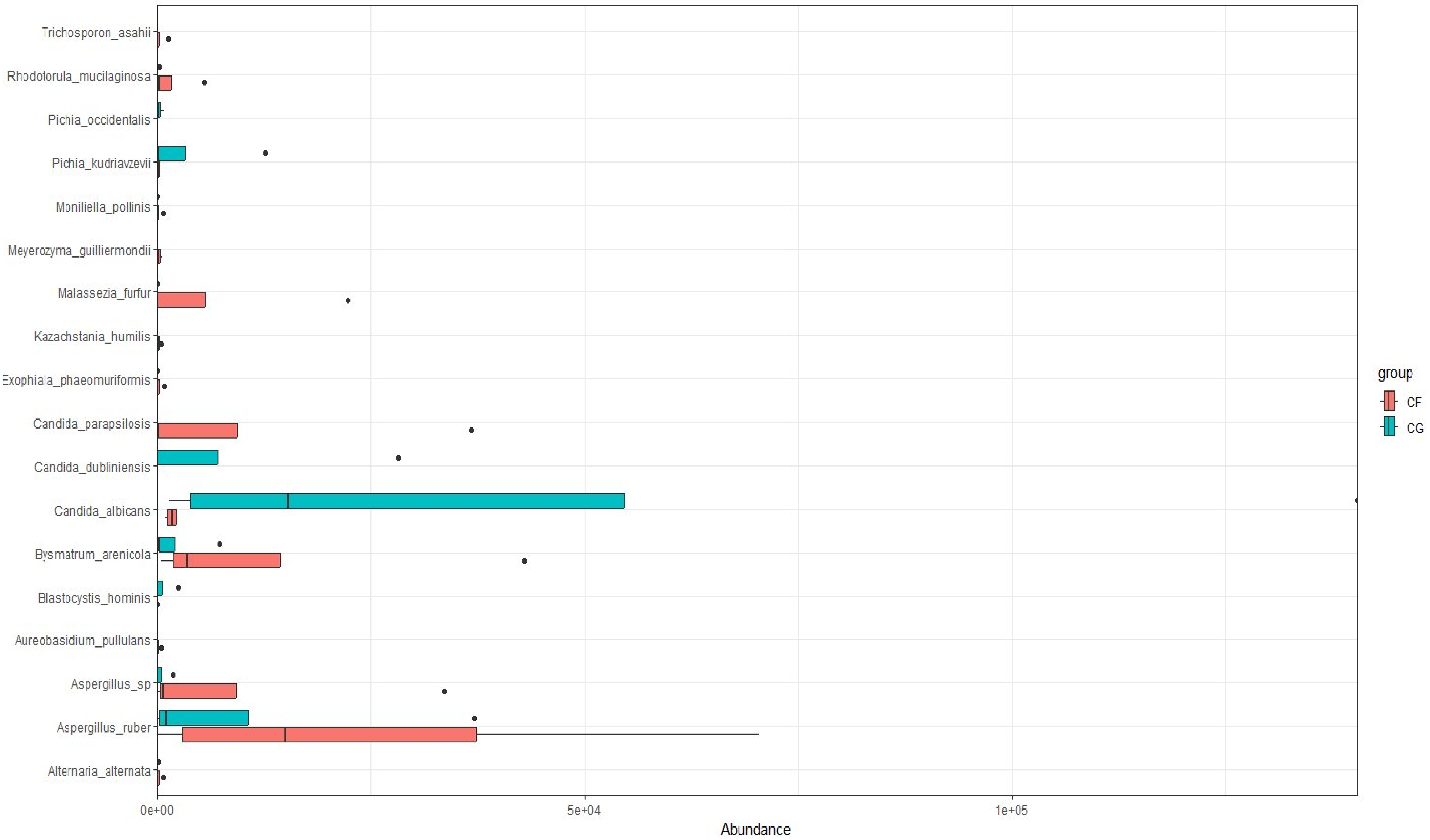

Figure S9. LDA abundance of combined primers: The plots (A), (B), (C) demonstrate Linear Discriminant Analysis (LDA) for combined datasets (ITS1-ITS2, ITS1-18S, ITS2-18S, ITS1-ITS2-18S). The plots illustrate the LDA scores, highlighting species that markedly differentiate the groups, as evidenced by elevated LDA values. CG in red and CF in blue exhibit divergent patterns of enrichment for various species. An asterisk indicates species that are significantly differentially abundant.

(C) ITS2-18S

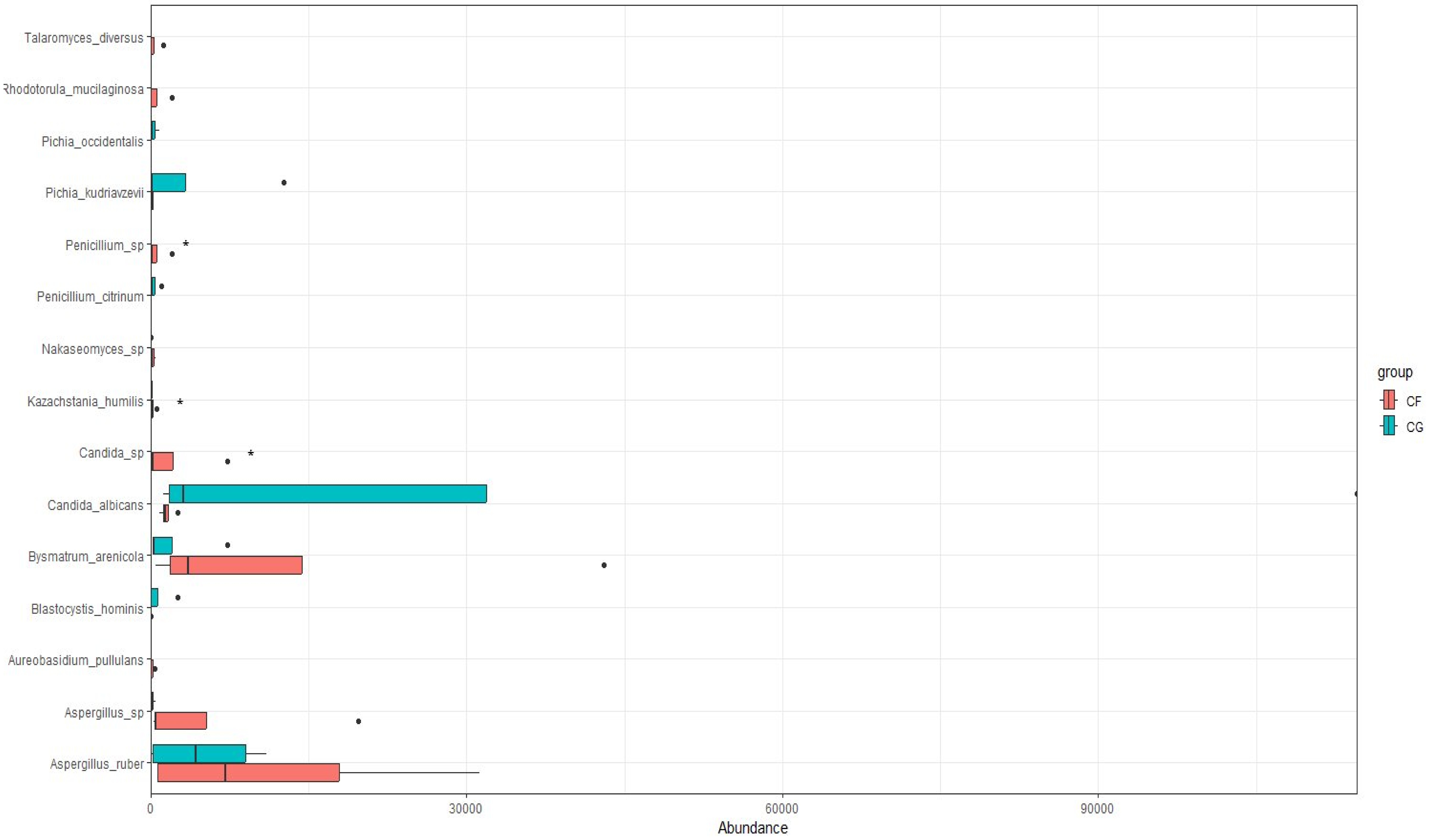

Figure S9. LDA abundance of combined primers: The plots (A), (B), (C) demonstrate Linear Discriminant Analysis (LDA) for combined datasets (ITS1-ITS2, ITS1-18S, ITS2-18S, ITS1-ITS2-18S). The plots illustrate the LDA scores, highlighting species that markedly differentiate the groups, as evidenced by elevated LDA values. CG in red and CF in blue exhibit divergent patterns of enrichment for various species. An asterisk indicates species that are significantly differentially abundant.

(D) ITS1-IT2-18S

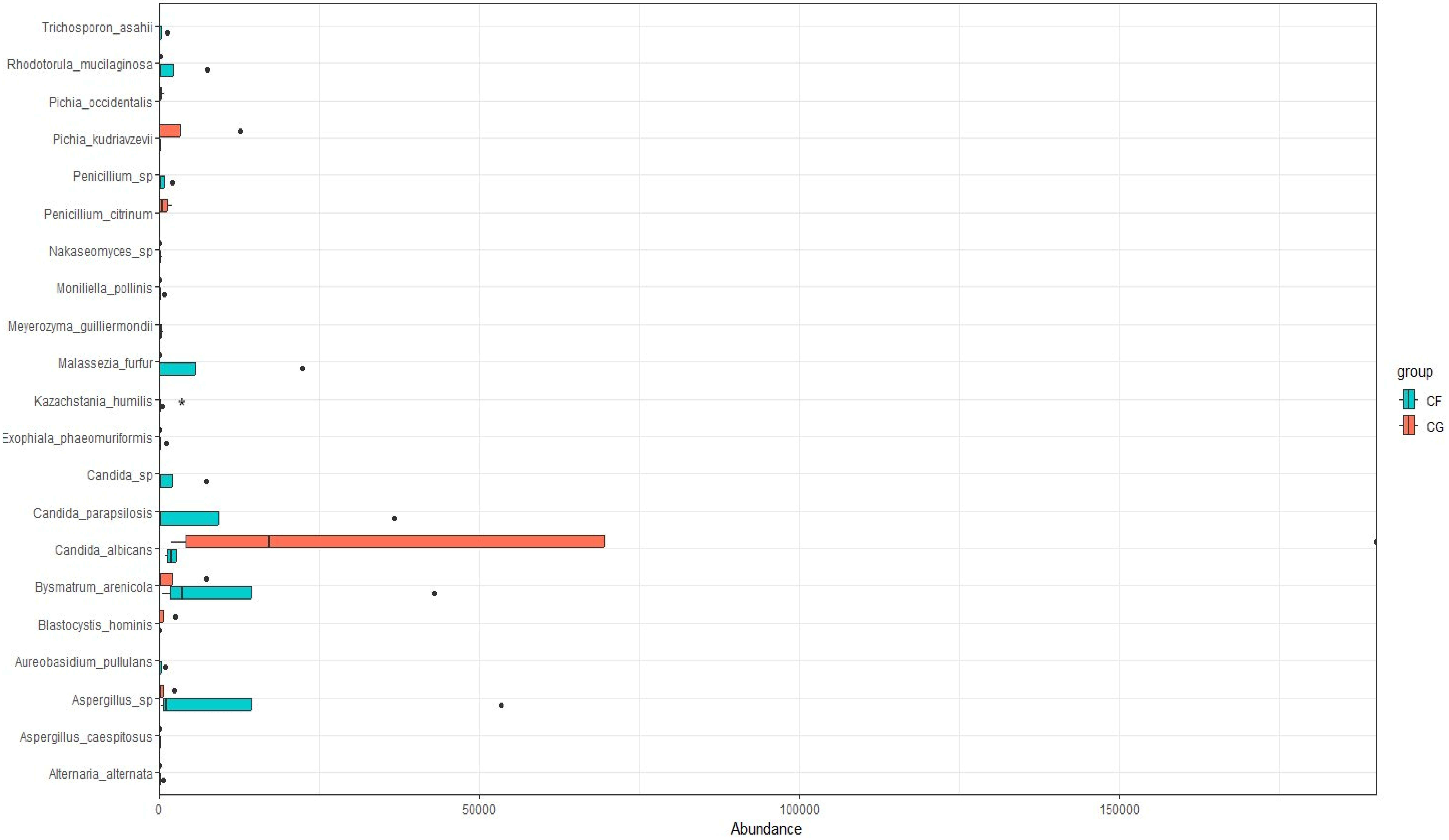

Figure S9. LDA abundance of combined primers: The plots (A), (B), (C) demonstrate Linear Discriminant Analysis (LDA) for combined datasets (ITS1-ITS2, ITS1-18S, ITS2-18S, ITS1-ITS2-18S). The plots illustrate the LDA scores, highlighting species that markedly differentiate the groups, as evidenced by elevated LDA values. CG in red and CF in blue exhibit divergent patterns of enrichment for various species. An asterisk indicates species that are significantly differentially abundant.

(A)

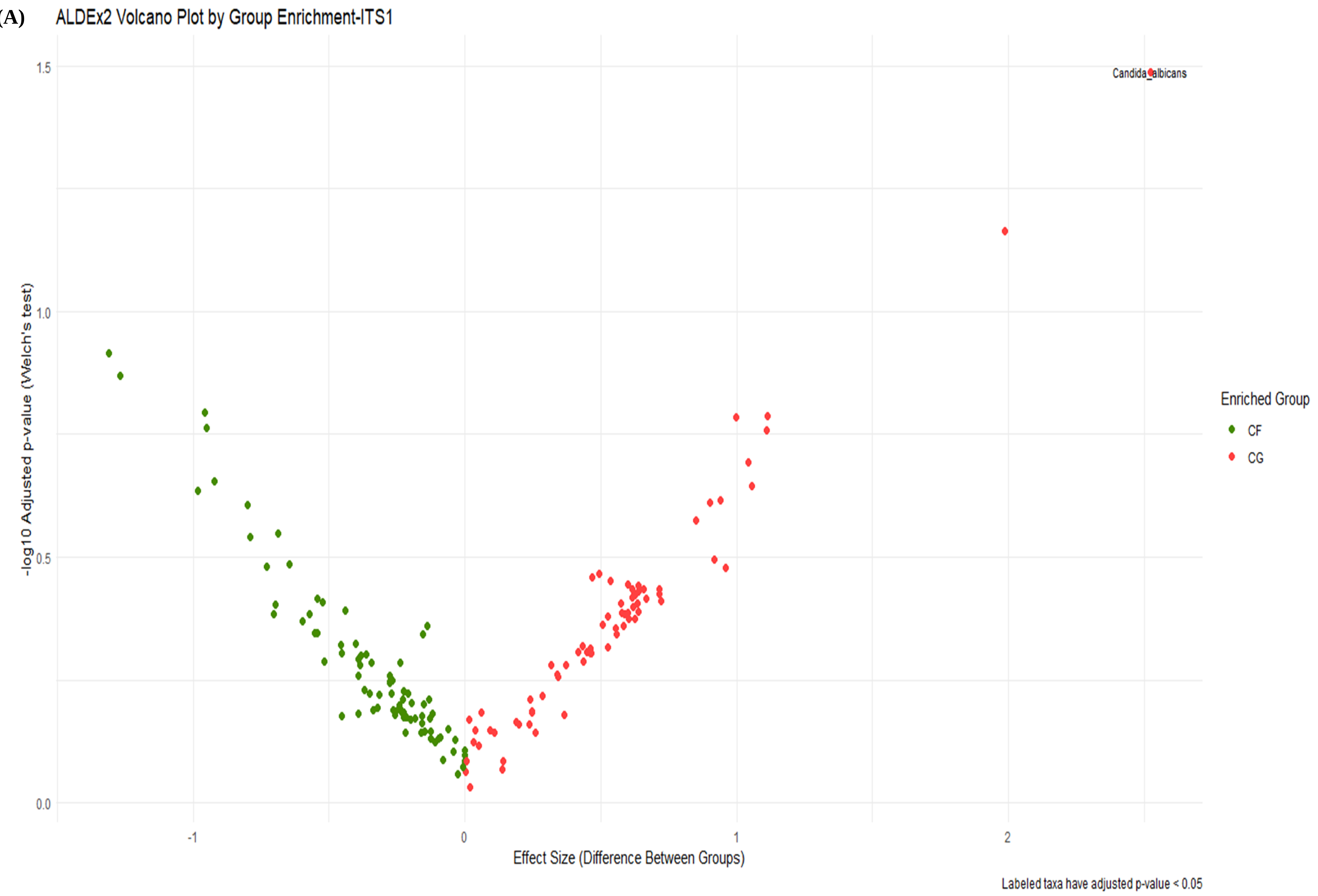

−log10(p-value)	p-value
0.2	0.63
0.4	0.40
0.6	0.25
0.8	0.16
1.0	0.10
1.2	0.063
1.5	0.032

Figure S10: ALDEx2 Volcano Plot of Differential Abundance Across singular datasets. The volcano plots (A), (B), (C) visualize the differential abundance of microbial taxa between cancer-free (CF, green) and cancer (CG, red) groups using ALDEx2 analysis for each primer set. The x-axis represents the effect size (difference in abundance between groups), while the y-axis shows the statistical significance ($-\log_{10}$ adjusted p-value). Taxa farther from the origin and higher on the plot are both highly differentially abundant and statistically significant. The plot emphasizes taxa that are enriched in each group, aiding in the identification of biologically meaningful and robust microbial biomarkers.

(B)

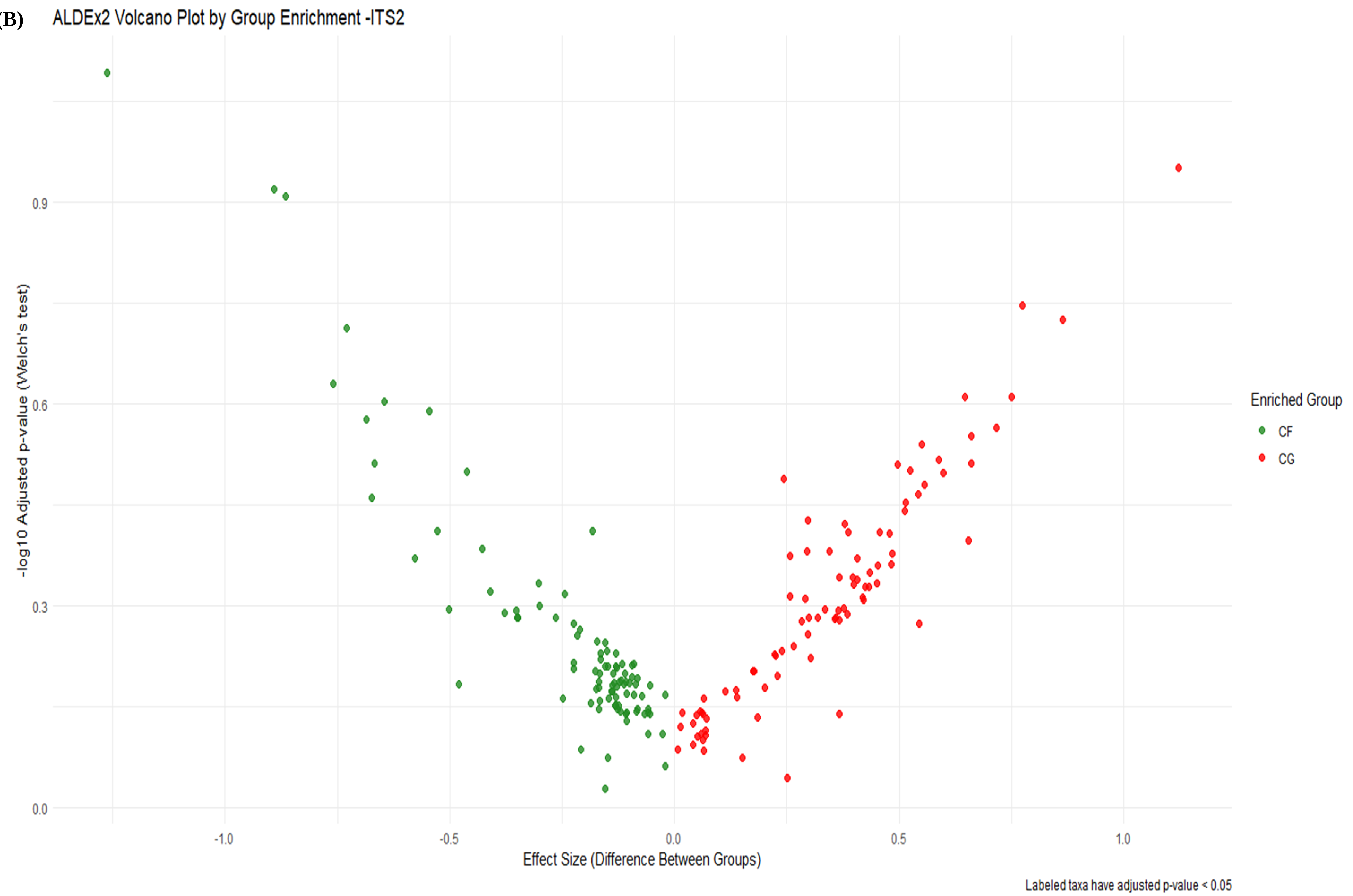

−log10(p-value)	p-value
0.2	0.63
0.4	0.40
0.6	0.25
0.8	0.16
1.0	0.10
1.2	0.063
1.5	0.032

Figure S10: ALDEx2 Volcano Plot of Differential Abundance Across singular datasets. The volcano plots (A), (B), (C) visualize the differential abundance of microbial taxa between cancer-free (CF, green) and cancer (CG, red) groups using ALDEx2 analysis for each primer set. The x-axis represents the effect size (difference in abundance between groups), while the y-axis shows the statistical significance ($-\log_{10}$ adjusted p-value). Taxa farther from the origin and higher on the plot are both highly differentially abundant and statistically significant. The plot emphasizes taxa that are enriched in each group, aiding in the identification of biologically meaningful and robust microbial biomarkers.

(C)

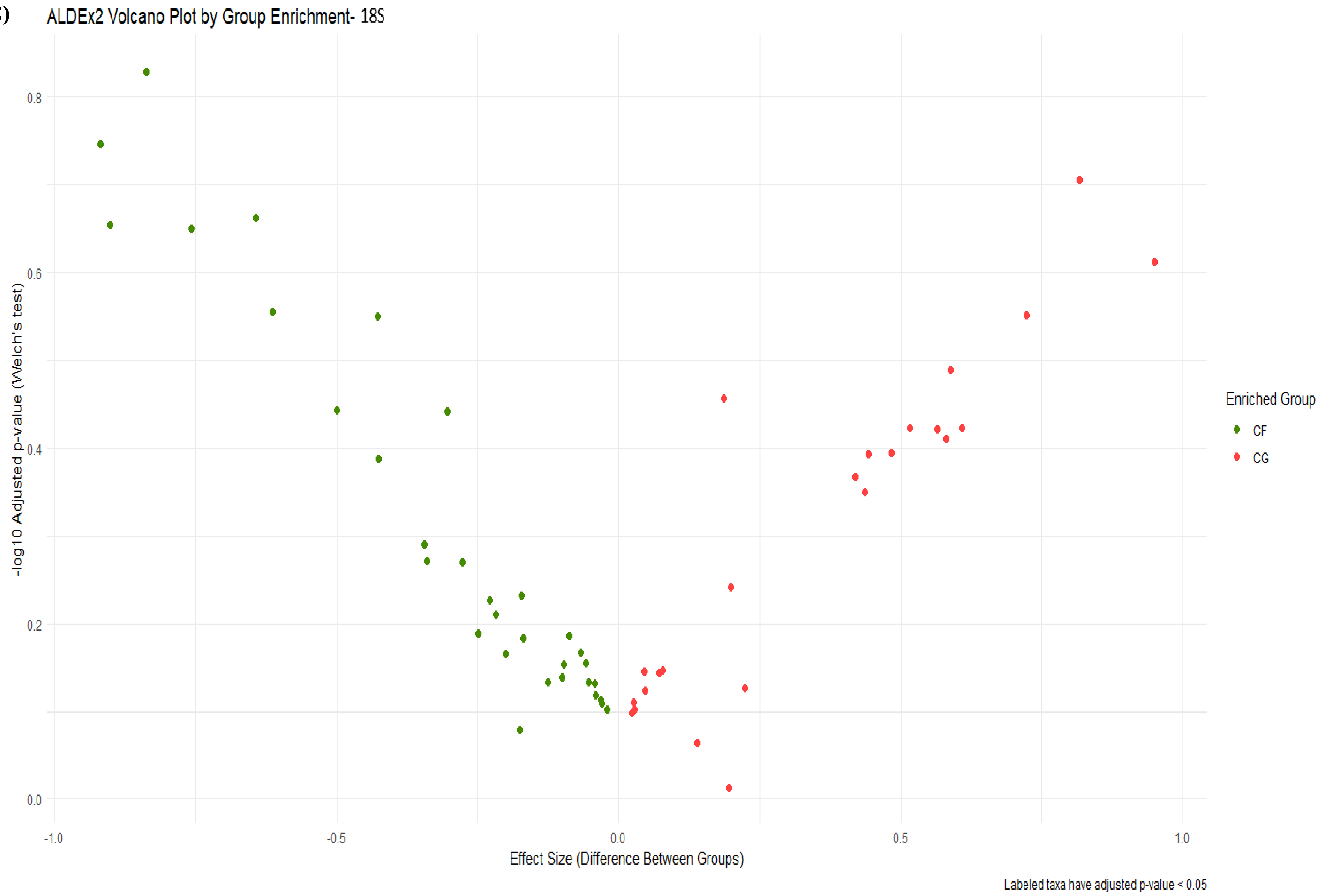

−log10(p-value)	p-value
0.2	0.63
0.4	0.40
0.6	0.25
0.8	0.16
1.0	0.10
1.2	0.063
1.5	0.032

Figure S10: ALDEx2 Volcano Plot of Differential Abundance Across singular datasets. The volcano plots (A), (B), (C) visualize the differential abundance of microbial taxa between cancer-free (CF, green) and cancer (CG, red) groups using ALDEx2 analysis for each primer set. The x-axis represents the effect size (difference in abundance between groups), while the y-axis shows the statistical significance ($-\log_{10}$ adjusted p-value). Taxa farther from the origin and higher on the plot are both highly differentially abundant and statistically significant. The plot emphasizes taxa that are enriched in each group, aiding in the identification of biologically meaningful and robust microbial biomarkers.

(A)

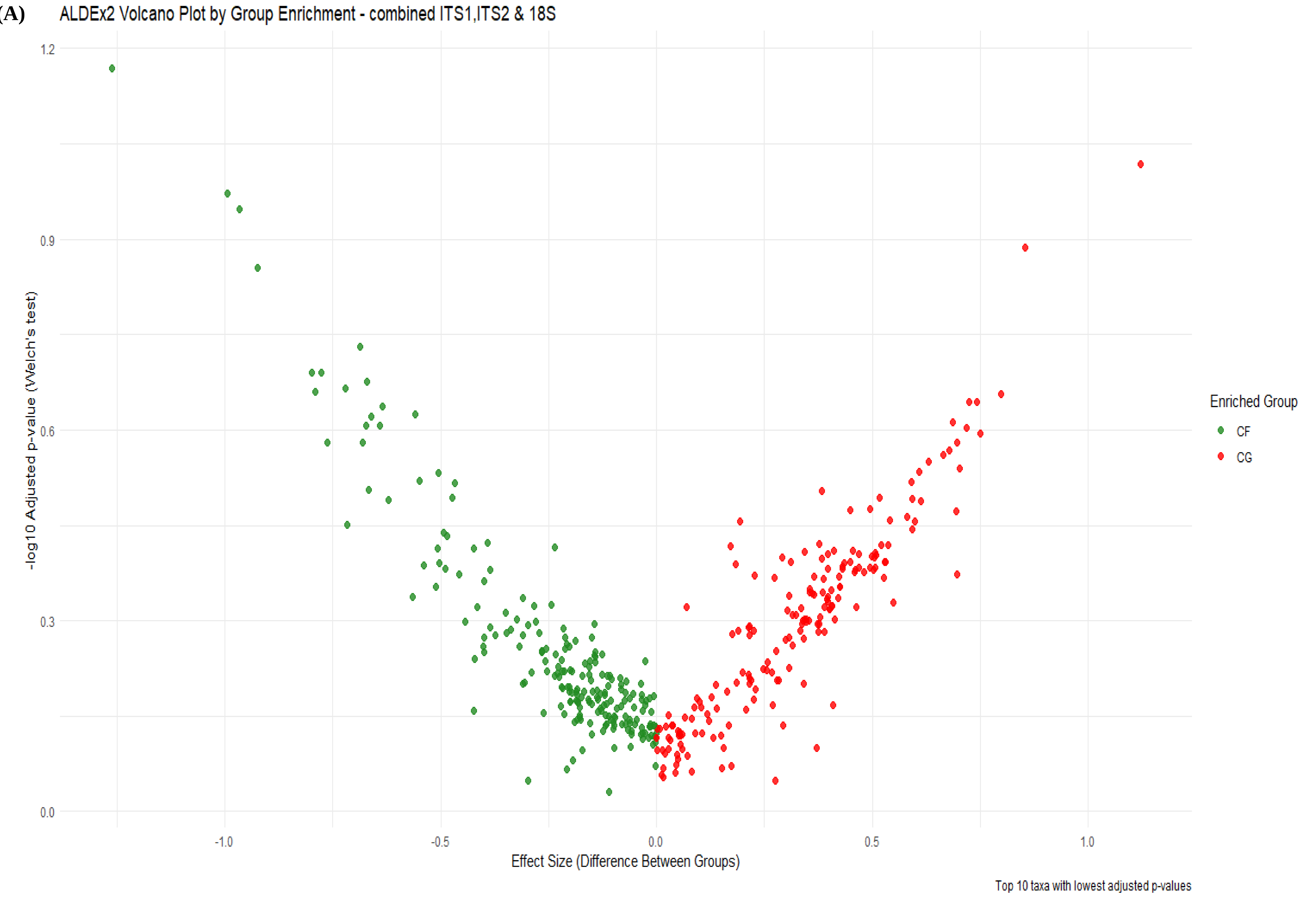

−log10(p-value)	p-value
0.2	0.63
0.4	0.40
0.6	0.25
0.8	0.16
1.0	0.10
1.2	0.063
1.5	0.032

Figure S11: ALDEx2 Volcano Plot of Differential Abundance Across Combined Primer Sets. The volcano plots (A), (B), (C) visualizes the differential abundance of microbial taxa between cancer-free (CF, green) and cancer (CG, red) groups using ALDEx2 analysis. The x-axis represents the effect size (difference in abundance between groups), while the y-axis shows the statistical significance ($-\log_{10}$ adjusted p-value). Taxa farther from the origin and higher on the plot are both highly differentially abundant and statistically significant. The plot emphasizes taxa that are enriched in each group, aiding in the identification of biologically meaningful and robust microbial biomarkers.

(B)

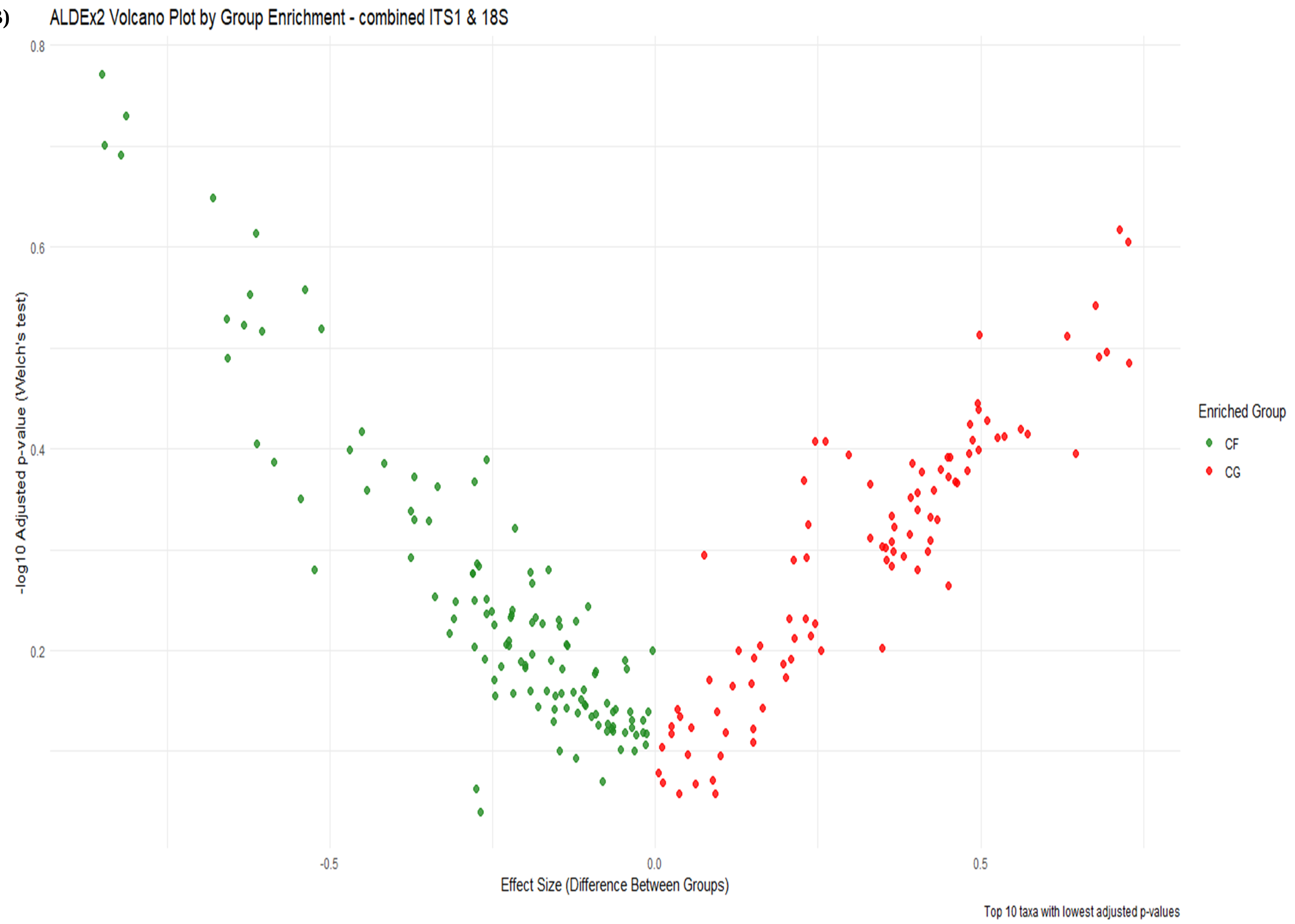

−log10(p-value)	p-value
0.2	0.63
0.4	0.40
0.6	0.25
0.8	0.16
1.0	0.10
1.2	0.063
1.5	0.032

Figure S11: ALDEx2 Volcano Plot of Differential Abundance Across Combined Primer Sets. The volcano plots (A), (B), (C) visualizes the differential abundance of microbial taxa between cancer-free (CF, green) and cancer (CG, red) groups using ALDEx2 analysis. The x-axis represents the effect size (difference in abundance between groups), while the y-axis shows the statistical significance ($-\log_{10}$ adjusted p-value). Taxa farther from the origin and higher on the plot are both highly differentially abundant and statistically significant. The plot emphasizes taxa that are enriched in each group, aiding in the identification of biologically meaningful and robust microbial biomarkers.

(C)

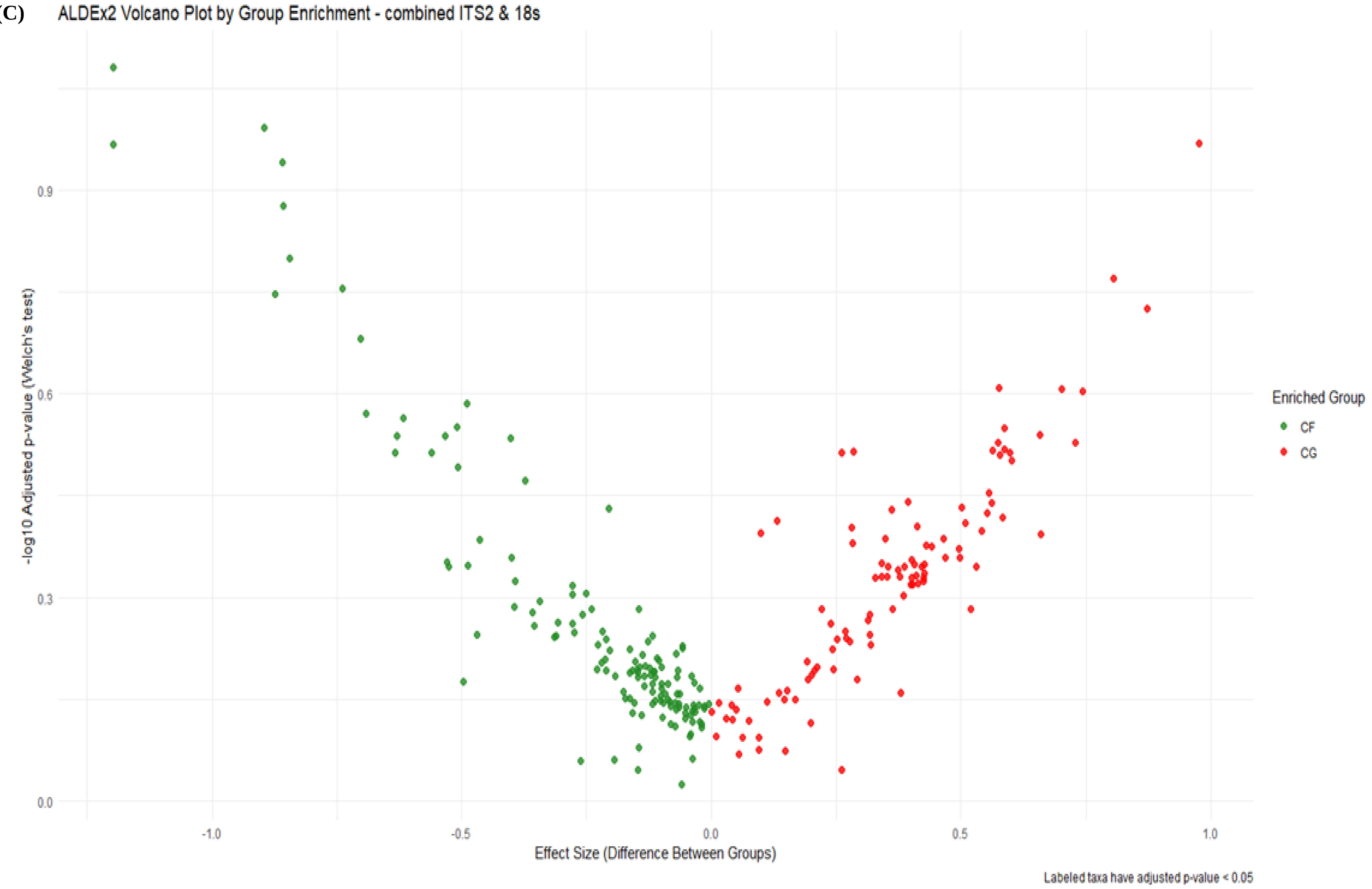

−log10(p-value)	p-value
0.2	0.63
0.4	0.40
0.6	0.25
0.8	0.16
1.0	0.10
1.2	0.063
1.5	0.032

Figure S11: ALDEx2 Volcano Plot of Differential Abundance Across Combined Primer Sets. The volcano plots (A), (B), (C) visualizes the differential abundance of microbial taxa between cancer-free (CF, green) and cancer (CG, red) groups using ALDEx2 analysis. The x-axis represents the effect size (difference in abundance between groups), while the y-axis shows the statistical significance ($-\log_{10}$ adjusted p-value). Taxa farther from the origin and higher on the plot are both highly differentially abundant and statistically significant. The plot emphasizes taxa that are enriched in each group, aiding in the identification of biologically meaningful and robust microbial biomarkers.

(A) ITS1

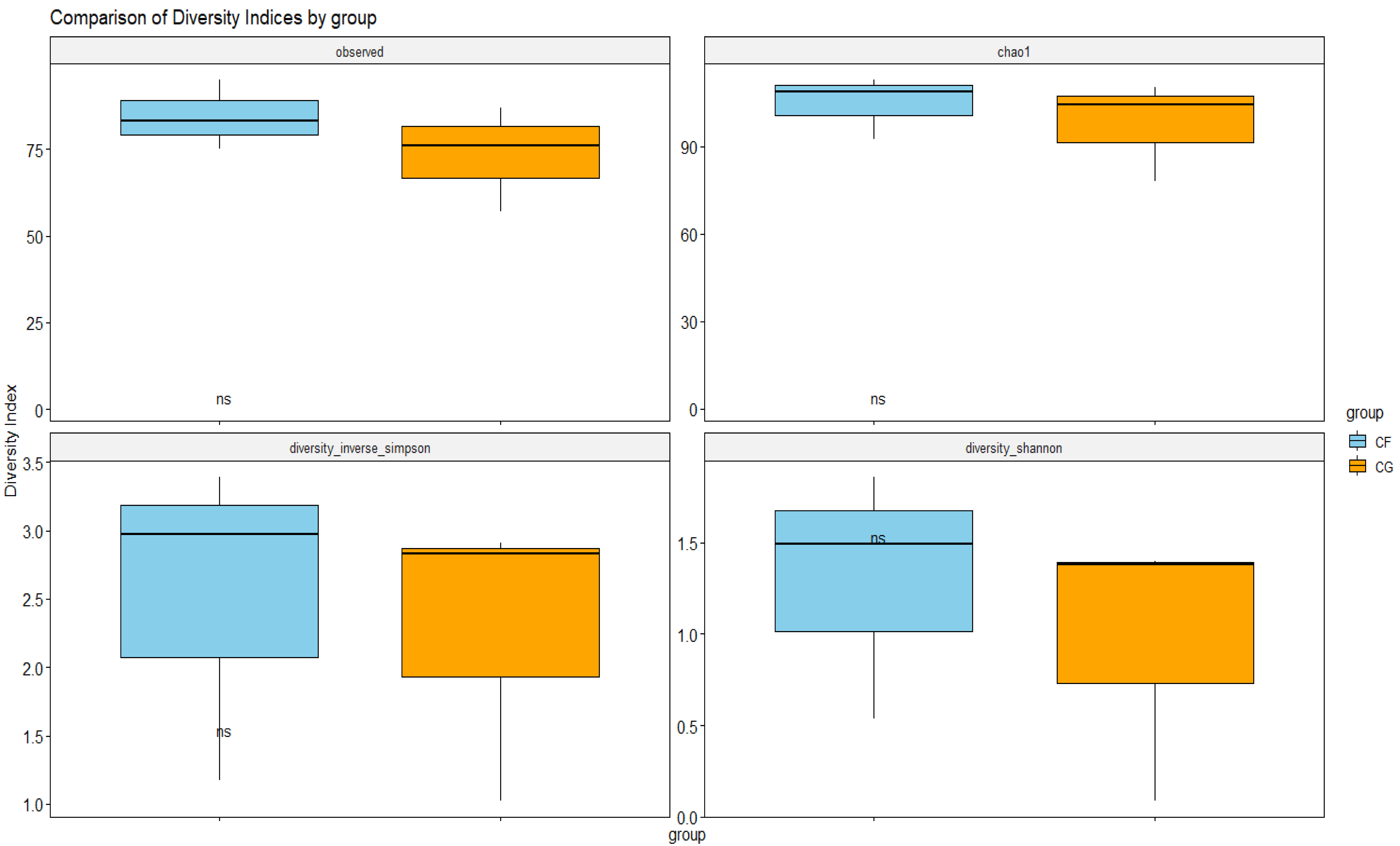

Figure S12. Alpha diversity: The (A), (B), (C) box plots illustrate the comparison of diversity indices between the cancer-free and cancer groups for the three primer sets (ITS1, ITS2, and 18S). They compare the diversity indices (observed richness, Chao1, inverse Simpson, and Shannon) for ITS1, ITS2, and 18S. The diversity indices for 18S indicate variations among groups richness, in which CF exhibiting greater diversity. Variation in diversity richness for ITS primers are higher than 18S especially ITS2, indicating that certain populations possess more diverse microbial communities than others with ITS2 primer set. Yet no varied levels of statistical significance (p-values $\leq$0.05).

(B) ITS2

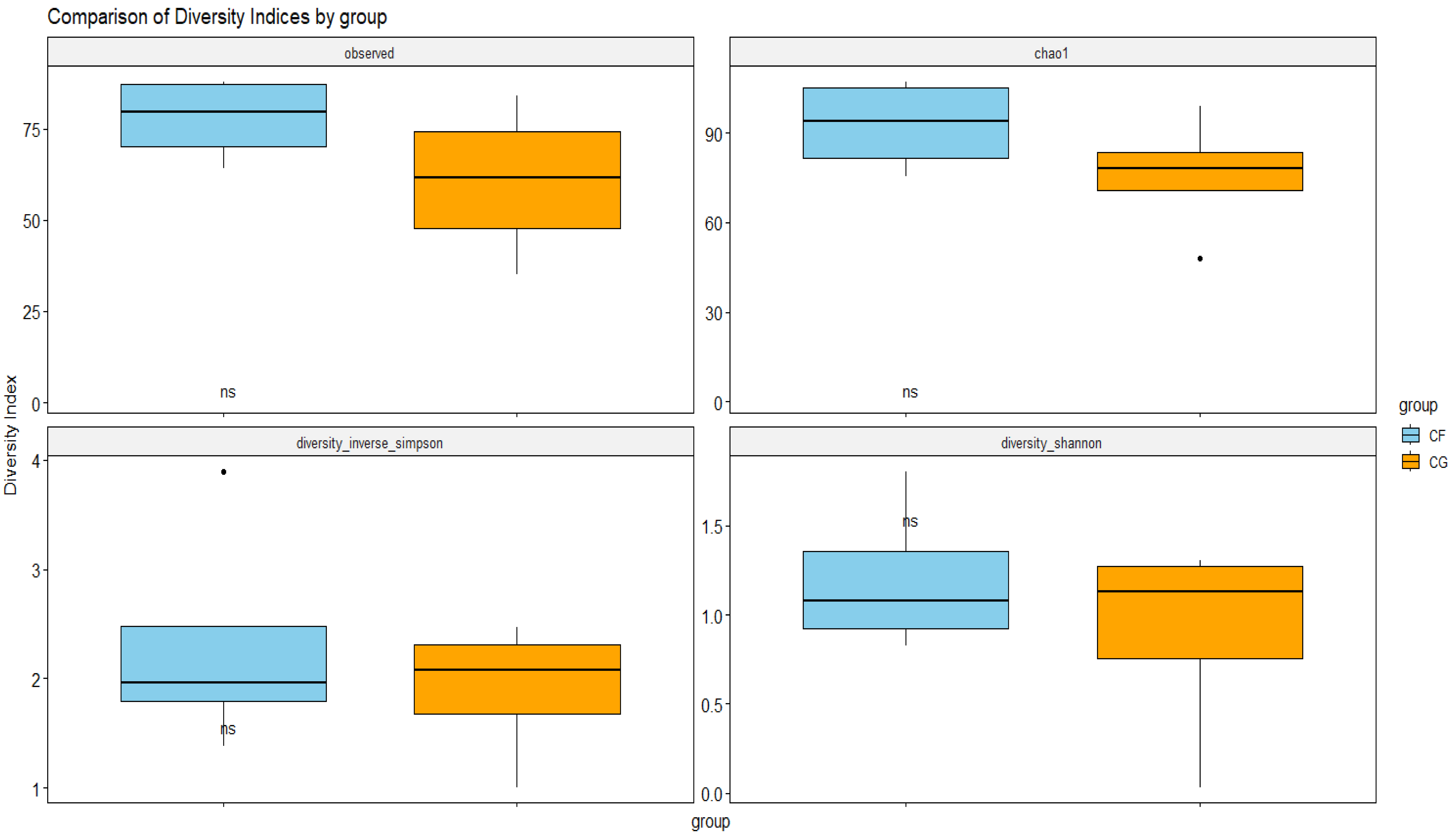

Figure S12. Alpha diversity: The box plots (A), (B), (C) illustrate the comparison of diversity indices between the cancer-free and cancer groups for the three primer sets (ITS1, ITS2, and 18S). They compare the diversity indices (observed richness, Chao1, inverse Simpson, and Shannon) for ITS1, ITS2, and 18S. The diversity indices for 18S indicate variations among groups richness, in which CF exhibiting greater diversity. Variation in diversity richness for ITS primers are higher than 18S especially ITS2, indicating that certain populations possess more diverse microbial communities than others with ITS2 primer set. Yet no varied levels of statistical significance (p-values $\leq$0.05).

(C) 18S

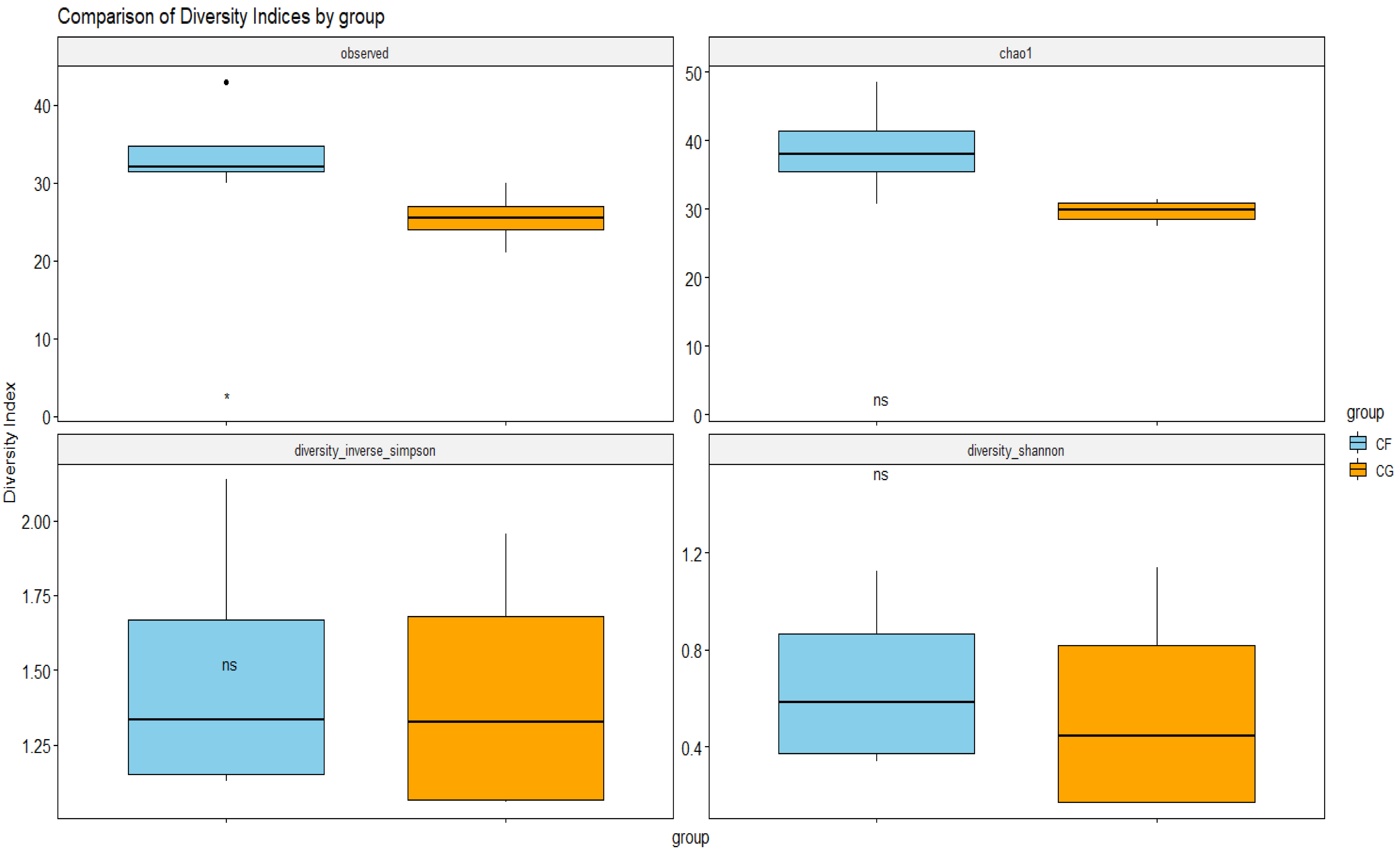

Figure S12. Alpha diversity: The box plots (A), (B), (C) illustrate the comparison of diversity indices between the cancer-free and cancer groups for the three primer sets (ITS1, ITS2, and 18S). They compare the diversity indices (observed richness, Chao1, inverse Simpson, and Shannon) for ITS1, ITS2, and 18S. The diversity indices for 18S indicate variations among groups richness, in which CF exhibiting greater diversity. Variation in diversity richness for ITS primers are higher than 18S especially ITS2, indicating that certain populations possess more diverse microbial communities than others with ITS2 primer set. Yet no varied levels of statistical significance (p-values ≤ 0.05).

(A) ITS1

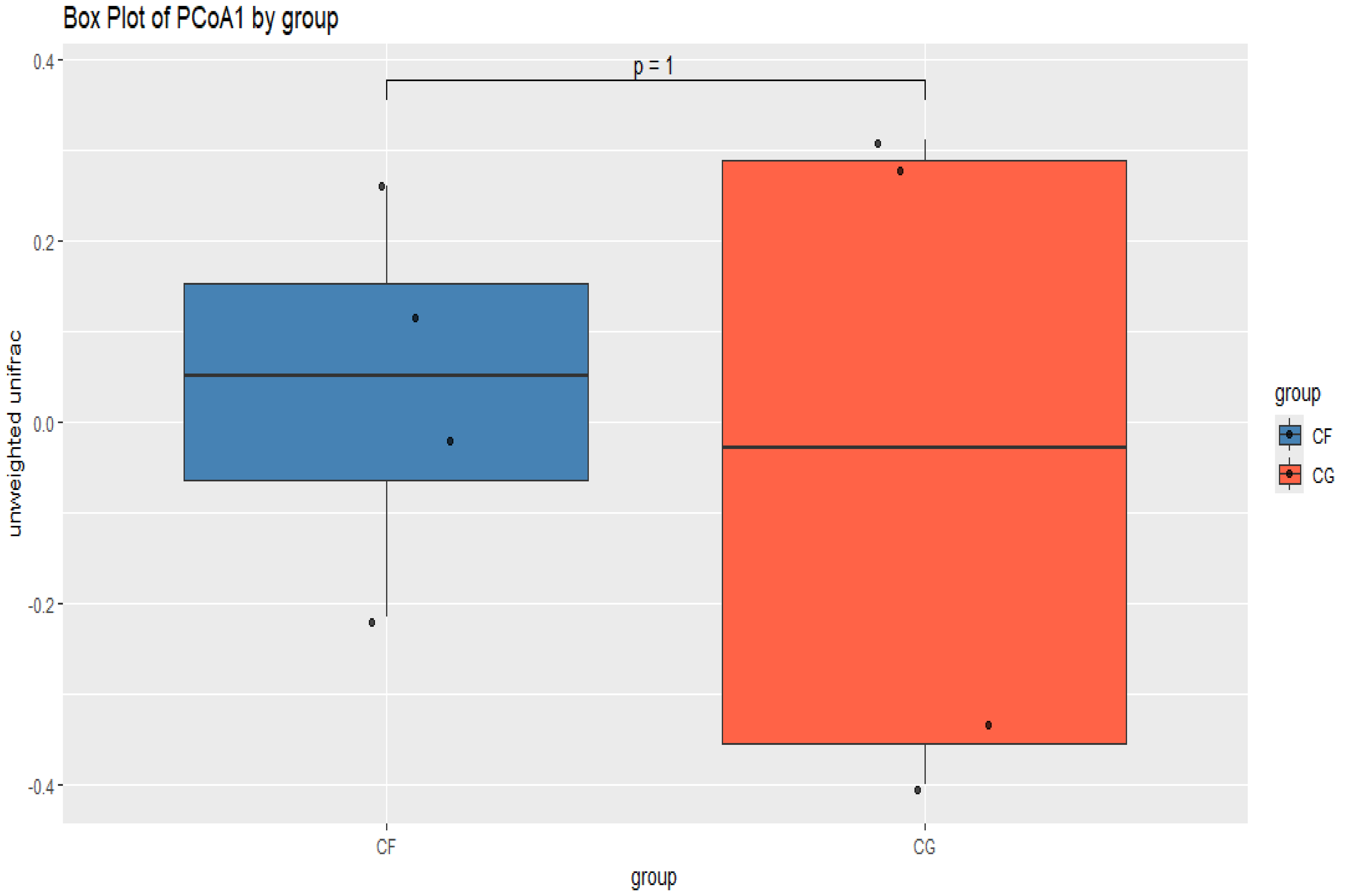

(C) 18S

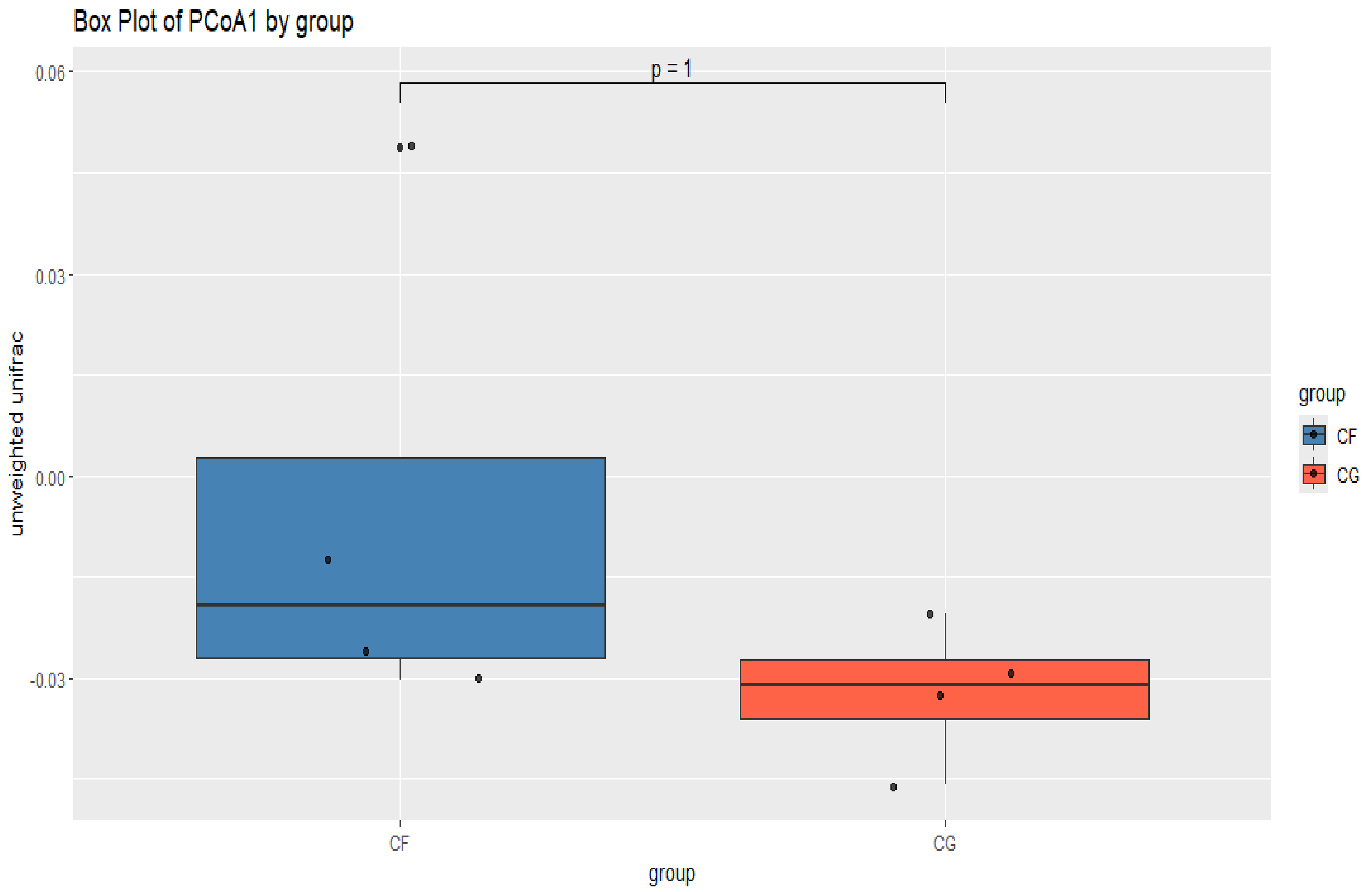

(B) ITS2

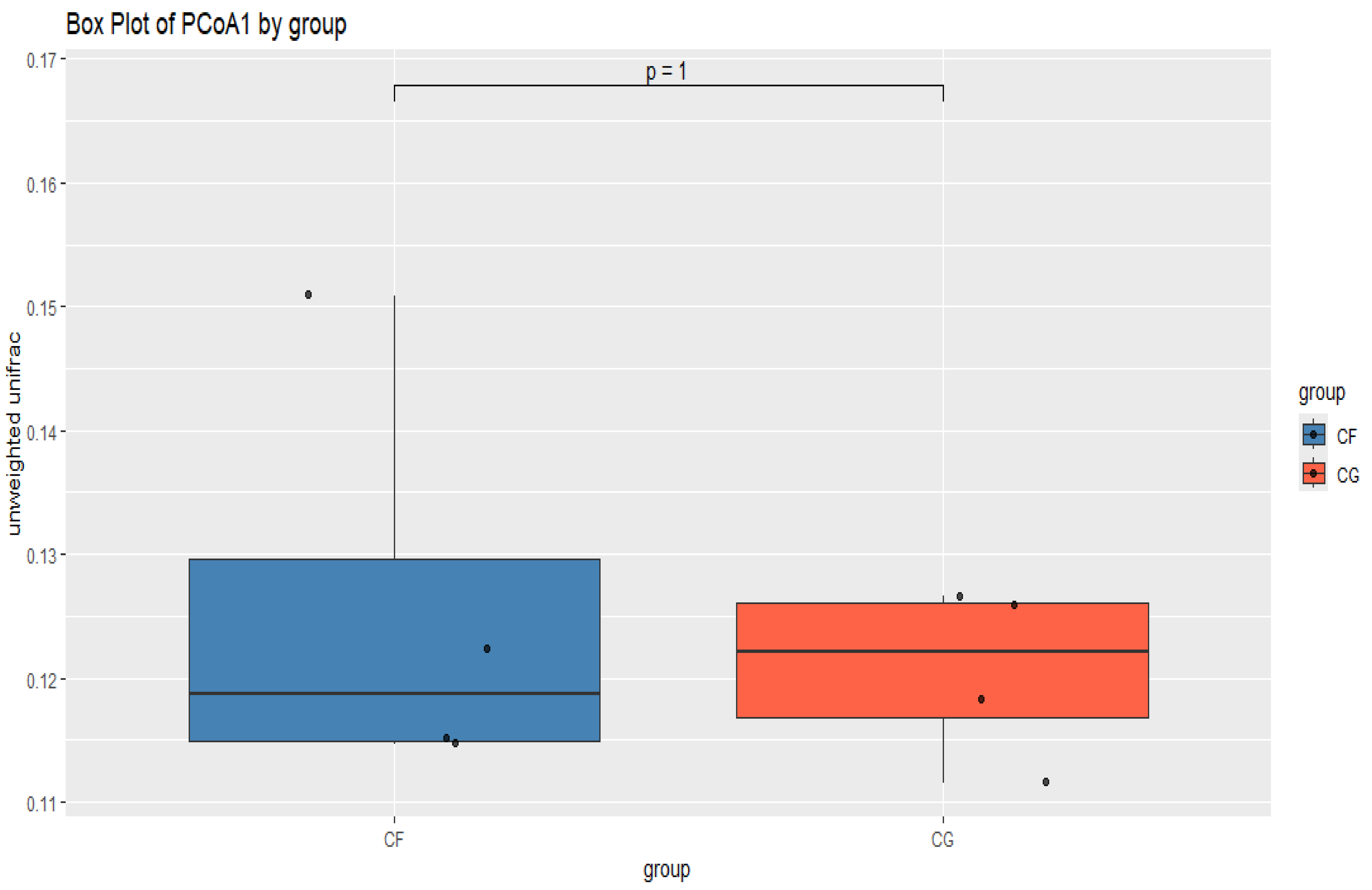

Figure S13. Beta Diversity: the box plots (A), (B), (C) illustrate the beta diversity evaluated by PCoA, for each of the three primer sets. ITS1: The unweighted UniFrac distances of CF and CG groups are similar. The box plot shows overlap, indicating that both groups are similar in diversity. 18S: The CF and CG groups have similar microbial communities. The two groups' unweighted UniFrac distances are identical along this axis. ITS2: Box plots indicating no diversity difference. The figures show that CF and CG groups had similar microbial diversity. Despite modest visible changes due to insignificant variability, demonstrating that the microbial communities are statistically equivalent across the three unweighted UniFrac distance components.

(A) Combined ITS1-ITS2

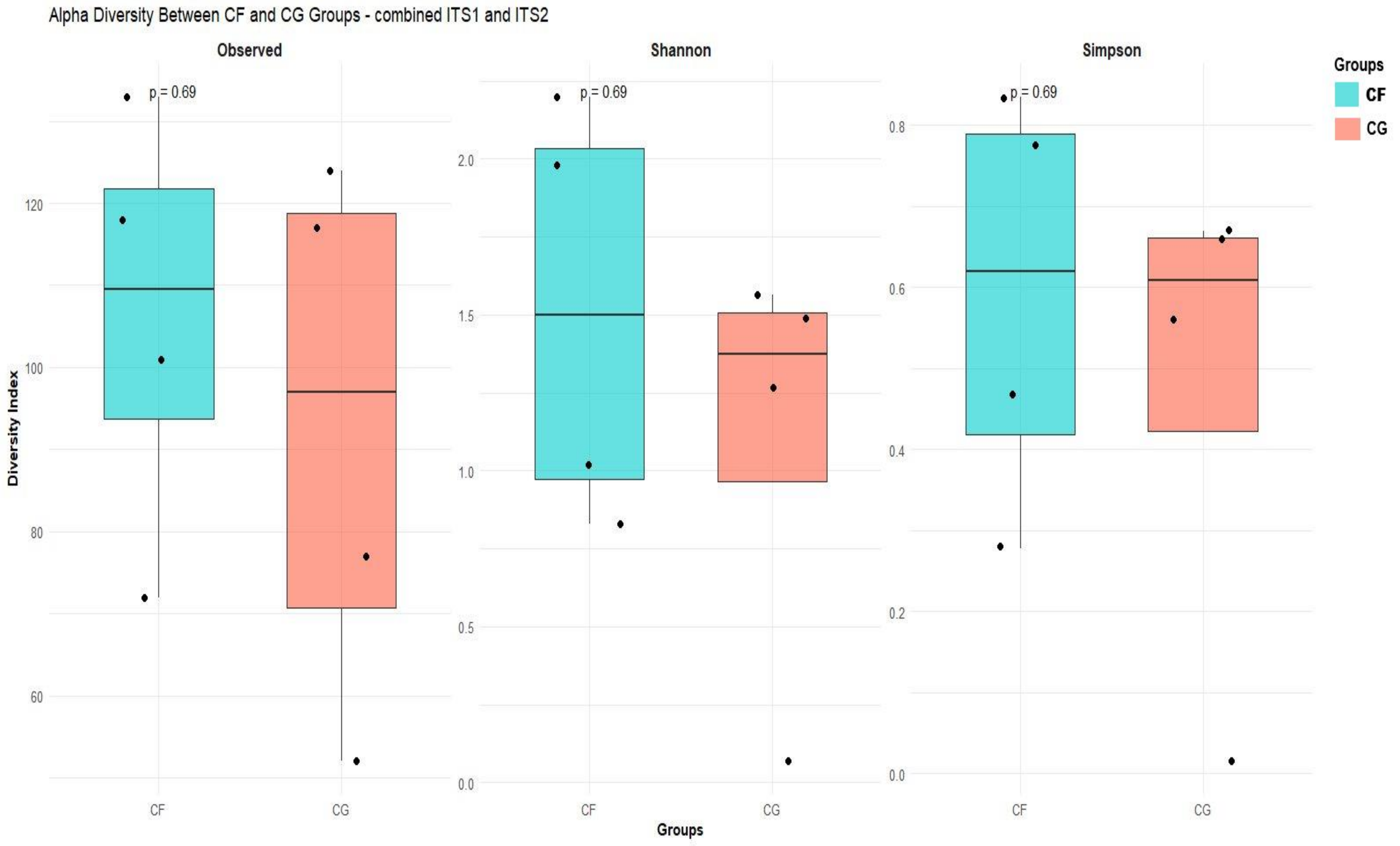

Figure S14. Alpha diversity comparisons between cancer-free (CF) and cancer (CG) groups using combined primer datasets (18S, ITS1, ITS2, and their combinations). Boxplots (A), (B), (C) represent Observed Richness, Shannon Index, and Simpson Index across groups. The 18S-ITS1-ITS2 combination revealed the strongest trend of reduced diversity in CG, particularly in the Simpson index ($p = 0.09$). The ITS1-ITS2 combination showed consistent diversity reductions across indices, while ITS1-18S and ITS2-18S produced weaker or mixed signals, likely due to 18S-associated taxonomic noise.

(B) Combined ITS1-ITS2-18S

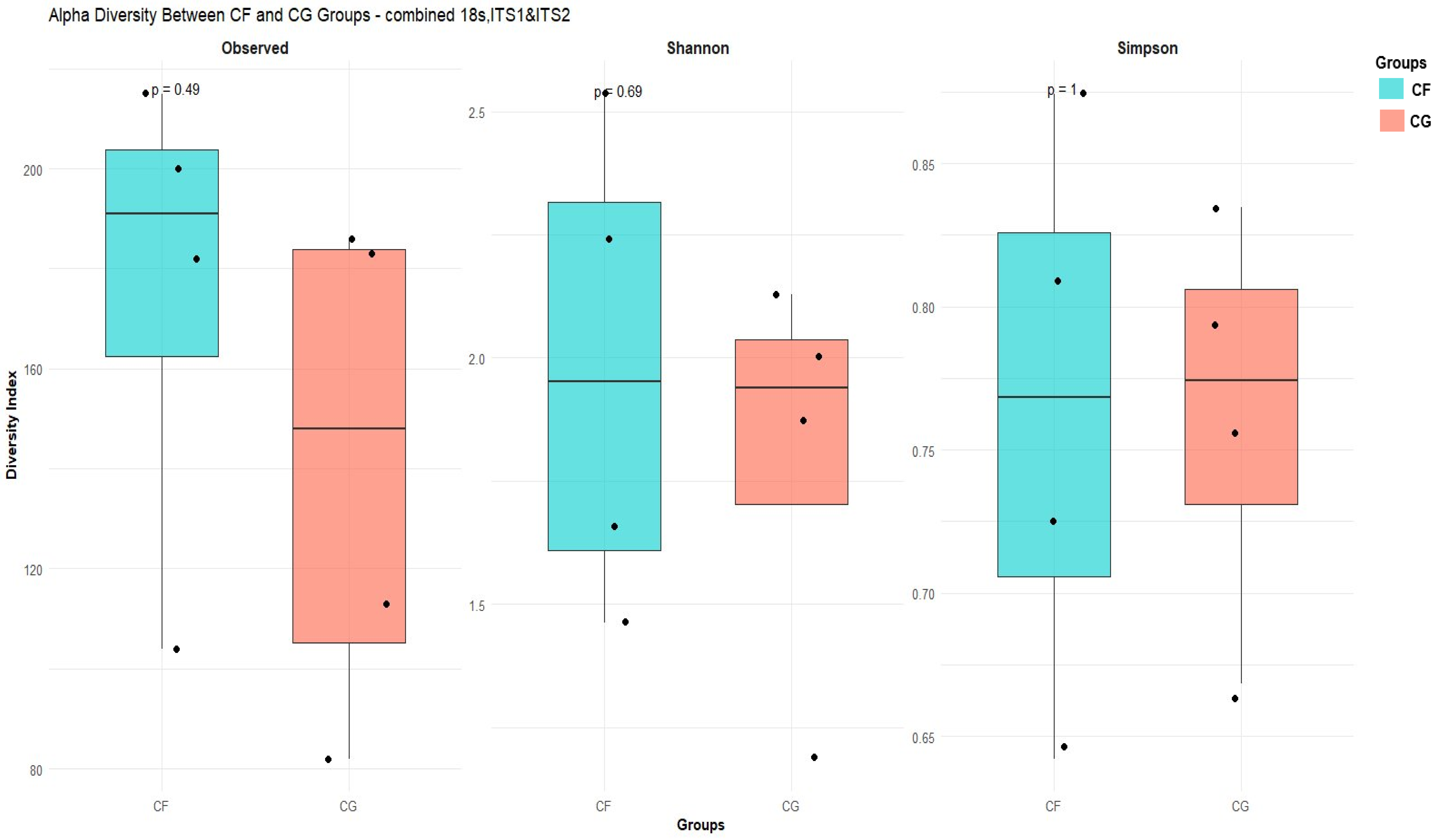

Figure S14. Alpha diversity comparisons between cancer-free (CF) and cancer (CG) groups using combined primer datasets (18S, ITS1, ITS2, and their combinations). Boxplots (A), (B), (C) represent Observed Richness, Shannon Index, and Simpson Index across groups. The 18S-ITS1-ITS2 combination revealed the strongest trend of reduced diversity in CG, particularly in the Simpson index (p = 0.09). The ITS1-ITS2 combination showed consistent diversity reductions across indices, while ITS1-18S and ITS2-18S produced weaker or mixed signals, likely due to 18S-associated taxonomic noise.

(C) Combined ITS1-18S

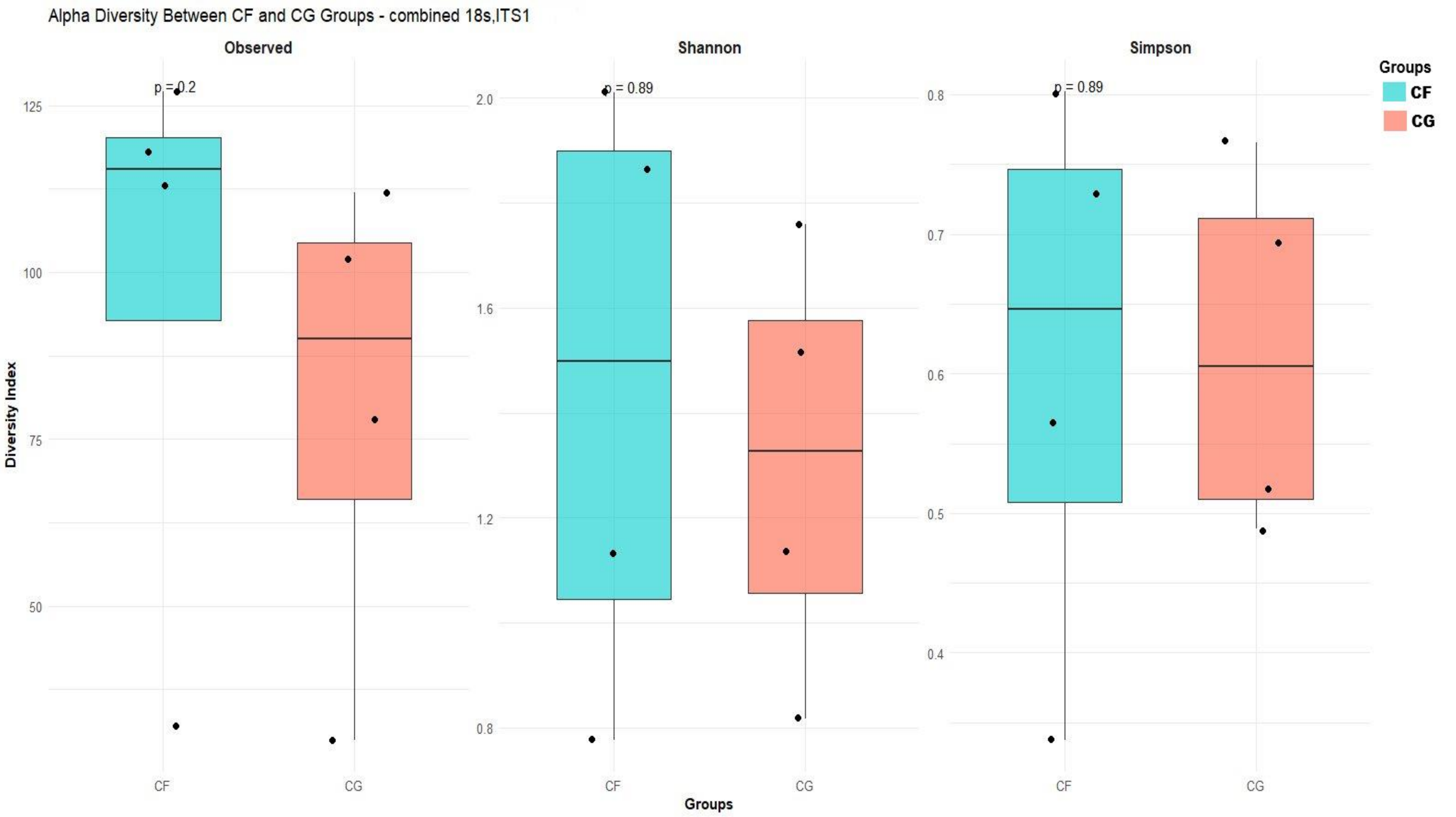

Figure S14. Alpha diversity comparisons between cancer-free (CF) and cancer (CG) groups using combined primer datasets (18S, ITS1, ITS2, and their combinations). Boxplots (A), (B), (C) represent Observed Richness, Shannon Index, and Simpson Index across groups. The 18S-ITS1-ITS2 combination revealed the strongest trend of reduced diversity in CG, particularly in the Simpson index ($p = 0.09$). The ITS1-ITS2 combination showed consistent diversity reductions across indices, while ITS1-18S and ITS2-18S produced weaker or mixed signals, likely due to 18S-associated taxonomic noise.

(D) Combined ITS2-18S

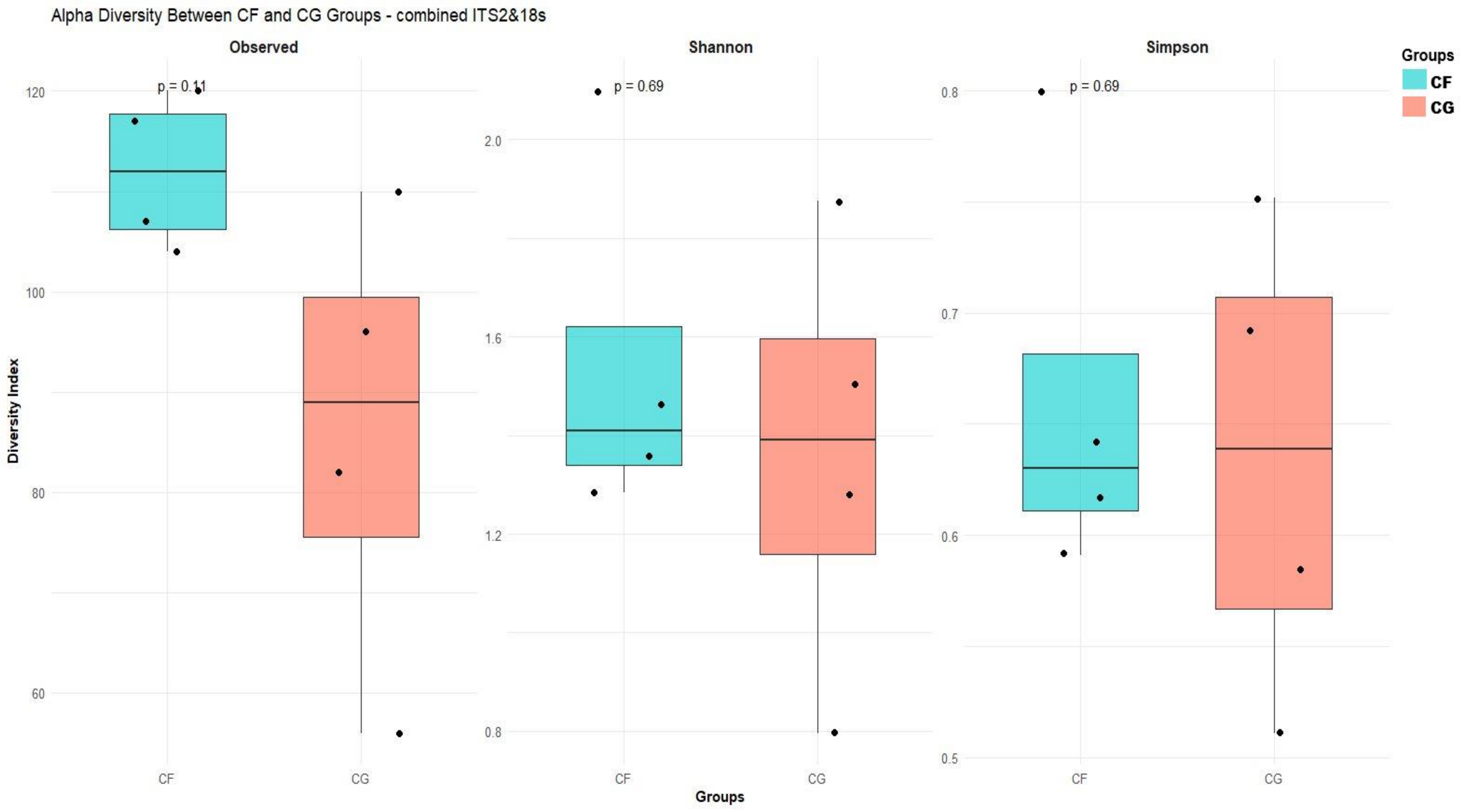

Figure S14. Alpha diversity comparisons between cancer-free (CF) and cancer (CG) groups using combined primer datasets (18S, ITS1, ITS2, and their combinations). Boxplots (A), (B), (C) represent Observed Richness, Shannon Index, and Simpson Index across groups. The 18S-ITS1-ITS2 combination revealed the strongest trend of reduced diversity in CG, particularly in the Simpson index ($p = 0.09$). The ITS1-ITS2 combination showed consistent diversity reductions across indices, while ITS1-18S and ITS2-18S produced weaker or mixed signals, likely due to 18S-associated taxonomic noise.

Table S1: Comparative alpha diversity metrics across primer combinations

Primer Combination	Observed Richness	Shannon Index	Simpson Index	Overall Trend
**18S - ITS1 - ITS2**	CF > CG (p = 0.22); clear trend of reduced richness in CG.	CF > CG (p = 0.69); more even distribution in CF.	CF > CG (p = 0.09); suggests reduced dominance in CG.	**Strongest diversity signal**; Simpson nearly significant.
**ITS1 - ITS2**	CF > CG (p = 0.69); moderate difference.	CF > CG (p = 0.69); lower evenness in CG.	CF > CG (p = 0.09); possible diversity loss in CG.	**Consistent pattern**, especially in Simpson.
**ITS1 - 18S**	Minimal difference (p = 0.69); overlapping boxes.	Slightly higher in CF (p = 0.69); weak resolution.	Nearly identical (p = 0.69); no group distinction.	**Least discriminatory** combination.
**ITS2 - 18S**	Slight richness drop in CG (p = 0.14); trend visible.	Near identical diversity (p = 0.69); overlapping distributions.	Slight increase in CG (p = 0.69); contradicts richness trend.	**Inconclusive**; mixed signals between indices, possibly due to 18S amplification.

Comparative alpha diversity metrics across primer combinations. The table highlights variations in taxonomic resolution and group enrichment trends across different primer combinations of datasets (ITS1, ITS2, 18S). Observed index refer to the rare species richness, Shannon index measures richness, and Simpson index evaluates evenness (CF = cancer free participants, CG = cancer group, p =p-value)

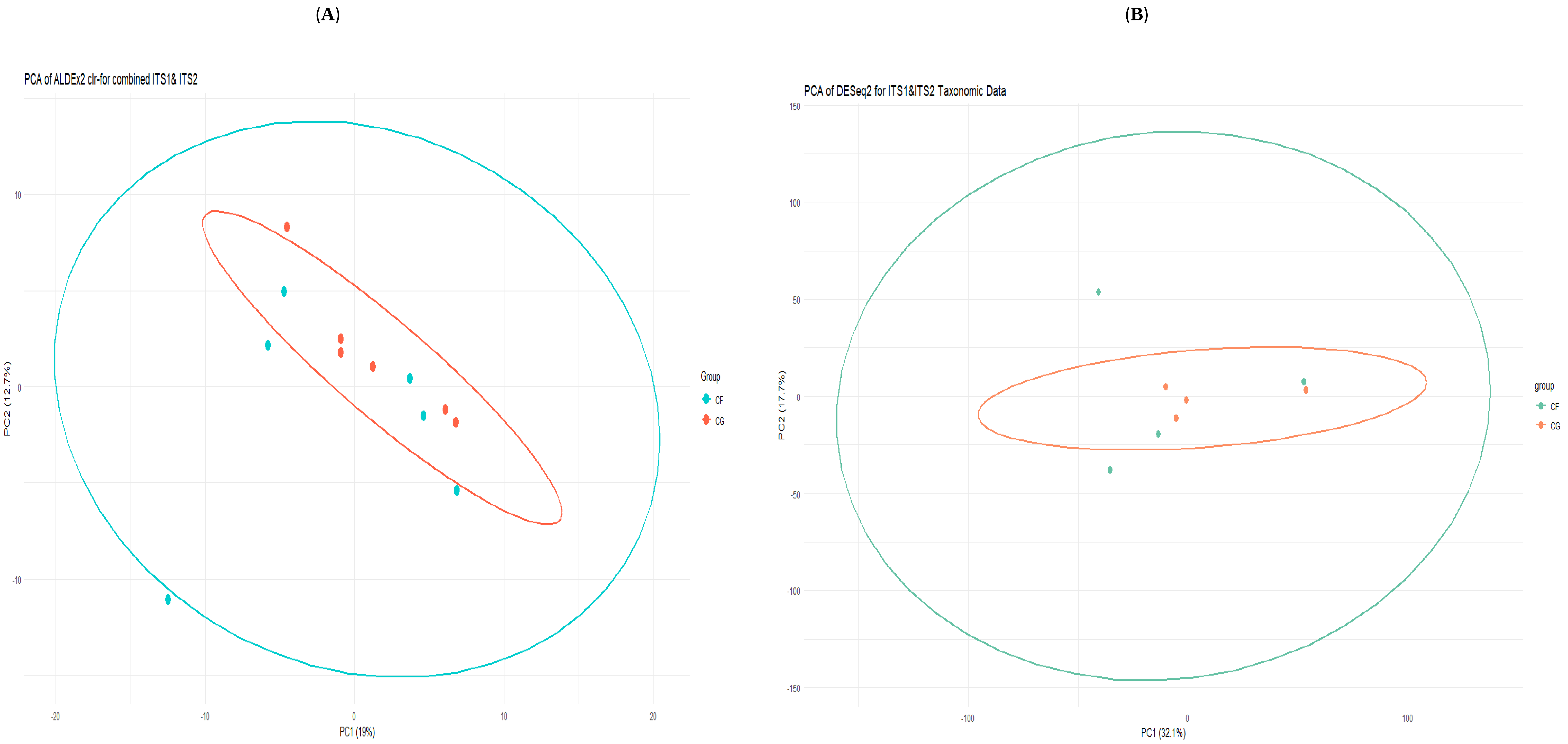

Figure 15: Principal Component Analysis (PCA) plots of combined datasets. Generated by ALDEx2 (*A*) and DESeq2 (*B*), illustrating the variance and clustering patterns among samples based on the first two principal components (PC1 and PC2), explained variance percentages. The plots' ellipses represent the group dispersion, visualizing group overlap or separation to highlight the differences or similarities in fungal community composition.

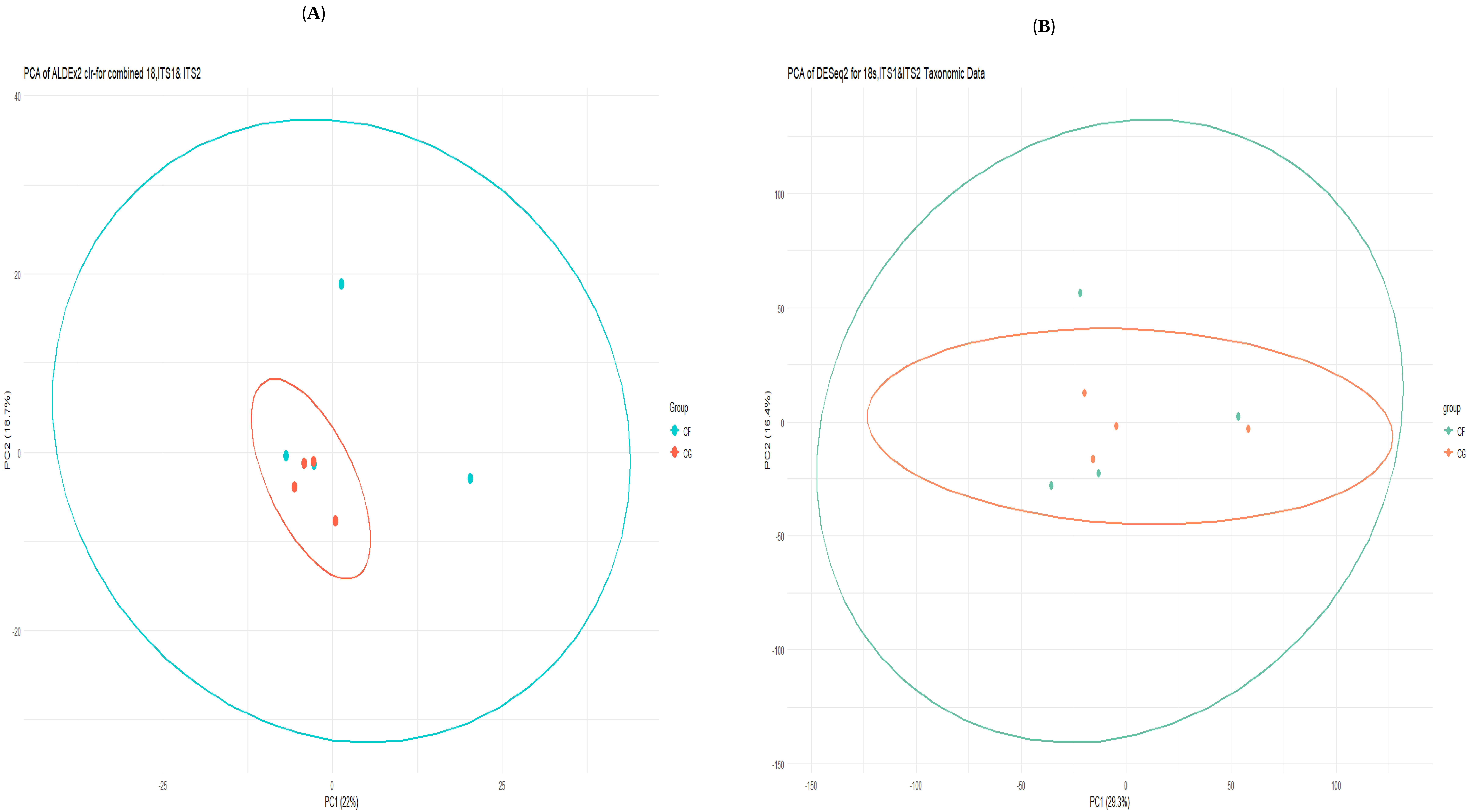

Figure 16: Principal Component Analysis (PCA) plots of combined datasets. Generated by ALDEx2 (*A*) and DESeq2 (*B*), illustrating the variance and clustering patterns among samples based on the first two principal components (PC1 and PC2), explained variance percentages. The plots' ellipses represent the group dispersion, visualizing group overlap or separation to highlight the differences or similarities in fungal community composition.

(A) (B)

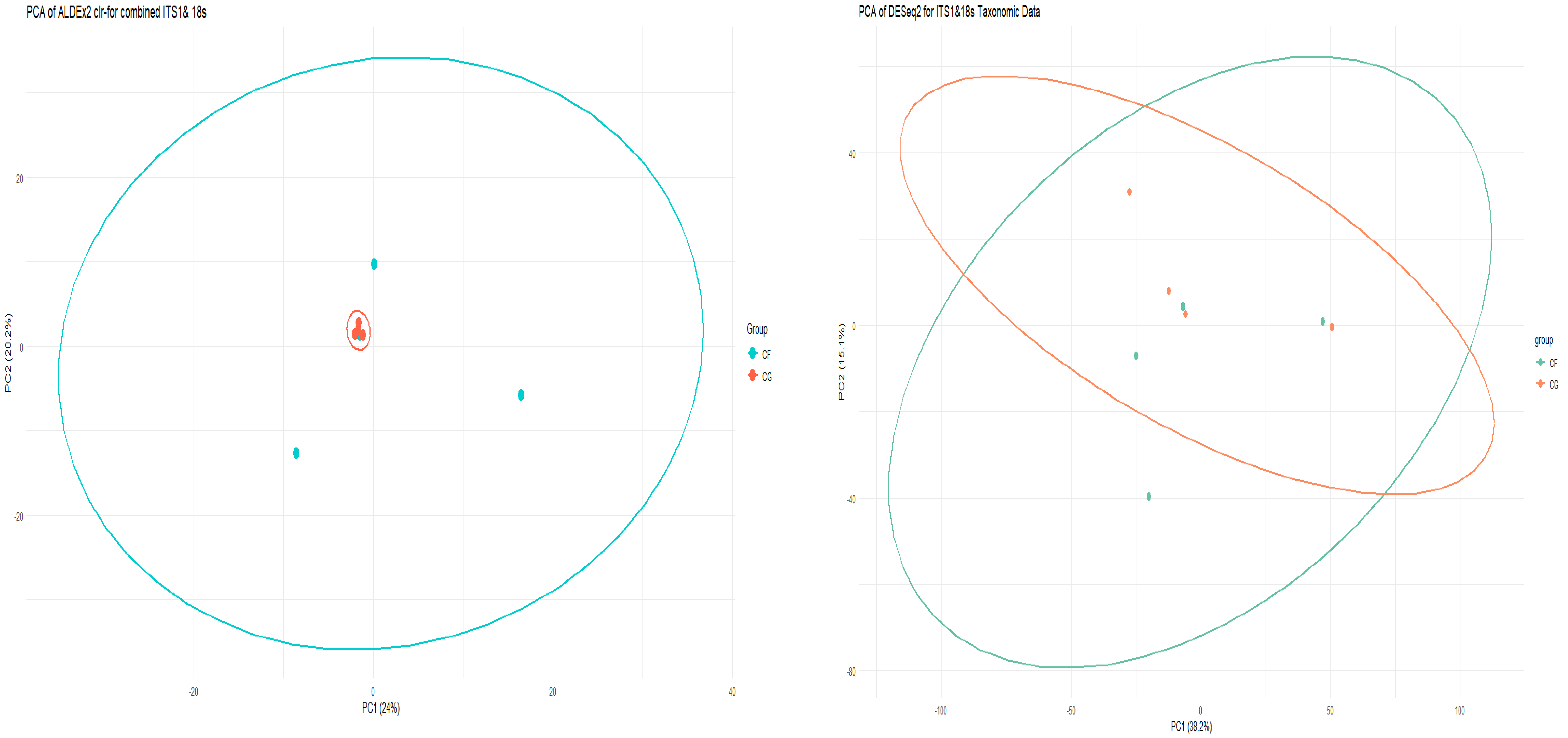

Figure 17: Principal Component Analysis (PCA) plots of combined datasets. Generated by ALDEx2 (*A*) and DESeq2 (*B*), illustrating the variance and clustering patterns among samples based on the first two principal components (PC1 and PC2), explained variance percentages. The plots' ellipses represent the group dispersion, visualizing group overlap or separation to highlight the differences or similarities in fungal community composition.

(A) (B)

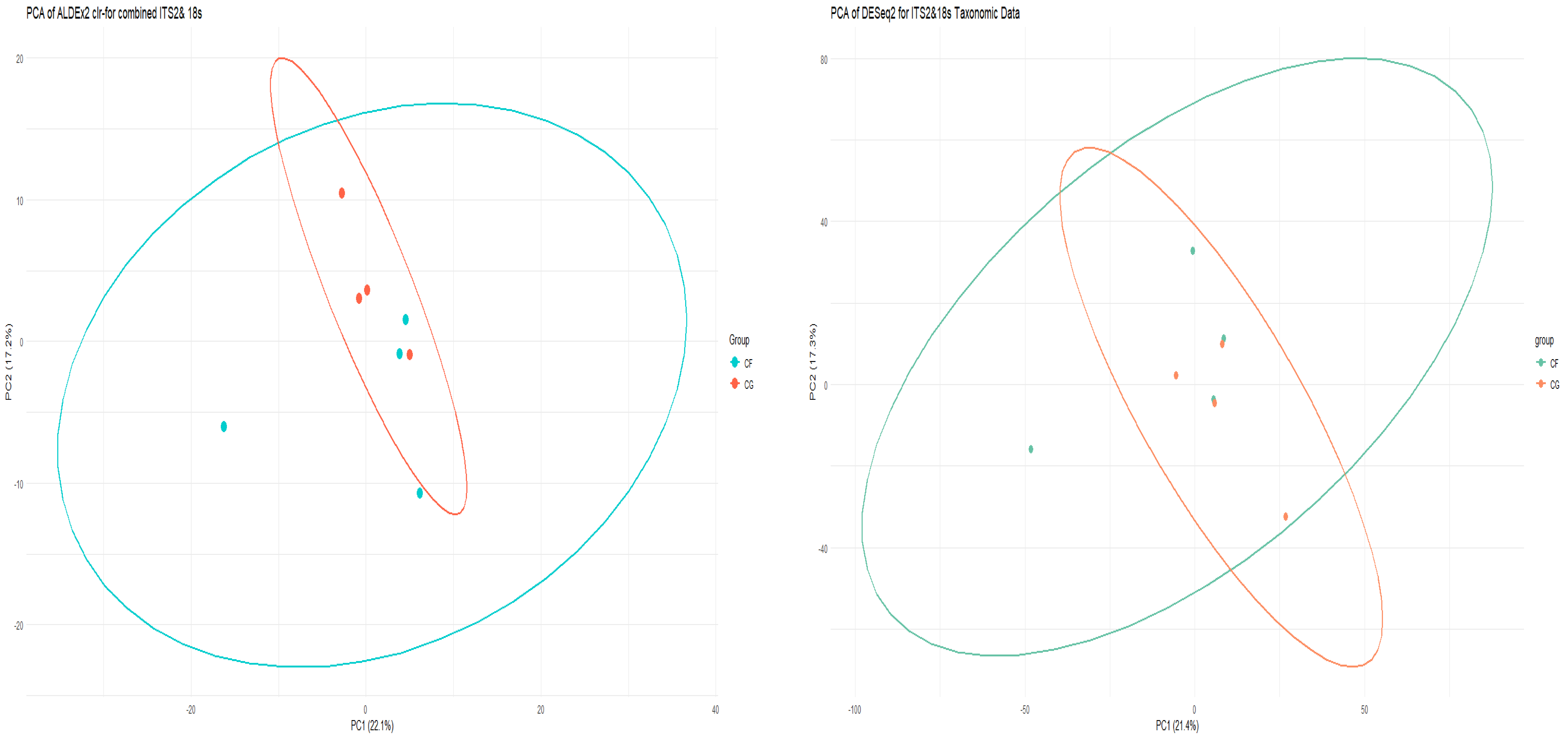

Figure 18: Principal Component Analysis (PCA) plots of combined datasets. Generated by ALDEx2 (*A*) and DESeq2 (*B*), illustrating the variance and clustering patterns among samples based on the first two principal components (PC1 and PC2), explained variance percentages. The plots' ellipses represent the group dispersion, visualizing group overlap or separation to highlight the differences or similarities in fungal community composition.
